# Concerted Evolution of Duplicate Control Regions in the Mitochondria of Species of the Flatfish Family Bothidae (Teleostei: Pleuronectiformes)

**DOI:** 10.1371/journal.pone.0134580

**Published:** 2015-08-03

**Authors:** Dong-He Li, Wei Shi, Thomas A. Munroe, Li Gong, Xiao-Yu Kong

**Affiliations:** 1 Key Laboratory of Tropical Marine Bio-resources and Ecology and Guangdong Provincial Key Laboratory of Applied Marine Biology, South China Sea Institute of Oceanology, Chinese Academy of Sciences, Guangzhou, 510301, China; 2 South China Sea Bio-Resource Exploitation and Utilization Collaborative Innovation Center, Guangzhou, 510301, China; 3 University of Chinese Academy of Sciences, Beijing, 100049, China; 4 National Systematics Laboratory NMFS/NOAA, Post Office Box 37012, Smithsonian Institution NHB, WC 60, MRC-153, Washington, D.C., 20013–7012, United States of America; Sichuan University, CHINA

## Abstract

Mitogenomes of flatfishes (Pleuronectiformes) exhibit the greatest diversity of gene rear-rangements in teleostean fishes. Duplicate control regions (CRs) have been found in the mito-genomes of two flatfishes, *Samariscus latus* (Samaridae) and *Laeops lanceolata* (Bothidae), which is rare in teleosts. It has been reported that duplicate CRs have evolved in a concerted fashion in fishes and other animals, however, whether concerted evo-lution exists in flatfishes remains unknown. In this study, based on five newly sequenced and six previously reported mitogenomes of lefteye flounders in the Bothidae, we explored whether duplicate CRs and concerted evolution exist in these species. Results based on the present study and previous reports show that four out of eleven bothid species examined have duplicate CRs of their mitogenomes. The core regions of the duplicate CRs of mitogenomes in the same species have identical, or nearly identical, sequences when compared to each other. This pattern fits the typical characteristics of concerted evolution. Additionally, phylogenetic and ancestral state reconstruction analysis also provided evidence to support the hypothesis that duplicate CRs evolved concertedly. The core region of concerted evolution is situated at the conserved domains of the CR of the mitogenome from the termination associated sequences (TASs) to the conserved sequence blocks (CSBs). Commonly, this region is con-sidered to regulate mitochondrial replication and transcription. Thus, we hypothesize that the cause of concerted evolution of the duplicate CRs in the mtDNAs of these four bothids may be related to some function of the conserved sequences of the CRs during mitochondrial rep-lication and transcription. We hope our results will provide fresh insight into the molecular mechanisms related to replication and evolution of mitogenomes.

## Introduction

The vertebrate mitochondrial genome (mitogenome) typically codes for 37 genes, including 13 protein-coding genes, 22 transfer RNAs (tRNAs), two ribosomal RNAs (rRNAs) and one control region (CR) [1]. However, two or more CRs have been found in some vertebrate mitogenomes, including those of birds [2–4], turtles [5–7], snakes [8–10] and fishes [11–13]. Moreover, there is a special phenomenon in mitogenomes with duplicate CRs, where sequences of the duplicate CRs are extremely similar. This has been proposed to have evolved in a concerted fashion, a situation which has been found in birds, snakes, fishes, sea cucumbers, sea firefly and ticks [3, 4, 8, 9, 12, 14–18]. Based on Liao’s opinion (1999), the concept of concerted evolution of duplicate CRs is a peculiar evolutionary phenomenon of the two CRs in mitogenomes, which may lead to homogenization of duplicate CRs within one species [19]. Shao *et al*. [17] further defined concerted evolution of CRs as including two main points: (1) the sequences of CR1 and CR2 of one species are highly similar; and (2) they are more similar to each other in the same species than either is to its namesake in the other species.

Molecular phylogenetic studies have made significant contributions to further understanding the process of concerted evolution of duplicate CRs in many different species [3, 8, 9, 12, 14]. For example, Eberhard *et al*. [15] conducted a phylogenetic analysis of the duplicate CRs from 21 individuals representing four *Amazona* parrots. The result revealed that two CRs of an individual were more closely related to one another than to corresponding segments of others in the same species, except for three subspecies in *Amazona ochrocephala*. The paralogous CRs of an individual in the same subspecies did not group together, but the orthologous CRs in different individuals clustered first. This phylogenetic analysis helps to understand the different evolutionary pattern of the two CRs, one was concerted evolution of the two CRs in some species and the other was independent evolution in other subspecies.

As more mtDNAs with duplicate CRs have been sequenced, different hypotheses explaining how duplicate CRs are generated and how they have evolved in a concerted fashion have been put forward [9, 16, 18]. Most of them explain for either the generation of CRs or for the concerted evolution of duplicate CRs, just one example could explain both cases.

Two mechanisms, illegitimate recombination and dimeric mitogenome, were used to account for the generation of duplicate CRs. The illegitimate recombination involves the breakage and rejoining of participating DNA strands [20], and if the recombined DNA strands include the CR, it would generate a mitogenome with two CRs. The dimeric mitogenome is formed by two monomeric mitogenomes joining head to tail (dimerization). This mitogenome would have two CRs and two sets of mitochondrial genes [21].

Another two mechanisms have been proposed to explain how duplicate CRs maintain concerted evolution. Ogoh and Ohmiya [18] illustrated that the duplicated CR1 inserted at the location where the old CR2 was deleted during mitochondrial replication, which made the sequences of the two CRs identical or highly similar. The mechanism of gene conversion proposed by Kumazawa *et al*. [9] indicated that the sequences of two control regions can be homogenized. The crossing over of nicked strands between two control regions within an mtDNA molecule leads to formation of a Holliday structure, and the sequence of one control region may be replaced by that of the other via repair of heteroduplex DNA intermediates.

The tandem duplication mechanism, proposed by Kumazawa *et al*. [9], account for not only the generation of duplicate CRs but also for the concerted evolution of them. It involves a replication error triggered by imprecise termination or slipped-strand mispairing, which would lead to duplication of a section. If this section included the CR, then the replication error would generate a mitogenome with two copies of the CRs.

Of the1500 complete mitogenomes available for teleosts from GenBank (as of November, 2014, http://www.ncbi.nlm.nih.gov/genome), duplicate CRs have been found only in three species, including *Kryptolebias marmoratus* (Cyprinodontidae) [12], a righteye flounder, *Samariscus latus* (Samaridae) [13], and a lefteye flounder, *Laeops lanceolata* (Bothidae) [22]. Only a single species from each of these families has been found with this phenomenon. The studies related to these species mainly focused on gene rearrangements, or on the evolutionary pattern conducted only in a single species; both situations represent only narrow taxonomic coverage. No concerted evolution of CRs has been reported in these species or in broad taxa above the level of species. Even within the entire group of teleosts, no phylogenetic study about concerted evolution of duplicate CRs has ever been reported before.

The family Bothidae (excluding species now assigned to the Paralichthyidae) has been recovered as a monophyletic lineage within the Pleuronectiformes in several studies [22–27]. Based on the previous finding of duplicate CRs in the mitogenome of *L*. *lanceolata*, here we sequenced five new mitogenomes from species in the family Bothidae to test whether or not duplicate CRs exist in these species. Also, together with six previously reported complete mitogenomes in this family, we’ll explore how duplicate CRs were generated and what the evolutionary pattern and molecular mechanism of CRs exist in this monophyletic lineage representing 11 species from seven genera. We hope our results will provide fresh insight into molecular mechanisms related to replication and the evolution of mitogenomes.

## Materials and Methods

### Ethics statement

This study was carried out in strict accordance with the recommendations and guidelines of the National Institutes of Health. No specific permits were required for specimens used in the present study because they are common marine fishes captured in commercial fisheries. *Arnoglossus tenuis* was collected from Shipu commercial fisheries, Zhejiang, China; *Chascanopsetta lugubris* and *Crossorhombus valderostratus* from commercial fisheries in Durban, South Africa; *Lophonectes gallus* from the Sydney Fish Market, Australia; *Psettina iijimae* from Xingda commercial fisheries, Kaohsiung, Taiwan. These species are not included in the endangered species list of the IUCN (http://www.iucnredlist.org/). All species are small- to moderate-sized species of flatfishes characterized by both eyes on the left side, with pectoral and pelvic fin rays not branched [28, 29].

### Sampling, DNA extraction, PCR amplification and sequencing

Mitogenomes for *A*. *tenuis*, *C*. *lugubris*, *C*. *valderostratus*, *L*. *gallus* and *P*. *iijimae* were sequenced for the present study and have been submitted to GenBank (Table 1). Total genomic DNA was extracted from the muscle of these specimens using an SQ Tissue DNA Kit (OMEGA, Guangzhou, China) according to the standard manufacturer’s protocol. Dozens of primer pairs (S1 Table) were designed for the fragment amplifications of five flatfish mitogenomes based on the method of aligning sequences of the flatfish mitogenomes previously reported by Shi *et al*. [30].

**Table 1:** Flatfishes used in this study.

Classification	Species	Abbreviation	LEF/REF*	Accession No.*	Duplicate CRs
Bothidae	*Arnoglossus polyspilus*	A.po	LEF	NC_024946	NO
	*Arnoglossus tenuis*	A.te	.LEF.	**KP134337**	YES
	*Bothus myriaster*	B.my	LEF	KJ433563	NO
	*Bothus pantherinus*	B.pa	LEF	NC_024947	NO
	*Crossorhombus azureus*	C.az	LEF	JQ639068	NO
	*Crossorhombus kobensis*	C.ko	LEF	NC_024949	NO
	*Crossorhombus valderostratus*	C.va	LEF	**KJ433566**	NO
	*Chascanopsetta lugubris*	C.lu	LEF	**KJ433561**	NO
	*Lophonectes gallus*	L.ga	LEF	**KJ433567**	YES
	*Laeops lanceolata*	L.la	LEF	AP014591	YES
	*Psettina iijimae*	P.ii	LEF	**KP134336**	YES
Paralichthyidae	*Paralichthys olivaceus*	P.oli	LEF	NC_002386	NO
Pleuronectidae	*Platichthys stellatus*	P.ste	REF	NC_010966	NO

*: LEF/REF means Lefteye or Righteye flounder. The underlined nunber indicates newly-sequenced mtDNAs for five species of Bothids.

PCR was performed in a 25 μl reaction volume containing 2.0 mM MgCl_2_, 0.4 mM of each dNTP, 0.5 μM of each primer, 1.0 U of Taq polymerase (Takara, China), 2.5 μl of 10x Taq buffer, and approximately 50 ng of DNA template. The amplification profile included an initial denaturation at 95°C for 3 min, 35 cycles of a denaturation at 94°C for 45 s, an annealing temperature of 45–55°C for 45 s, and elongation at 68–72°C for 1.5–5 min. The PCR reaction was completed by a final extension at 72°C for 5 min. The PCR products were detected in 1.0% agarose gels and purified with the TaKaRa Agarose Gel DNA Purification Kit (TaKaRa, China) and used directly as templates for cycle sequencing reactions in both directions with the ABI 3730 DNA sequencer (Applied Biosystems, USA). Sequence specific primers were further designed and used as walking primers for both strands of each fragment.

### Sequence analysis

All sequenced fragments were assembled to create complete mitochondrial genomes using CodonCode Aligner (version 3.7.1) and BioEdit (version 7.0.1) [31], and then manually checked against possible sequencing errors. Annotation and boundary determination of protein-coding genes, rRNA and tRNA genes were performed using NCBI-BLAST. Identification of the CR is based on identifying the symbolic structures of CRs: Conserved Sequence Blocks (CSBs) and Termination-Associated Sequences (TASs) [30]. Tandem repeated sequences (TRs) were identified using Tandem Repeats Finder 4.03 in the default parameters. The genetic distances of Kimura-2 Parameter between the core region sequences of CRs were calculated in the MEGA 5.0 program [32].

### Phylogenetic analysis and ancestral state reconstruction

Eleven mitogenomes of species of Bothidae, five from the present study and six others retrieved from Genbank, were used for phylogenetic analyses. Based on previous studies [22, 24–26], two species, *Platichthys stellatus* (Pleuronectidae) and *Paralichthys olivaceus* (Paralichthyidae), which have a close relationship with bothids, were chosen as the outgroup (Table 1). Two datasets were generated: one included the first and second codon position sites for each of 12 coding genes (without the third codon position site and the *ND6* gene following studies by Campbell *et al*. [22]), the rRNA genes (R), and tRNA genes (T) (noted as 12RT, S1 Fig); the other dataset included only the core region of CRs excluding the tandem repeat sequences (S2 Fig). Before phylogenetic tree estimations, multiple alignment within each dataset was performed using Clustal X [33], then the two datasets were used for maximum likelihood (ML) analyses implemented in PhyML [34] and Bayesian inference (BI) in MrBayes 3.1.2 [35]. The support values for ML analyses were evaluated with 1000 non-parametric bootstrap replicates and the best-fit evolutionary models were determined using Modeltest 3.7 [36]. The best-fit models of nucleotide substitution for the BI analyses were selected with MrModeltest 2.1 [37] and the BI trees were constructed using MrBayes ver.3.2.1 on 10,000,000 generations and 100 sampled generations [35]. The phylogenetic signal was tested based on the existence of CRs in 11 bothids with two other flatfishes as outgroup using the commonly employed metrics: Pagel’s λ [38]. Calculations of Pagel’s λ were conducted using the R package Geiger [39–41]. The ancestral state was reconstructed by using maximum likelihood in the Ape package of the R statistical environment [40, 41].

## Results and Discussion

### Genome organization

**Fig 1:**
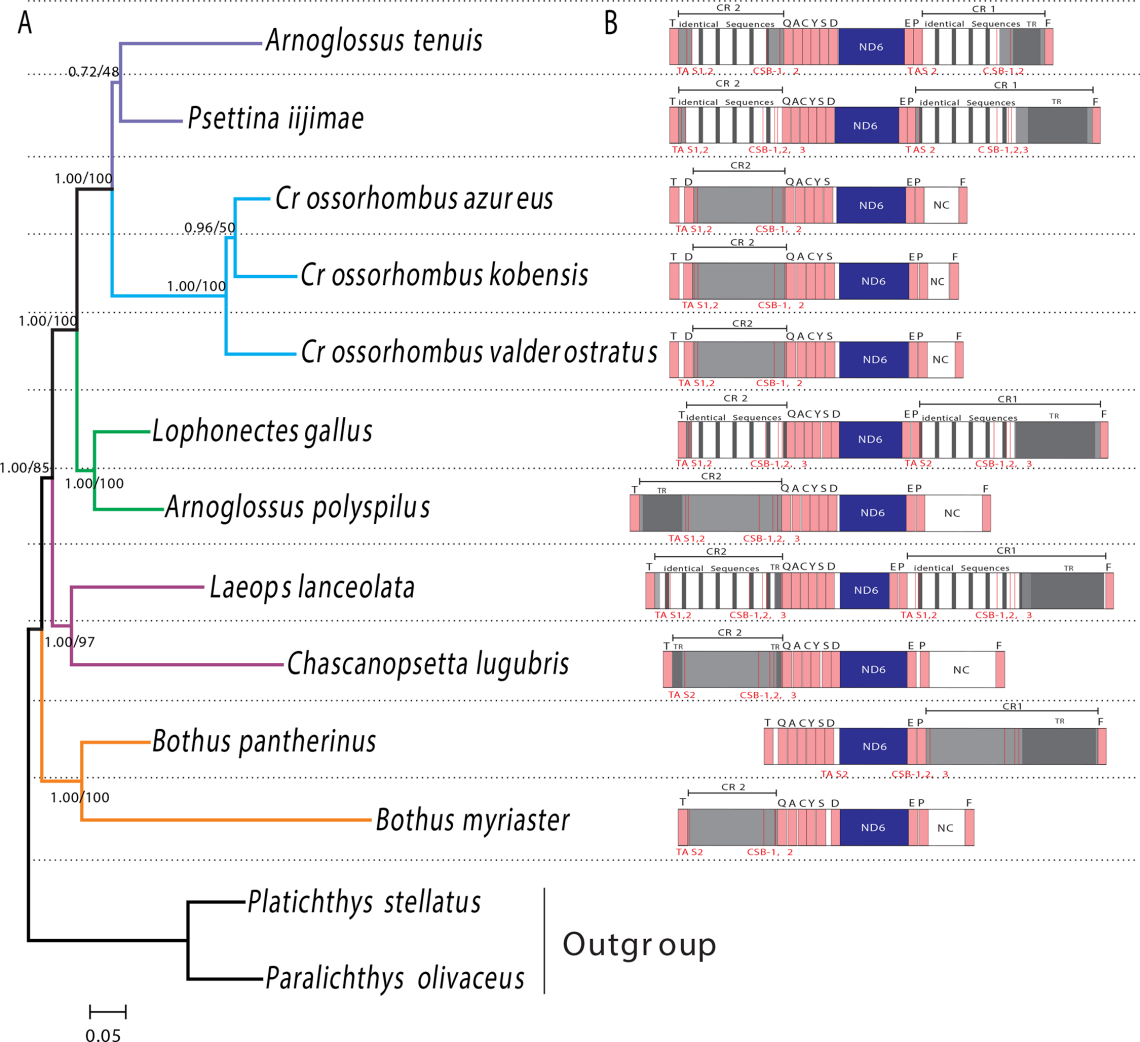
(A) Phylogenetic tree for 11 bothids with two other flatfishes as outgroup using the 12RT dataset. The phylogenetic tree was reconstructed by maximum likelihood and Bayesian methods. The first number beside the internal branch indicates Bayesian posterior probability, the second one is the bootstrap value. (B) Gene orders of fragments between tRNA-T and tRNA-F from 11 bothid species. Cross-hatched area in bold indicates the core region of duplicate CRs. Light-gray box indicates the Conserved Sequence Blocks (CSBs) and Termination-Associated Sequences (TASs); Dark-gray box indicates the Tandem Repeat Sequences (TRs); White box indicates the Non-coding region (NC).

The mitogenomes of *A*. *tenuis*, *C*. *lugubris*, *C*. *valderostratus*, *L*. *gallus* and *P*. *iijimae* were all circular molecules, with 17,556 bp, 17,251 bp, 16,790 bp, 18,642 bp and 18,080 bp in length, respectively. All of them contained 37 genes, including 13 protein-coding genes, two rRNA genes and 22 tRNA genes. Most of these genes were encoded on the heavy-strand, while *ND6* and eight tRNA genes were encoded on the light-strand (S2 Table). All tRNA genes could be folded into typical cloverleaf structures except for the *tRNA-C*. Two large non-coding (NC) regions were found in these five species, one was located between *tRNA-P* and *tRNA-F*, the other was situated between *tRNA-T* and *tRNA-Q*. Comparison of these NC sequences with the CR sequences of other flatfishes revealed that *C*. *lugubris* and *C*. *valderostratus* had one CR located between *tRNA-T* and *tRNA-Q*, with lengths of 855 bp and 704 bp, respectively. However, *A*. *tenuis*, *L*. *gallus* and *P*. *iijimae* had two similar control regions, which were termed CR1 (between *tRNA-P* and *tRNA-F*) and CR2 (between *tRNA-T* and *tRNA-Q*). The length of CR1 ranged from 966–1,513 bp and CR2 ranged from 830–836 bp (S2 Table). The length differences between the CRs mainly resulted from the presence of tandem repeats with extensive variation in sizes and copy numbers (Fig 1A and 1B).

### Concerted evolution of duplicate CRs

Comparisons of six previously determined mitogenomes of bothid species [22, 30, 42], reveal that only one species, *L*. *lanceolata*, has duplicate CRs (Table 1). Together with three other bothids in the present study, a total of four species from different genera in the Bothidae have duplicate CRs (Fig 1A and 1B). The core region sequences of duplicate CRs of mitogenomes within the same species were virtually identical or nearly identical (represented by cross-hatched areas in Fig 1B, about 600 bp in total). Sequence similarity of the core regions of the paralogous CRs (CR1 and CR2) was 99.8–100% in the same species, versus 65.4–76.1% between counterparts of the orthologous CRs in different species (S3 Table, Fig 1B and S2 Fig). Genetic distances of the Kimura-2 Parameter between the core regions of the paralogous CR1 and CR2 are 0–0.2%, versus 23.8–43.6% between the counterparts of the orthologous CRs (S3 Table, S2 Fig). The high similarity and small genetic distances between duplicate CRs within each of four species fit the predicted pattern of concerted evolution [3, 15, 17, 18].

**Fig 2:**
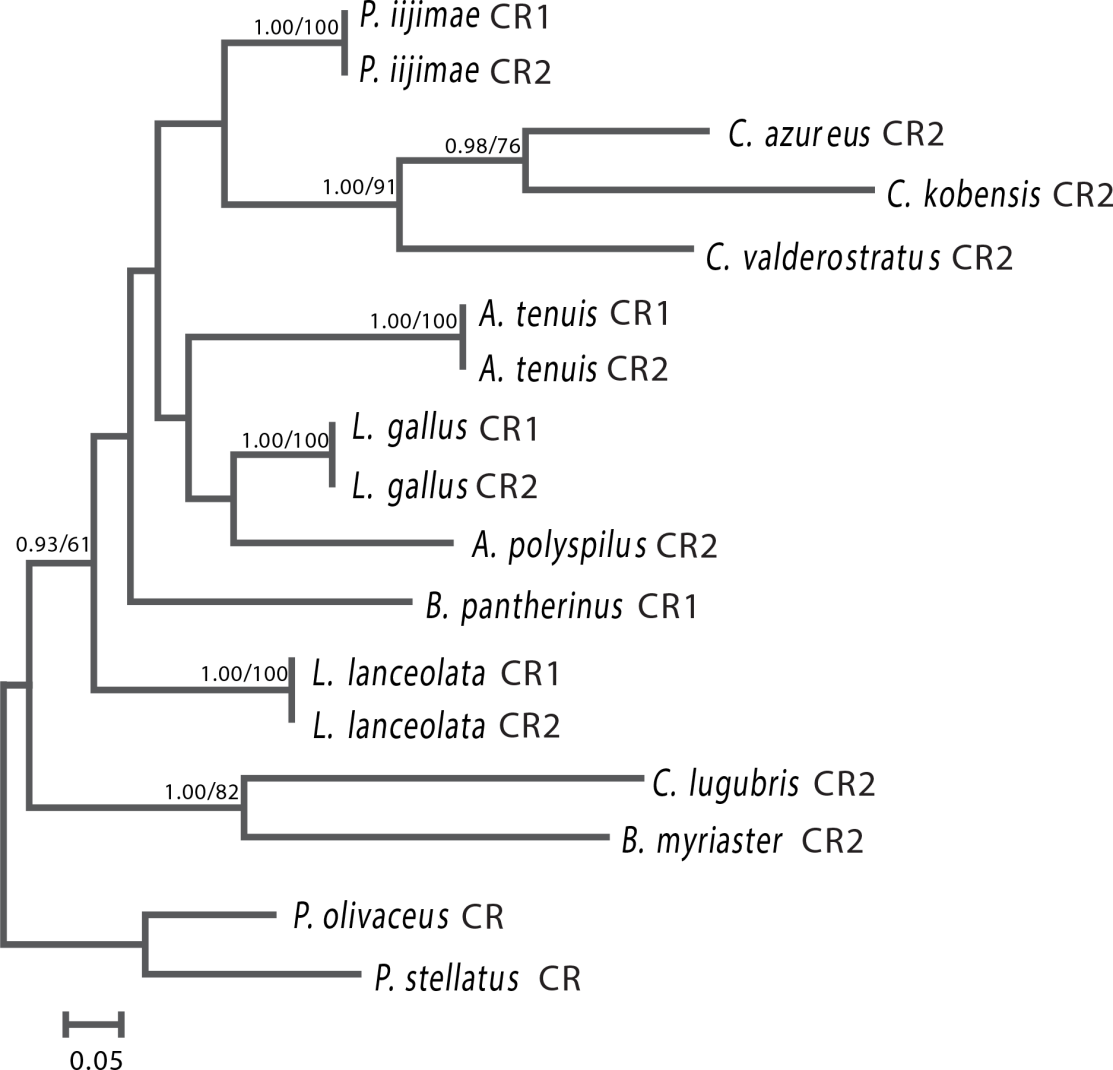
Phylogenetic tree for 11 bothids with two other flatfishes as outgroup using the core region of CRs. The first number beside the internal branch indicates Bayesian posterior probability (values below 0.9 not shown); the second number represents the bootstrap value (values below 50% not shown).

Based on two datasets (12RT and CRs) from 11 bothid species with two other flatfishes as outgroup, ML and BI trees were constructed. The results showed that both trees were largely congruent with each other, therefore, only one topology with both support values was shown, including bootstrap values for the ML tree and posterior probability for Bayesian analysis (Figs 1 and 2). Phylogenetic analyses supported the hypothesis that all 11 species were the descendants of the monophyletic group of Bothidae, but the four flounders with duplicate CRs did not group to one clade. However, the CR1 and CR2 from one species always clustered together (Figs 1 and 2). These results indicated that the four flounders with duplicate CRs are not monophyletic, and that the concerted fashion of duplicate CRs is not a single event that happened only in one lineage of bothid, but rather, it occurred in multiple cases within the family Bothidae.

### How are duplicate CRs generated?

So far, the phenomenon of four species with duplicate CRs from different genera within one family (Bothidae) is the first report in fishes. How these duplicate CRs were generated raises a very interesting question. Comparison of the mechanisms for the generation of duplicate CRs from previous reports indicates that the similar dimerization model is suitable to explain the cases in these four species, which is similar to the conclusion of Shi *et al*. [30] who used this mechanism to describe the process for generation and degeneration of duplicate CRs in the mitogenome of *Crossorhombus azureus*.

The complete mitogenome of *C azureus*, the first one for species of lefteye flounder, has undergone genomic-scale gene rearrangements. The *ND6* and seven tRNA genes (*Q*, *A*, *C*, *Y*, *S1*, *E*, *P*) encoded by the light-strand have been translocated to the position between *tRNA-T* and *tRNA-F*. It also contains two large non-coding regions (NC1 and NC2), one (NC2) is identified as the CR, the other (NC1) is considered as a remnant from one of the duplicate CRs. Considering the mechanisms previously used to explain the generation of duplicate CRs, Shi *et al*. [30] point out that the tandem duplication mechanism and illegitimate recombination model were not as parsimonious as the dimeric mitogenome model [21]. Therefore, they inferred that the duplicate CRs were generated from a dimeric molecule of mitogenome formed by two monomers linked head-to-tail. Subsequently, one of the CRs was degraded to the NC1 and the other became the new CR [30].

For 11 lefteye flounders in this study, the mitogenomes of four species have the two CRs (CR1 and CR2), whereas seven others have only one CR and in the position where a second CR would occur (Fig 1B), the mitogenome has a large non-coding (NC) region. Further analysis revealed that either the position of the NC or the CR, as well as the cluster of light-strand coding genes, is consistent with the arrangements observed in *C*. *azureus* (Fig 1B; S2 Table). Thus, we hypothesized that the duplicate CRs of four bothids were generated through the same mechanism outlined in the dimerization model and proposed for the evolution of the mtDNA of *C*. *azureus* as was described by Shi *et al*. [30].

### What is the cause of concerted evolution of duplicate CRs in bothid species?

The evidence of duplicate CRs (CR1 and CR2) in four species and a large non-coding region in seven species with one CR supports the hypothesis that the mitogenomes of seven species once had the other CR at the corresponding position of NC [30]. That is, the mitogenomes of 11 bothid species in the ancestral state have two CRs. Accordingly, we hypothesize that duplicate CRs of the mitogenomes in these flounders are inherited from a common ancestor of present-day bothids.

**Fig 3:**
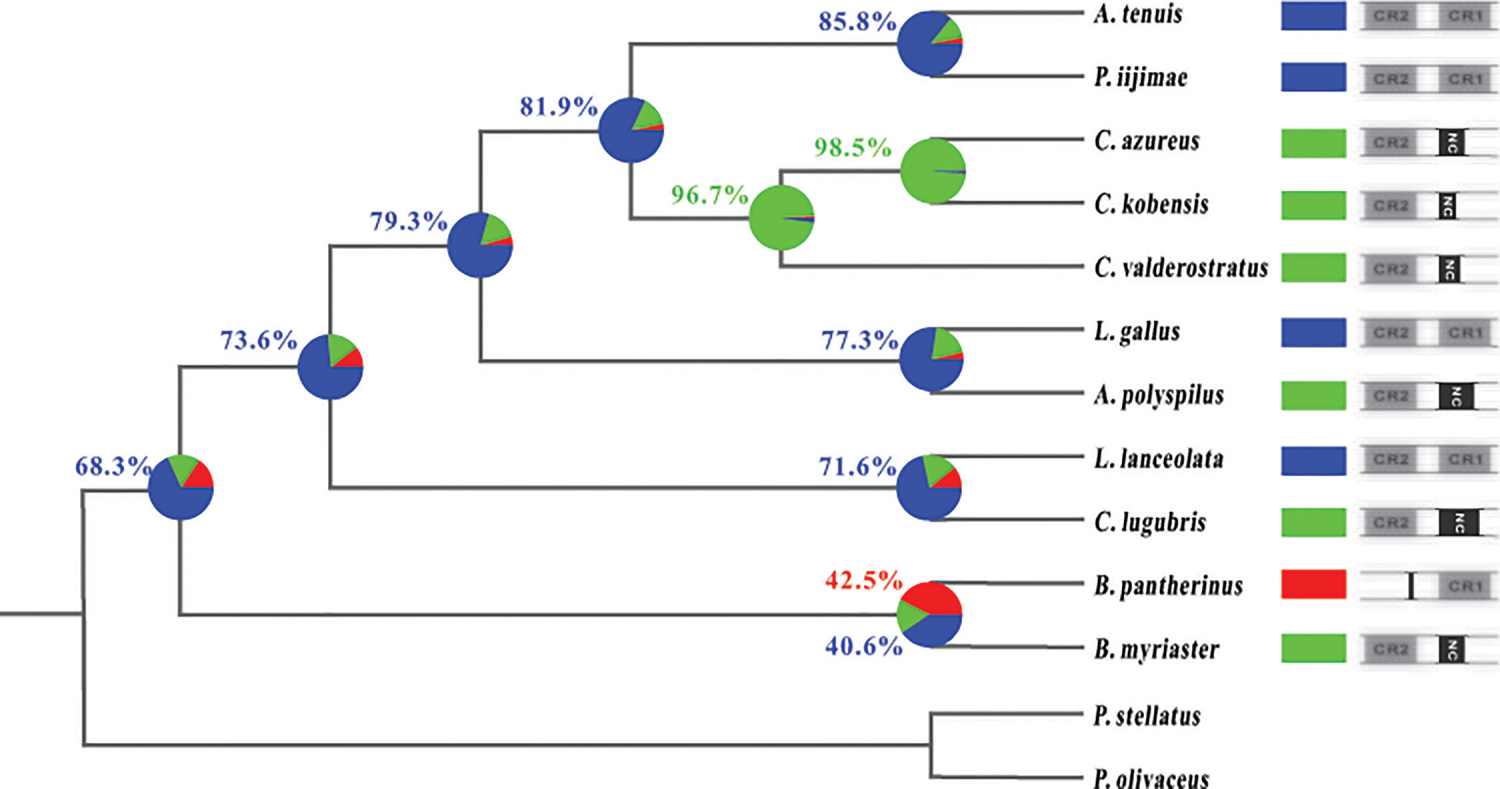
The analysis of ancestral state reconstruction for CR type based on maximum likelihood approach. The pie chart represents the relative likelihood value of alternative CR type. Numbers in color represent the relative likelihood value of the corresponding slice (values below 40% not shown). The blue slice indicates the likelihood that both the CR1 and CR2 were inherited from a common ancestor; the red slice indicates the likelihood of CR1; the green slice indicates the likelihood of CR2. NC represents the non-coding region that was degenerated from the CR.

**Fig 4:**
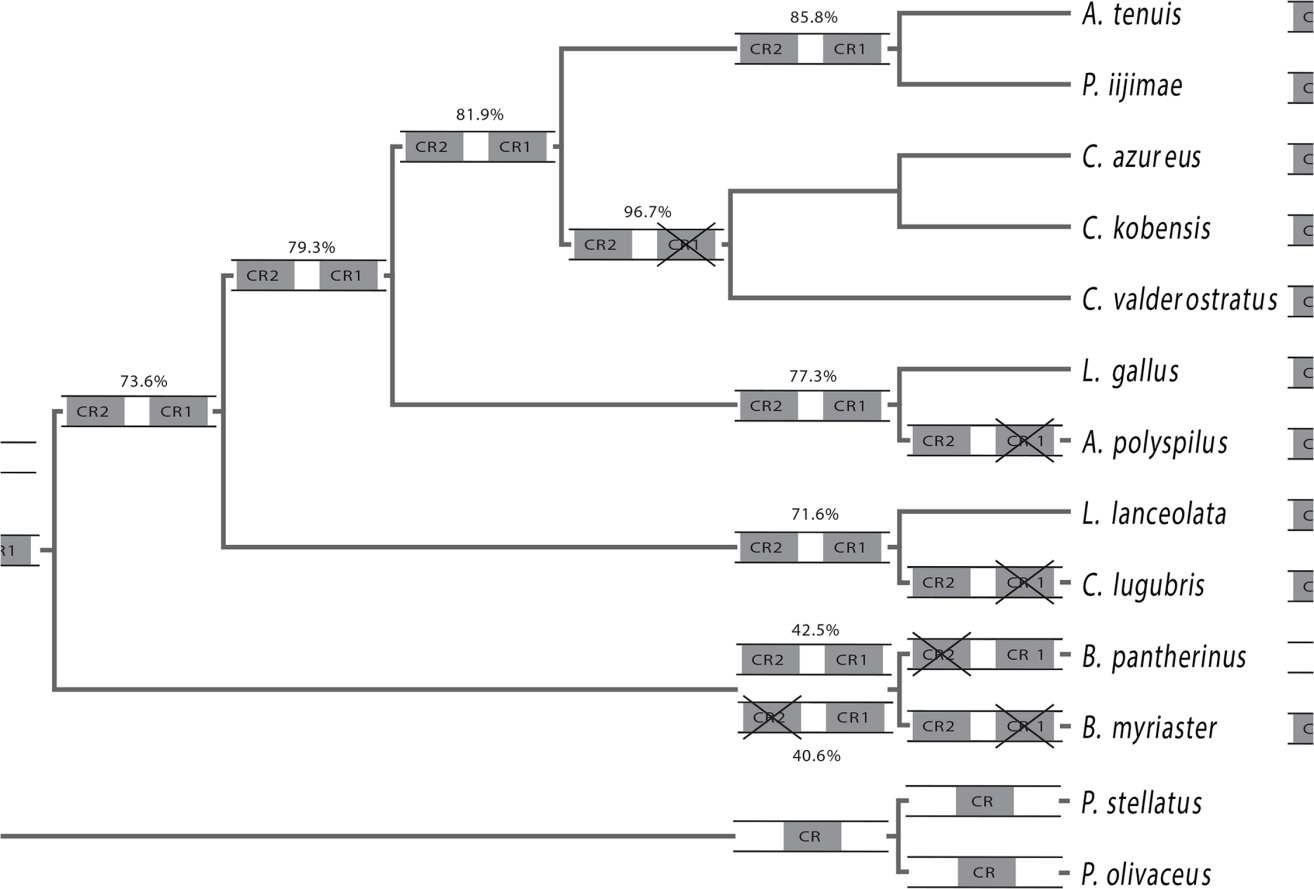
The hypothesized evolutionary mode of duplicate CRs of 11 bothids with two flatfishes as outgroup. The mode was hypothesized based on the highest likelihood values of the ASR result. Number and CRs status beside the nodes are based on the highest likelihood value of the ASR result. The two close likelihood values of 40.6% and 42.5% for the node including *B*. *pantherinus* and *B*. *myriaster* are shown.

In order to provide more evidence for this hypothesis, we reconstructed the ancestral state of the CRs character based on the existence of the CRs from 11 bothids with two other flatfishes as outgroup. First, we calculated the phylogenetic signal by Pagel’s λ. Pagel’s λ is a scaling parameter that ranges from 0 to 1.0 Lambda values. Lambda value of 0 indicates no phylogenetic signal, whereas a value of 1.0 indicates perfect phylogenetic signal. The result of Pagel’s λ in this analysis was a 1.0 Lambda value that indicated perfect phylogenetic signal. Therefore, the ancestral state reconstruction (ASR) of the CRs character was deduced by using the maximum likelihood approach (Fig 3). From the highest likelihood values of the ASR result, the evolutionary mode of duplicate CRs was plotted out manually (Fig 4).

The ASR result showed that the copy of CRs had already occurred as early as the origin of the bothids. With expansion of bothid lineages, the duplicate CRs have been retained in some such as *Arnoglossus* (*Arnoglossus tenuis*), *Lophonectes*, *Laeops* and *Psettina*. Meanwhile, in the evolution of other lineages, i.e., *Crossorhombus*, *Arnoglossus* (*Arnoglossus polyspilus*), *Chascanopsetta*, CR1 was degraded and only CR2 was retained. In the *Bothus* lineage, there are two different evolutionary results: one branch lost CR1 and the other kept CR1, but lost CR2. This result supports the hypothesis that the two CRs have the same evolutionary tempo and direction, and it also provides other evidence for our hypothesis that the ancestral state in these bothid species has two CRs (Figs 3 and 4). Furthermore, it also shows that duplicate CRs do not result from recent evolutionary events in these fishes, but rather, they appeared as early as the origin of the bothid lineage (Figs 3 and 4). Subsequently, during evolutionary expansion of bothid lineages from two CRs ancestral state, one of the duplicate CRs was lost in different bothid species at different evolutionary times, whereas, in contrast, other species (lineages) still retained two CRs.

As for the time of origin of a common ancestor of present-day bothids, studies have discovered that bothids may have appeared about 30 million years ago (mya) based on calculations of the molecular clock [26], or to the late Eocene (ca. 40–56 mya) based on fossil evidence [43].

Over at least 30 mya of evolution, how did those mitogenomes keep two CRs without degeneration and how did duplicate CRs maintain concerted evolution in their core regions? Comparisons with the above three mechanisms about concerted evolution of CRs (the tandem duplication model [9], the deleted and duplicated hypothesis [18] and the gene conversion [9]), revealed that all of them are not suitable here. Primarily, because the first two models hypothesize that concerted evolution of CRs is maintained by generating new duplicate CRs through multiple independent origins. With respect to the gene conversion mechanism [9], it is mainly caused by homologous recombination. It is known that recombination is rarely found in the mitogenomes of teleostean fishes. Based on the phylogenetic trees, the four species of bothids possessing concerted evolution of their CRs belong to different branches rather than one lineage. If the events of concerted evolution of two CRs resulted from one of the three mechanisms mentioned above, the same replication errors should have happened several times in different species; the possibilities of these cases are too low to occur because the mitogenome structure is quite conserved and relatively stable. In addition, Shao *et al*. [17] also considered that it is unlikely that the same replication errors (tandem duplication model) arise over and over again, and independently in each species.

Although the mechanism of concerted evolution of CRs in the Bothidae is not quite sure, further analysis of the structure of CRs of four bothids having concerted evolution can still reveal useful information. The symbolic structures of the CR in vertebrate mitogenomes contain some conserved sequences known as CSBs and TASs. The fragment from the TASs to CSBs is generally considered to be the core region for regulation of mitochondrial replication and transcription of the heavy-strand [44–48]. The CSBs play a crucial role in both transcription and replication since they are required for the stability of heavy-strand synthesis initiation [49], and the TASs have a function that can arrest heavy-strand synthesis [47, 48].

In the mitogenomes of the four lefteye flounders with duplicate CRs, the concerted evolution region (identical or nearly identical sequences) in two CRs of each species is restricted in these conserved sections. Although some sequences are very close to these regions, such as the tandem repeat sequences in the mitogenome of *L*. *gallus*, which has only an 11-base-pair interval to the concerted evolution region of CR1 (Fig 1B), concerted evolution was not found in them because those tandem repeat sequences are not present in CR2. When Eberhard *et al*. [15] studied the concerted evolution of the CRs in parrots, they also pointed out that duplicate CRs may persist only if the duplication event gives rise to complete, functional copies. Thus, we infer that concerted evolution of the CRs in these bothid species may be related to some function of the conserved sequences of the CRs during mitochondrial replication and transcription.

## Supporting Information

S1 Fig Alignment of the sequences of 12RT dataset from mitogenomes of 13 species of flatfishes. Abbreviations of species names are shown in Table 1.
(DOCX)

S2 Fig Alignment of the core region sequences of the CRs from mitogenomes of 13 species of flatfishes. Abbreviations of species names are shown in Table 1. Numbers following species names represent the types of the CRs: CR1 or CR2. “1” represents the CR1, and “2” represents the CR2, respectively.
(DOCX)

S1 Table Primers used for fragment amplifications in five flatfish mitogenomes. (DOCX)

S2 Table Features and gene maps of the mitogenomes of five species of Bothidae. Intergenic region: non-coding bases between the feature on the same line and the line below, with a negative number indicating an overlap.
(DOCX)

S3 Table Percent similarity (above triangle) and genetic distance (below triangle) calculated by Kimura-2 Parameter based on core regions of CRs and counterpart sequences from 11 bothids with two other flatfishes as outgroup. Abbreviations of species names are shown in Table 1. Numbers following the species names represent the type of CRs: CR1 or CR2. Numbers in gray are sequence similarity and the genetic distances of Kimura-2 Parameter between core regions of the duplicate CRs within each of four species of flounders.
(DOCX)
