# Supplementary material for: Concerted Evolution of Duplicate Control Regions in the Mitochondria of Species of the Flatfish Family Bothidae (Teleostei: Pleuronectiformes)
Source: PLoS One. 2015 Aug 3;10(8):e0134580. doi: 10.1371/journal.pone.0134580 (PMC4523187; doi:10.1371/journal.pone.0134580)
Supplement: S1 Fig — Abbreviations of species names are shown in Table 1. (DOCX) [file pone.0134580.s001.docx]

A.te ATTGATGCAA GAACCAAAGC GCCGGGTCAC AGCAACGTAC CCAGCAAGGC TGCTCCAGGG GATTAAGCGC CAATTCCCTT TGCCCGCACG CACTGCACCC

P.ii .C.A.AC..C A........T .T.....T.. .......... .......... .......... ..C....... .T....A..C CA........ ......C...

C.az .CCA.AAT.C ....T..... .......... .......... .......... ..A....T.. .......... .T....A..C GA........ ..........

C.ko .CC..AAT.C AG..T..... .......... ....G..... .......... ..G....... ..C....... .T....GT.C GA........ ......C...

C.va .GAA.G.T.C .C..T..... .......... .......... .......... ..A....... .......... .T....A... GA....T... ......C...

L.ga .ACCCAAT.C C....GG... .......... ....GT.... .......... ......C... C.C.....A. .G....G..C CA........ .G....C...

A.po .CGCTAC..C C....GG... .......... .......... ......G... ..A....... ..C.....A. .T....GT.. CA.A....T. ......C...

B.pa .A.TCACT.C AC...GG... .........T ....GT.... ......G... ..A....... ..C.....C. .T....G..C .A........ ..........

L.la -AAATAC..C AC..TG.... .......... C......... A.....G... ..GC..C... C.C.....A. .T.G..G..C GA.A...... ..T.......

P.oli --AATACA.C A...TGC... .........T C...G..... .......... ...C...... A.C....... .T.G..G..C ...A...... ..........

P.ste --AATACA.C A....GT... .T........ C...G..... .......... ...C...... C.C....... .T.G..GT.C G..A....T. ........T.

C.lu G.GATAC..C C....G.... .......... .......... .......... ..G...T... A.C.C..... .CG...GT.A C..A...... .CA...C...

B.my .AACCACA.T CG..TGCT.T .......... G...G..... ......GC.. C.AC..G... ..C.....A. .TG...G..A .A.....G.. .G..A.C...

A.te CGCAGCACAA CTACGATATG GTAATGTGTG TATATGCAGG CCGGGCAATT GGACGCACCT AAATGGGTTC CGTGAGCGAA TCGTCGTCCG GATTGTACGG

P.ii .C.G...... .......... ..T.C..... .......... .......... .......... G...A..... .......... ..C....... ..........

C.az .C...T.... ........C. ..T.C..... .......G.. .......... .....T.... G...A....T .......... ..AC....T. A.........

C.ko .C.G.T.... ..G.....C. ..T....... .......... .......... .......... G...A..... .........G .......... ..........

C.va .C.G.T.... ...T....C. ..T.C..... .......G.. .......... .....T.... ....A.A... .......... ..C..A.... ...G...A..

L.ga .C........ ........C. ..T.C..... .......G.. .......... .......... ..G....... .......... .......... .......T..

A.po .C.G...... .......... ..T.C..... .......G.. .......... .......... G...T..... ...A.....G ..CC...... ....A.....

B.pa .C.G...... ..G.....C. ..T.C..... ......TG.. .......... .......... G.G....... .......C.. ..CC..C... ..........

L.la .C.....T.. ..G.....C. ..T.C..... .......... T......... .......... ......T... .......C.G .TCC...... ..........

P.oli TTAG...TG. T.G..C..C. ..T.C..... .......G.. .......... .......T.A ...CA...A. .A.A....GG .......... ....A.....

P.ste TCGG....G. T....C..C. ..T.C..... .......G.. .......... .......T.A ....A...A. .A.A....G. ...G...... ....A.....

C.lu .C.GA....C ........C. ..T.C..... .......G.. .......... .......... G......... .......... .......... A.........

B.my TCTG.....G ..G..C..C. ..T.C.G... .......... .......... .......A.. GGG.T....A .A.A...C.. ..CC..C... T.....T...

A.te AACTCTGTGG GGTGCGTGTA GGTGGGCTGC TTTGGTGAAC TAAGCAGGTT CGACCTCAAC GCGTGAAAAA CGGCGGCTCT ACGGTCCTCT GCCACCATAG

P.ii .......C.. .......... .......... ..C....... ........C. ...TTG.G.. A.A....... T.....G... .......... ..........

C.az .......C.. .......... .......... ..C....... .......AC. ..TATCTT.. .TA.A...C. A..T..G... .......... ..........

C.ko .......C.. .......... .......... ..C....... .......AC. ..TATC.TG. ..T..G..C. .A.T..G... .......... ..........

C.va .......C.. .......... .......... ..C....... .......AC. ..TATC.T.. .G.A....T. ..TA..G... .......... ..........

L.ga .......... .......... .......... ..C....... ........C. ...TAA.... AA..A...C. ...T..G... .......... ....A.....

A.po .......C.. .......... .......... ..C....... .......A.. ...A.A..C. AT......G. .......... .......... ....A....A

B.pa .......C.. .......... .......... .......... .......... ...TTG.TC. TG......G. .AA..CG.T. G......... ..........

L.la .......C.. .......... .......... ..C....... ........C. ...A.G.TT. AA......T. ..C..AG... .......... ..........

P.oli ...G...CA. .......... .........T ..C...T... ...ATT.... ..GT.A...G A.AGA.C... G.C.T.G... G..T...... .........A

P.ste ...G...CA. .......... .......... ......T... C....T.AC. ..GT.A.T.. .TAAA..... G.C.T.G... G..T...... .........A

C.lu .......C.. .......... .........T ..C....... .......AC. ...TAC.T.. .AA.A...C. .CC..CG... .......... .........T

B.my ...A....A. .......... .......... ..C....... ....T..AC. ...ATC.GG. TAAA.T.G.. .A.T.AG... ...T...... .........T

A.te TCCCATGCCC TCCGCCAGCG TCCC-TAACT ATTACCAGCG TGAAAATGAT CTTCGTAGCG AAACGAACCA GCACCCCGGG GGAATTCACG GGGGACCTGA

P.ii .....C.... ......C... .T..-A.... .C.G...... C......... ..C....... ......C... ..C....... .......... ..........

C.az .....C.... ........A. ....-A.... .C.G....T. C...C..... ..A....... ......G... ..T....... .......... ....G.....

C.ko .....C..T. ........A. ....-A.... ...G...... C...C..... ..A....... ......C... ..C....... .......... ..........

C.va ....G..... ........G. .T..-A.... .C.G...A.. ....C..... ..AT...... ......C... ..C....... .......... A...G.....

L.ga .T...C..T. ..T....... G...CA.... .C..AA.... C......... ..C....... ......C... ..C....... .......... ..........

A.po .T...C...A .......... ....T..... .CC..A.... C...C..... ..C....... ......G... ..C....... .......... ..........

B.pa .....C...A ......GAA. ....C..... .....A..T. A......... ..C.....T. ......G... ..C....... .......... ..........

L.la .....C.T.G .......AA. ....CG.... G.....G... C..GG..... ..C....... ......C... ..C.A..... .A.....G.. .........G

P.oli .....C..AA ..T....... G...CC.... .CA...C... C......... ..CT...... .......... ..C....... .A....T... A.........

P.ste .....C..GA ..T.....T. G...CC.... ..A...T.T. C...C..... ..C....... .......... ..C....... .A....TG.. A.........

C.lu ...TGC.... ..T...CTT. ....CGG... ....T..... C......... ..C...C... ...T..C... ..C....A.. .T.....G.. A.........

B.my .....C.TTA ..T.G.GAA. ....CC.... .C....GAA. C.....GA.. ..C....... .G.T..G.T. .......... ..G...AG.. A........G

A.te AAAGTAAGCT GAGCCACCTC CGCGTAGAGC AAGCGCGCTT CCGGCCGCGC AAGCGTTATC ACGCTGCCCC CCATTTGTCC TAAGATTACG GGTGGTACAC

P.ii .......... ..T.AT.... .A..GG.T.. .......... .......... ....A..... ........T. ...CCA.A.. CC..CC.... .....C....

C.az ......T... ..TG.T..A. .AA.AC.C.. .......... T........T ....AC.... ........A. ...CCATAT. CG...C.... ..........

C.ko ......T... A.TG.T..G. ..A.AG.... ...T...... .........T ....AC.... ........A. ...CCATG.. C....C.... ..........

C.va .......... ..TG.T..GG ..A.G..T.. .......... .........T ....AC.... ........A. ...CGATA.. CG...C...A ..........

L.ga .....C.... ..T.AT..AT .AA.G..C.. ...T...... .......... .....A.... .......... .A.CGAAAA. .G...C.... .....C....

A.po .......... ..T.GT..C. T...A..C.. .......... .......... ....AC.... .......... .A.CA.AG.. C...CC.... ..........

B.pa .....C.... ..T.GG..CA A...GG.C.. .G....C... .......... ....A..... ........G. A..CCGA.GT C....C.... .....C....

L.la C...C..... ..T.GT..C. GAA.A..C.. .G.T....G. .......... ...AAC.... ........G. AGG.GA.A.T GG..GG.... ..C..C....

P.oli .....C.... ..C.A...CG AAAAA..C.T ......A... .......T.. ....AC.... ........A. A.-CGACA.T A.T.CA.... .....C....

P.ste .....C.... ..C.A...C. AAA.G..C.. ......A... .......T.. ....AC.... .......T.. A.-AAACA.. A...TC.G.. .....C....

C.lu .....C.... ..TGA...C. AA..GC.CTT ......A.G. .......G.. T....C.... .......AT. G..C.AAA.. CCG.GC...A .....C....

B.my G....T..G. ..T.GTA.GT G...GGAC.T .C.A..C.C. .......T.. C..T.C.... .A......A. ..G..AACGG AGT.GA.... .....C....

A.te CCAATGTTTA GCTGTAGAGA CTTCGCAACA CAATAAAATT ATCATAAAAT AAAACCGCTT TAGCGCAGCG CCCCCTCAGC CCTAGTACAT CAACGCAACG

P.ii ....C..... ..C...AC.T ..G.A..... .CG.CG.... G..G..G..G .......... ..A..A..A. ..G....G.. ..C....... ..........

C.az ...GC..... ..C...AC.T ..A.T..C.. .C..TG.... G..G..G.C. ..G...AT.. A....T..G. ..G....G.. ..C....... T..T.A..AA

C.ko ...GC..... ..C...AC.C ..A.TA.C.. .C..TG.... G..G....C. ..G...A... A.T..TG.A. .AA.T..G.T ..C....... T..T.A..AA

C.va ...GC..... ..C....C.C ..A.T..... .T..TG.... G..C....T. ..G...A... A....TG.AA ..A.T..G.. .......... ...T.A..AA

L.ga ....C..... ......AC.C ....TT.C.. .C..CG.... ..T......G ..G...T... G.A.C.G.G. ..G.T..G.. ..C....... ..........

A.po ....C..... ..C....C.C ..C.TT.C.G .....G.... ..A......A ..G....... G.......G. ..G....G.. ..C....... ..G..T....

B.pa ...GC....G ..C....C.C .....TTC.. ....TC.... G..G..GT.G ..C.G..... ..A.....AA .......G.. ..C....... ..C.......

L.la ....C..... ..A....G.C ....T.TC.G .GC.CG..A. ..AG.CG.C. .......... G.A.A.G.G. ..AT..AG.. ..C....T.. ..CG..T...

P.oli ....C..... ..C...ACAC ....C..C.G ....C..... G........G ....G..A.. A.A.A....A ..GT...G.. ..C....... T.C.......

P.ste ...GC..... ..C...ACAC ....C..C.G .C..T..... G.....G..G ..G.G..A.. G.A....... ..GT...G.. ..C....... T.C.......

C.lu ...C...... ..C....CA. ...A...CAG TGC.C....C C.AG.....G .T.....G.. A.T.T....A ......TG.. ...G...... ..CA......

B.my ...GA..... .......C.G ....A.TCTG .....GT... C..GC.C.GG .G..T..A.. ........GA ..A....G.. ..C...G... A.TT...C..

A.te CCACGACGGC TGCCATTTTC CCTTGAGCAA CCAACGAATT TCCACCAGAC CCGATTAGAC GCCCCACGAT G--CTAAGGG -CTCTTAACA GAGACTCGTG

P.ii .AC.A..... ...T.C...T ....T....G .....C..A. ..T.A.CA.. .A..C...G. ........GG .--.C.C..A A......... ..........

C.az .AC.A.A... .....C.... .......... ...G....G. ..TTT.CCGA ...G...... ....AC..GG .CCTC.C..A G..T...... ..........

C.ko .AC.A..... .....C.... .......... ...GTA.... ..TTT.CCG. ...C...A.. .....T..GA --TA..C..A G..T...... ..........

C.va .A.AA.A... A....C.... .T....A... .......... ..TTTTCTGT ...C..G... ..T.A...GA .--.C.C... T..T...... ..........

L.ga ..G...A... .....C.... .........G ....A.G.CG ..TCT.CC.. ...C.A.... .....G..G. .--.CGC..- A......... ..........

A.po ..G....... .....C.... .........G ....A.T.C. ..TTT.CC.. ...G.A..G. .......... A--T..C... A..T...... ..........

B.pa .TG....... .....C..G. ...C.T.... .....A..G. ..TTTTCCT. .G.T.AG.G. A.......GG .--AC.C..A A......... ..........

L.la ..G...GT.. .....C.... .GA....... .....AG.GC ...C..CAGT GA...AGCT. .....G.AGC .--T.GC..A A..T...... ..........

P.oli .G........ A......... ......A... G....AG.AC ..TTT.CC.. .....A.AGA C....G...C .--......A A..T...... ..........

P.ste ..G...G..T A..T.C.... ......A... T.....GGGC ..TTT.CT.. ...G.AG..A C.T..G..GG .--.C....A A..T...... ..........

C.lu .GGA.G...T ...T.C.... .....G.... ....ATG.CG ...T..TAC. ...T.GGCT. A........A .--.C....A A......... ......T...

B.my ..GG.....A .T...C.... .G..AG.... .....C..AC C.TCA.CATT ...C.A.AT. ..T.....CA .--.CCC..A A..T...... ..........

A.te GTGGAGGAGC ACCACGGCAC CGGCCGGGCA TAGAGAGCGT GAATTAGACA AAGGTGATCA CCAGGGCGAG TCCAAAAATT CCCCTTCTCC GTTGGGGGGG

P.ii .......... .......... .......... .......... .......... .......... .T.A...... .......... .......C.. ..........

C.az .......... .......... ...T...... .......... .......... .......... .......... .......... .......C.. ..........

C.ko .......... .......... ...T...... .......... .......... .......... .......... .......... .T.....C.. ..........

C.va .......... .......... ...T...... .......... .......... .......... .......... .......... .......C.. ..........

L.ga .......... .......... ....T..... .......... .......... .......... .......... .......... .......CT. ..........

A.po .......... .......... ....T..... .......... .......... .......... .......... .......... .......C.T ..........

B.pa .......... .......... .......... .......... .......... .......... .......... .......... .......C.. ..........

L.la .......... .......T.. .......... .......... .......... .......... .......... .......... ......TC.. ..........

P.oli .......... .......... .......... .......... .......... .......... ...A...... .......... .......C.. ..........

P.ste .......... .......... .......... .......... .......... .......... .T.A...... .......... .......C.. ..........

C.lu .......... .......... .......... .......... .......... .........G .......... .......... .......C.. T.........

B.my .......... .A......G. ..C..T.... .......... .......... T........G .T........ .......... .......C.A ..........

A.te AGTAGTCCTG GACGCGGGTG GCAATTCCCG GATTACGGAA TAAAATAAAC GGATATCGCT TGTGGCAAGG CCCCTCCGCG GGAAACCAGC ATAAATTGCG

P.ii ........C. .......... .......... .......... .......... A......A.C .......... .......... ......T... ..........

C.az ........C. .......... ........T. .......... .....A.... .......... .......... .......... .......... .C........

C.ko ........C. .......... .......... .......... .....A.... .........C .......... .......... .......... .C.....C..

C.va ........C. .......... .......... .......... .....G.... .........C .......... .......... .......... .C.......A

L.ga ........C. .......... .T........ .......... .......... .......... .....T.... .......... ......T... .C........

A.po ........C. .......... .T........ .......... .......... A..A.....C .......... ...T...... .......... .C........

B.pa ........C. .......... .T........ .......... .....C.... C..A.....C .......... .......... ......T... .C........

L.la ........C. ..T....... .T........ .G........ .....A.... A..A...A.C .......... .......... .......... .C........

P.oli ........C. A......... .......... .......... .....A.... AAGA...A.C .......... ........T. .......... .C........

P.ste ........C. .......... .......... .......... .....A.... A.GA...A.C .......... ........T. .......... .C........

C.lu ........C. .......... .T........ .G........ .....A.... .C.A...A.C .......... .......... .......... .C..G.....

B.my ........CT .......... ..G....... .....T.... .....G.... CA.G...C.C ...T.TG..A .......... .......... ..........

A.te GGGGCATTCC CTTTTGCCGG TATACCGTGA ATCAGGTTTG AAGCTGTAGA GTGAAGAGCC GTAGTGCCAT AGGAGGGACG TTATGAAAAG ACAGGAGTAT

P.ii .....GC... T......... ..C....... ........G. .......... ........T. .......... .......... .......... ..........

C.az ......C... T......... ..C....... ........G. .......... .......... .......... .......... .......... ..........

C.ko ......C... T......... ..C....... ........G. .......... .......... .......... .......... .......... ..........

C.va ......C... T......... ..C....... ........G. .......... .........T .......... .......... .......... ..........

L.ga ......C... T......... ..C....... .......... .......... .......... .......... .......... .......... ..........

A.po ......C... .......... ..C....... .......... .......... .......... .......... .......... .......... ..........

B.pa ......C... .......... .......... .......... .......... .......... .......... .......... .......... ..........

L.la .......... T......... ..C....... ........G. .......... .......... .......... .......... .......... ..........

P.oli ......C... .......... .......... .......... .......... .........T .......... .......... .......... ..........

P.ste ......C... .......... .......... ........G. .......... .......... .......... .......... .......... ..........

C.lu ......C... .......... ..C....... ........G. .......... .........T .......... .......... .......... ..........

B.my ......C... .......... ..C....... .......... .......C.. ....G...G. .......... .......... ...G...... G...A.....

A.te CGAACGGGAA TGACACTGCG TATCTAGGGC AGAGTGATTC GAGCCGATTG GGCTCTGCTA GGGTGAGGGT GCTTCCTAGT ACCTTTTAGC TGGATGGGAA

P.ii ...C...... .......... .......... ....C..... .......... .......... .......... .......... ...C..A.A. .........T

C.az ...C...... .....T.... ...T...... .......... .......... .......... .......... .....T.... ....G.A.A. .........C

C.ko ...C...... .......... ...T...... ....C..... .......... .......... .......... .......... ......A.A. .........C

C.va ...C...... .......... ...T...... ....C..... .......... .......T.. .......... .......... ....G.A.A. .........C

L.ga T..C...... .......... .......... ....C..... ..A....... .......T.. .......... .......... ....G.A.A. .........C

A.po ...C...... .....T.... .......... ....C..... .......... .......... .......... .......... ....G.A.A. .........C

B.pa ...C...... .......... .......... ....C..... .......... .......... .......... .......... ....G.A.A. .........C

L.la T..C...... .....T.... .......... ....C..... .......... .......... .......... .......... .T.CG.A.A. .........C

P.oli T..C...A.. ....C..... .......... ....C..... .......... .......... .......... .......... ....A.A... .....A...T

P.ste ...C...A.. ....C..... .......... .......... .......... .......... .......... .......... ....A.A... ..C......T

C.lu ...C...A.. .......... .......... ..G.CT.... .......... .......... .......... .......T.. ...C..A... .....A...C

B.my T..C...C.. .......... .......... ...AC..... .......... .......... .........C C......... ..AC.CA... ..C......C

A.te ATTCCCTCGC GGACCCTTGT CGGTACTAAG TTGGTCGTCG GGAATCTAAT GGTTGACGGC TGCCAGAAAG TCCGTCCCTC ATGGCGTGCG C--CA-----

P.ii .......... .......... .......... ..A....... .......... ...A.....T ..G..A.... .......... .........A .TG.G-----

C.az .......... .......... .........A ..A....... .......... ...A...... ..G.GA.... .......... .........A .TAA.CTCTC

C.ko .......... .......... .........A ..A..T.... .......... ...A...... ..G.GA.... .......... .........A .TAA------

C.va .......... .......... .........A ..A....... .......... ...A...... ..GTG..... .......... .........A .TGA------

L.ga .......... .......... .......... ..A....... .....T.... ...A...... ..G..A.... .......... .........A .TAA------

A.po .......... .......... .......... ..A......A ..G....... ...A...... ..G..A.... .......... .......... .CGA------

B.pa .......... .......... .......... ..A......A ..G..T.... ...A...... ..G..A.... .......... .........A .CGA------

L.la .......... .......... .......... ..T......A ........G. ...A...... ..G..A.... .......... .........A .CGGGCA---

P.oli .......... .......... ....G..... ..A...A... .......... .......... ...A....G. .......... .......... .TA-------

P.ste .......... .......... .......... ..A...A... ...T.T.... ...A.....G G.G..T.... ..T....... .......... .AAT.G----

C.lu .......... .......... ........G. ..A....... .......... ...A...... ..GT.AT... .......... .........A .CC.GCGA--

B.my .......... .......G.. ........G. ..C....... ..G.C..... ...A.....G G...G.C... .......... .......... AAAG------

A.te ---------- AGCCTCCGCC GGGTCCAGGC TCTCGCGTAA GTCAAAAGCT AAAGAGAGAT AGACACGTCG AGGATAGCCG AGTAAAGACT CCCCTAAGGG

P.ii ---------- .......... .......... C......... .CT.....T. ..G....... .....T.... .......... ....T..C.. ..T.......

C.az ATGTACGTTC ......A.T. .......... C......... .C......T. ..G....... ....G..... .......... G.C.T..T.. ..........

C.ko ---------- -.....A.T. .....T.... C......... .C......T. ..G....... ...TG..... .......... G.C.T..C.. ..........

C.va ---------- -.....A.T. .....T.... C......... ........T. ..G....... ....G..... .......T.. G...T..C.. ..........

L.ga ---------- -...G..... .........T C......C.G .A........ ..G......C ....G..... .......... ....C..C.. ..........

A.po ---------- -...A..... .......... C......... .AT....... .........C ....G..... .......... G...C..C.. ..........

B.pa ---------- -...A..... .....T.... C......C.. .A........ ..C......C ....G..... .......... G.C.C..C.. ..........

L.la ---------- ....A..... .......... C......C.. .A......T. .CG......G .......... .......... G.C.C..C.. ..........

P.oli ---------- ........T. .......... C......... .A........ ..G......C ....G..... ..A....... .AC.C..C.. ..AT.C...A

P.ste ---------- ........T. .......... C......... .A........ ..G.....CC ....GT.... .......... .AC.C..C.. ..AT.T...A

C.lu ---------- G...G..... .....T...T C....TAC.G AAT.....G. ...A.....G .A.TG..... ..AG...... G.CG...C.. ..........

B.my ---------- ..T.A...T. .....A...T CA...T.... ..G..G.... ..TCG..AG. ....G....C ..AG..A... T.CGGG.... .....T...A

A.te AGCCAAGAGC GCCTTTATGT AGTGGCGTGG TACGATGTAC GCTCCTGGGC CCGGCGAACA CACGAGGGGC CGTGCCGCGG AGGGACGCCA CAGTAGGCCG

P.ii .........G .......... ...C...... ...C...... .....C.... .G.....T.. ...A...... .T........ .......... ..........

C.az .........G .......... .....T.... ...C...CG. .....C.... .G...T.T.. .....T.... .T........ .......... ..A.......

C.ko .........G .......... .......... ...C...CG. .....C.... .G...T.T.. .....T.... .T........ .......... ..A.......

C.va .........G .......... .......... ...C...... .....C.... .G...T.T.G .....T.... .T........ .......... ..A.......

L.ga .A..G....G .......... .......... ...C...... .....C.... .A....GT.G .......... .T........ ....G...T. ..A.......

A.po ....G....A .......... .......... ...C...... .....C.... ......GT.. .......... .T.....T.. .......... ..A..A....

B.pa ....G....A .......... .......... ..AC.C.CG. .....C.... .A....GT.G .G........ .T........ ....G..... .GA..C....

L.la .T..G.A..A .......... .......... ...C.C.CG. .....C.A.. .G....GT.G .......... .T.AG....A ....G...T. ..A.......

P.oli ....C.A..A .......... .........T ..AC.C.C.. .....C.... .A.A..GT.. .G.AT....T .T..A..T.. ....G..... ..A..AA...

P.ste ....C.A..T .......... ...C.....T ..AC.C.C.. .......... .A.A..GT.. .G.AT....T .T..G.T... ....G...T. .GA...A...

C.lu .A.......A .......... .......... ..AC.C.... .....C.... .G...TCT.G .G........ .T........ ....G...T. ..T.GAC...

B.my .........G .......... .....A.... ..GC...CG. .A.....A.A .A.A.A.GAG .....T...A .T.....T.. ....G..... ..A...C...

A.te GTTGCTTGAT GGACATACAG TGTATCGCTG CTTGGATGGA ACCCACGCTT AATCTTTCAT TAAACTAGCG CCCCACCAAC AACAACAAAT TTCTTAACAC

P.ii .......... .........A G...G.AT.. .........G .......... .......T.. C..G.A...A .GG....... G...GG..G. A........T

C.az .......... .......... G....T.T.. A..A.....C .......... ..C.....G. ..GC.G...A .TG....CGG C.T.G..T.. A........T

C.ko .......... .......... G....T.T.. A..A.....C .......... ..CT...T.. ..GC.G..TA .TG....CGG C.T.G..T.. A........T

C.va .......... .......... G......T.. T..AACCC.T .......... ..C.....G. ...C.G.ATA .TG....CGG C.T.G..T.. A......T.T

L.ga .......... .......... G...CT.... G..AC.GC.G .......... ..CT...... ...G.A..T. .AA..G.GG. GC.GG..... A...C....T

A.po .......... .......... G...C..... ....C.G..G ...T...... C.C....... C.GG.A..T. TTA..G.GG. G..G...... A...C....T

B.pa .......... .......... G...CT.... G..AC.G..G .......... T.CG...... C....G..TA .AA..G.CG. GG.CC..... A......T.T

L.la .......... .........A G...C.A... T..ACTA..G ...T..A... ..CA...TG. CT.G.A..T. .AA..G.C.. GTG.C..G.. A........T

P.oli .......... .......... G.GGC.C... A..TT.A..G .......... ..CG...AG. .....C..T. .AT..G..C. ....CA.GT. A........T

P.ste .......... ........G. G.GGC..... A..TC.A..G .......... G..G...A.C .....C..T. .AT..G..T. ....CA.GC. A........T

C.lu A......... .......... G...C.C... G...C....G .........C ..CG...... C.GG.A..A. .AA....GCG T.T.TG.G.. A...C..G.T

B.my .......... .......... G.A.CG.... G..ATTGA.G ......T... T.CG...TG. C....A.TTA .AA..GTGGA GG.TTAGC.. ....C..T.T

A.te TGCTGTCATC GACCAAAGCA CCTCCTCACA GCTTAACATG GCTTTCGCTT ACCCACAGAG GCATGGCTGT CAATCAAGAT CGCCCTATAC AACCTCACGC

P.ii .......... ...T.GC.AG ....T..G.. A..GG..CCA A.A...A..A ....C..ATC .......... T.G.TC.A.A .......... .......A..

C.az .......T.T .....GC.AG .......T.. A..AC..... A.A..TA..A G...C...C. .......... ....TC.A.A T......... .....T.A..

C.ko .......T.. .....GT.AG .......T.. ...CG..CC. A.A...A.GA G.TT.G..C. .......... ..G.TC.A.A T..T...... .......A..

C.va .......T.A .....GT.AG ....T..T.. A..AC..C.. A.A..AA..A G...C...C. ........A. TTG.TC.A.A .......... .......A..

L.ga .......T.. .....GC.AG .........G ...GG..CC. ..A...A..A ....C..ACC .......... ..G.TC.A.A .......... .....T.A..

A.po .......... .G.T.GC.AG .......G.. ...G...CC. C.A...A..A ....C..A.C ......T... ..G..C.A.A .......... .......A.T

B.pa .........T .....GC.AG ...G...G.. ...GG..CC. C.A...A..A ....C..A.C .......... T....C.A.A .......... ...T.T.A..

L.la .......G.. .G...GC.AT .......... A..A...C.A C.A...A..A ....C..A.C ......T... T.C..C.A.A ...T...... .....A.A..

P.oli ....A..G.. .....GC.A. T..GT....T A..A...CCA C.G..AA..A T...C.CAC. .....A.... .....TTA.A .......... .....T.A..

P.ste ....A..G.. .....GC.A. ...A.....G T......CAT C.G..AAG.A T...C.CAC. .....C.... ..C...TA.A .......... ..........

C.lu .......T.. .....GC.AG .......G.. ...GG..CG. C.A...AG.A ...TC..A.C .........G TC.....A.G .......... .......A..

B.my ....C..G.. .....GC.AG ...G....T. ...AG..CCA ..A..TA..A ...TT..A.C .........C ..G.TT.A.G T......... .....T.A..

A.te GTCTTCGAGA GGATACCACG CGCCCCGGAC ACCACGCAAA GAAACTACCT GCGGCCAGAC AGGCCCACCG GTGGTCCATA ACAGGGCAAC GCTCCAACGA

P.ii .......... .......... .......... G......... .......... .......... .........A .A...G.CCC .....A...G .T....C..G

C.az .......... .......... .......... G......... .........C .........T .....T..TA .A...G.CCC ......T..A ......CT.G

C.ko ...C...... ...G.A..A. .......... .......... .........C .T........ .....T..TA .A...G.CCC .....C...A ......C..G

C.va .......... .......... .......... GG........ .........C .T...T...T ....TT...A .A...G.CCT ......T..A ......C..G

L.ga .C.C.T.... A..C...... .......... ........G. .........C .......... .........A .A...G.CCC ..G..C..GG T.....C..G

A.po .C.C...... A..C...... .......... .......... .........C .......... .....T...A .A...G.CCC .....C...G C.....T..G

B.pa .G.C.T.... A..C...... .......... .......... .........C .........T .........A .A...G..CC .....C..GG C...T.CT.G

L.la .G...T.... A..C...... .......... .......... .........C .T........ .........A .A.A.G.CCC ..G..T..GG C.....CT.G

P.oli .G.C...... A..C...... T...T..... .G....T... .........C .......... .........A .A...G.CC. ...A.AT.GA C.....C..G

P.ste .G.C...... A..C....G. T......... .......... .........C .......... .........A .A...G.CCC ..G..AT..A ......C..G

C.lu .G...T...C A.CC...... T...T..... .....A.... .........G .......... .....T...A AA...G.CC. .....A.TGG ......C..G

B.my ...C...... ..TC...G.. ...G...... .......... .T...C...A .....T.... .....T...A .A...G..CT .A...C...G C..T..C...

A.te GGGCACGTGT GTCCACTCCG AGATGCGCCT CAGACACTCC AGGAGGCCGA TGAGATTCTC TGTTAACGAA CCGTAGTCTT CGAGCGGATC GCCACCGCAG

P.ii ...A...... .C.T...... ......A... .......... ......T.A. ..C....... .A.C...... ..AC.A.... .......... ..........

C.az ...A...... .C........ ......A... .......... ........A. ..C.G..... .TAC.....G A.AC.A.... .......... ..........

C.ko ...A...... .C.....T.. ......A... .......... .......TA. ..C.G..... .TAC.....G A..C...... .......... ..........

C.va ...A...... ..T....T.. ......A... .......... ...G...TA. ..C.G..... .TAC...... A..C...... .......... ..........

L.ga ...A...... .C.....T.. ...AC..... ...G...... ........A. ..C....... .AGC...... ...C.A.... .......... ..........

A.po ...A...... .C........ ...AC..... ...G...... .......TA. ..C....... .AAC..T... .....A.... .......... ..........

B.pa ...A...... .C.T...T.. ...GA..... ...G...... ...G...TA. ..T....... .AAC...... T.AC.A.... .......... ..........

L.la ...A...... .CT....... ...GC..... ...G...... ......TTA. ..T....... ..AC....CC ...A.A.... ..G....... ..........

P.oli ...A...... .C.T...... ...GC..... ...G...... ........A. ..C....... .AAC...... ..A..A.... ..G....... ..........

P.ste ...A...... .C........ ...GC..... ...G...... .......TA. ..C....... .AAC..A..G ..A....... ..G....... ..........

C.lu ...A...... .C........ ...GC..... ...G.....T ...G..TGC. ..C.G..... .A.CTT..GG ...C.A.... .......... ..........

B.my ...A...... .C.G...... ...GA..... ...G...... ........A. ..C...C... .AGC..T..C TTAC...... ..G....... ..........

A.te CCTGAATTAA TGGCTTTGTT TGTTCTACGC ACGCGGTTCC GGAACTGCTG GCCCAAGGCC GTGGAGATGC CAAAGGGCAC GACTCGCAAC TGGTTATCCG

P.ii ......C... ...T..C... .......... ......C... ..G..C.... ........T. .......... .......... .......... ..........

C.az T.....C... ...T..C... .......... .......... ..G..C.... .......... .......... .......... .......... ..........

C.ko ......C... ......C... .......... .......... ..G..C.... .......... .......... .......... .......... ..........

C.va ......C... ......C... .......... .......... ..G..C.... .......... .......... .......... .......... C.........

L.ga ......C... ......C... .......T.. .......... .....C.... .......... .......... ......A... .........G C.........

A.po ......C... ......C... .......... ...T..G... A....C.... .......... ........T. ......A... .......... ..........

B.pa ......C... ......C... .......... ......C... .......... .......... .......... ......A... .......... C.........

L.la ......C... ......C... .......... .......... .....C.... ........T. .......... ......A... .......... C.........

P.oli T.....C... .......... .....G.... ...T...... .....C.... ..T....... .......... ......A... .....T.... C.........

P.ste .......... ......C... .......... .......... .......... .......... .......... ......A... .........T C.........

C.lu ......C... ...T..C... .......... ......C... ....GCA... .......... .......... ....C.A.G. .G.......T C.........

B.my ......C... ......C... .......T.. .G....A... T....C.... ...T...... .......... .......... .......... C.........

A.te TGTCGGCTAA GGAGTGTATT GGAGTCGCCG CAGTATCGGT CACCGCTCTA TGCCTGTGGG GTTTCTGGGG TTTCTATATT TGTAACCCAG ACATAGCTGA

P.ii C......... .......... .......... .......... .G..A.C... .......... .......... .......... .......T.A ...G.AT.AG

C.az C......... .......... .......... ......T.A. .G..A.C... .......... .......... .......... .....A...A ...GG...AG

C.ko C..G...... .......... .......... ......T.A. TG..A.C... .......... .......... .......... .....T.T.. ...G.....G

C.va C..G...... .......... .......... ......T.A. .G....C... .......... .......... .......... .......... ...GG.....

L.ga C......... .......... .......... .......... T...A.C... .A........ .......... .......... ......TT.A G..G.AT..G

A.po C......... ..G....... .......... ......T... .C..A.C... .......... .......... .....G.... .....T...A G..G.A..AG

B.pa .......... ..G....... .......... .......... .G..A.C... .A........ .......... .C........ .......T.A .T..GA..CG

L.la C......... ..G....... .......... .G........ .T..A.C... .......... .......... ...T...... .......TG. .A.G.A...G

P.oli C......... ..G...G... .......... ......T... .C..A.C... GA........ .......... .C........ .......A.A .A.AGC..A.

P.ste C......... ..G...G... .......... TG..T.T... .C..A.C... .......... .......... .C........ .........A .A.AGC..AG

C.lu C......... ..G....... .......... ...A...... .C........ AC........ .......... .C...G.... .......A.A .G.GTAT.TG

B.my ........TG ..G....... .......... ...A...... .C..A.C... .A........ .......... .C...G.... ........GA GT.GGAT...

A.te CGAGTTTCCC CACGCGACTC TGTGTGCAGT GCCCTTCCTT CGGACTCCTG CGAGCCCCCC TGGCCATCAA ATTTAGGGCG TCACGCGTGT GCGGCGTGGA

P.ii .........A A......... .......... .......... .......... .......... .....CA... ..G.G...TC C..T...... ..........

C.az .......T.A A......... .......... .......... .......T.. .......... ....T...C. .CG.G....C .......... T...T.....

C.ko .........A AG........ .......... .......... .......... ....T..... ........C. .CG.G....A CT........ C.........

C.va ..C.G....A .......... .......... ......T... .......... .....T.... ........CG .CG.G..... C..T.T.... ....T.....

L.ga .........A A......... .......... ......T... T......... ......T... .....G..GC ....G..... .T........ A.........

A.po ...A.....A A......... .......... .......... T......... .....T.... .......... ..A.G..... ......A... A.........

B.pa ..C......A A......... .......... .......... .......... .....T.... ..A..G..T. ..G.GA.... C......... A.........

L.la .........A AG........ .......C.. .......... .......... .......... .....G..CC ....GT.... GT....A... T...T.....

P.oli .......... AT........ .......... .......... .A........ .......... .....CA.TC ..A.GA...T C..T..A... A.........

P.ste .......... A......... .......... .......... .A........ T......... .....G..CC ..A.GA...T C.....A... A.........

C.lu .........A A..A...T.. .......G.. ......G... T......T.. .......... ..C..GA... ..A.....TT ...T..A... C.........

B.my ..CA.....G A...T..A.G .......... ......G... T......... ........G. ..A...C.T. ..A.GA..AT .T...T.... ..........

A.te ACGCTGTTAG TTTGCAGCGC CCCCCTTGCC TTGGAACTCT AGCTCTGACC AGAATTAGCA ACCATTGTGG GGGCGCTGGA GCACGTGCCC GCACCCTACA

P.ii ..A.....G. .AC.TT.... .......... .C........ G.T.T.AC.. GT........ .......... A......... .....A.... A.........

C.az ..A.....G. .A........ .....C.... .C........ C...T.AT.A GTG...CC.. .......... A.....C... .....A.... A.........

C.ko ........G. .AC....... .......... .C........ G...T.AT.G GT....CC.. .......... A....T.... .....A.... A...T.....

C.va ..A..A..G. .A........ .....C.... .C........ G...T.AT.G GA....CC.. ..T....... A.....C... .....A.... AT..T.....

L.ga ..A.....G. .A........ .......... .......... G...T.AC.. G......... .......... A..T...... .....A.... A.......T.

A.po ..A.....G. .AC....... .......... .C........ ......AC.. .A....G..C C.T....... A......... .....A.... A.......T.

B.pa ........G. .AC....T.. .....C...T .C....T... ......AT.. .T.G...... ...G...... A.....C... .....A.... AT..T.....

L.la ..A....... .GC.T..... .....C..T. .C......T. ......AC.G .T.T.GG... .T.G...... ....A.C... .....A.... A...T.....

P.oli ..A.....T. .CC......T ..T..C.... .......... ......AC.. GT....GT.. C..G...... A.....C... .....A.... A..T......

P.ste ..A.....T. ..C.T....T ..T..C...T .C........ G.....AC.. GT....GT.. C..G...... A.....C... .....A.... A.........

C.lu ..A....... .G.....G.. .....C.... .C........ G.T...TCAT .AG...C... ...G...... .A.TA..... .....A...A A.........

B.my ..A....... .AC.TG.G.A ..A..C...T .C........ ....G..C.. .A....C... G.TG...... ......C... .....A..A. AA.ATA....

A.te ATCCATACCC AATCGCCATC TAAATGCATG AGGCACATTA ACGGACTGTC GACAGAGCCT ACCCGCTTTC CCCAGCGACA CAATGCATCC CCTAACTATT

P.ii .C...C.... ....TA...T .C..CA...T ..CG....C. ..A....A.. ..T....... .......... .......... ...A..GA.. ......C.CC

C.az .CA....... ....TAA... .T..C....T G.CA.TT.CG ..T....ACG ATTC.G.... G..A...... ....AT.... ...C...A.. ......C.AC

C.ko ..A..C.TA. ....TGG... .C..C....T G.CG.T..CG ..T....ACG ATTC.G.... T..A...... ....A..... ...C..CA.. ......C..C

C.va .CG..C.... ....TAA... .C..C....T G.CG.A..CG GTT....ACG ACTC.G.T.. ...A...... ....A..... ...G..CA.. .G....CGCC

L.ga ..A..C.... ....T..... .C..C....T ..CT...... ..T..G.A.. ATT....... .......... ....A..... ...A...A.. ......CGC.

A.po ..G..C.... ....T..... .C.GC....T G.AT...... ..T..G.A.. .C........ .......... ....AT.... ...G..GA.. ......C.CC

B.pa GCA..C.... ...TTA...T .C.GCA...T ..C...G.G. TAT..G.AGG ACT....... ...T...... ..T.AT.... ......CA.. .G.G..C.CC

L.la .CG..C.... ....TA...T .C..C....T ..C...G.C. ..T..G.A.. ACTG...T.. .....T.... ....A..... ...G..GA.. ......C.CC

P.oli ..A..C..A. .G...A.... .C...A...T .TCG..T.C. ..T..G.T.. ACT....G.. ......A..T ....AT.... ..G....A.. .A....C..C

P.ste .CA.....A. .G..CA.... .C...TT..T .TCA..T.C. ..T..G.T.. ACT....T.. ......A... ..T.AT.... ..G...CG.. .A....CG.C

C.lu GC......A. ...TTAG... .TTG.T...T ..AA.....T ..T..G.... ACT....... ...T.....T ....CA.... ..TG..CC.. ......CGAC

B.my .CG..C..A. ...TTA.... ...TG..G.C ..A.....A. .TTA.G.AGG AC.T...A.. .......... ..T.A..... ...G...C.. ....TGAGG.

A.te CCTCAAGTGG AGCACTTGAT GGACACACCA AACTGACCGC ACGGATTTTT ACAGTCCCCG GTCGCAAAAG TCTCAATCTT GACGCGAAGC ACCTGGTATG

P.ii .....T.... .......... .......... .......... .G.....C.. ..G..A.... .C..T..... A.....A..C .G........ .GT.....C.

C.az ...T.G.... .......AG. ........T. .......... .......C.. ..G..A..T. .C..A..... A...TGA... CC.....G.. ..T.......

C.ko ...T.G.... ....T...G. ........T. .......... .......... ..G..A..T. .C.AA..... A...T.A... .CA..C.G.. .AT.....C.

C.va ...T.G.... .......AG. ........T. .......... .......... ..G..A.... ...AAG.... A..TTGC... CC...C.G.. ..T.....C.

L.ga .T...T.... ..T.T..... .......... .......... .A........ ..G..A.... .C........ A...C.A... .G...C.... ..T.....C.

A.po .....C.... .......... .......... .......T.. .A.....C.. ..G..A.... .C........ A...T.A... .G....T... ..T.....C.

B.pa .....C.... .......... ........G. .......T.. .A........ ..G..A.... .C.A.G..G. A..TT.A..C C......T.. ..T.....C.

L.la .....T.... .......... ........T. .......T.. .A........ ..G..A.... .CT.....G. ...TC.C..C ..T.GC.G.. ..T..A..C.

P.oli .T.T.C.... ..A.A..... ........A. .......A.. CA.....C.. ..G....... .C.T.C.TG. A...C.C..A .CACAATT.G ..T....CC.

P.ste .T...T..A. ....A..... ........A. .......A.. CA.....C.. ..G....... ...T.C..T. A..TC.C..G .C...TTT.G ..T....C..

C.lu .T...C.... .......... ....G..... .......... .A.....C.. ..G..A.... .....G.... A..AC.A... A.A.TA...G ..T.....C.

B.my .G...C..A. ....A..C.. ........AC .......G.. CGT.G...C. ..T....... .CT..GG.G. AT....C.CA .TGATA.CA. ..T.C.G.C.

A.te TCGA-ACCTG ACCTCCAGCG GGCAGGTAGC GGGTCACGGT GAACACCGCA CTAAGCGTCT AACGCTGAAA AATATCCCAG CATCAGTTTG ACAGTGAGGA

P.ii .T..-..... .......... .......... ...C...... .......A.G .C..C.T... .......G.. .......... T......... ....C.....

C.az .T..-..... ....T..... .......... ...C...... .......A.G .C.CC.A... .......... GG........ ...T...... ....C.G...

C.ko ....-..... .......... .......... ...C...... .......A.. .C.CC.A... ....T..... GG........ .......... ....C.G...

C.va ....C..... .......... .......... ...C...... .......C.. .C.CC.A... .......... GG...T.... .......... ....C.G...

L.ga ....-..... G......... ...G...... ...C...... .......ATG .C....A... .G........ .G........ .......... ....C.G...

A.po .T..-..... G......... .......... ...C...... .........G .C....A... .G........ .......... .......... ....C.G...

B.pa .T..-..... G......... ...G...... ...C...... ....G..C.G .C.CCAA... .......... .......... .......... ....C.G...

L.la ....-..... G.T....... ...G...... ...C...... .....T.ATG .C........ .G.....G.. .G........ ...T...... ....C.G...

P.oli ....-..... G.T....... ........A. ...C...... .....AAA.G .C....A... ..T....GG. .G........ .......... ....C.G..G

P.ste ....-..... G......... ........A. ...C...... .....AAA.G .....TA... .GT....G.. .G........ .......... ......G...

C.lu ....-..... G......... ...G...... ...C...... ....G..A.G .C.GC.A... .C.....G.. .GG..T.... .......... ....C.....

B.my .T..-..... G......... .......... ...C...... .......T.G .A..CA.... .CA....GG. ...G....G. .......... ....C.....

A.te TACACTTGGG GAGCAAGCGC ATTGCTTCGA AATGCACACA CCTGCGCCCC CCCAGATTTA AACGACGCCC CCACGGGCAA AATTTGTGCT AAAAAGCGAC

P.ii C.....GC.. ..A....... .......... .......... AAC....... .......... G......... ....A..... .....A.T.. ...C..G...

C.az CG....GCA. ..A....... ....T..... .......... AAC......A ....TG...G G.T....T.. .......... .......... ..TC......

C.ko C.....GCA. ..A....... .......T.. .......... AAC......G ..T.TG...G G......... T......... .G.C...... ...T......

C.va .G....GCA. .GA....... .......T.. .......... AA........ ..T.TG.... G.T....... T......... ...C...... ..GC.....T

L.ga C.....GCA. ..A....... .......... .......... A.......A. .......... G......... ....A..... ...A.A..T. ...C......

A.po C.....GC.. .......... .......T.. .......... A.......GG .......... .......... ....A..... ...C.A.A.. ..CC......

B.pa C.....GC.. ..AT...... .......... .......... AGC......G .........T G......... ....A..A.. ...C.T.T.. ..CT.....T

L.la C.....GT.. ...T.....T .......... .......... AG....T.AA T......... G......... ....A..... .G.C...... ..CC......

P.oli C..T..GCA. TCA....... .......T.. .......... AGC.....AG .......... G.T....... ....A..... ...C.A.TT. .CGC..A...

P.ste C..T..GCA. .CA....... .......... .......... AG......GG T......... T......T.. T...A..... ...C.A.TT. .CGT..T...

C.lu CG.T..GCA. ..A....... ...A...... .......... ..C.....GG .......... G......... ....A..... .....A.... ......T...

B.my CC....GA.. .......... ....T..... .........G AGC.....GT T.......CT GTG....... ....A..TC. .......T.. ..CT.....T

A.te AAGGTTTCAT TAACCGCACG CAAGCGCGCA CGCCCGGCCT ACCCATAGAC GCATGCAAAC GACCCAGGAT GCCAGCGCCA GCCCAAACCC CCT--TGAGC

P.ii ......CT.. .........A .......T.. .....AC..C C...CC.... .......G.. AG--..A..G AT.G..A..T T.TGG.CT.. ..CAA..CT.

C.az ......CT.. C.....G.TC ..C....T.. .........G C....CG... .T...TG... A.--A.A... AT.CA..A.C T.TG..CT.A A.GAA..CA.

C.ko ......CT.. ......G.T. .GG....T.. .........G T....CG... ......G... A.--A.A..A A..CAT.A.C T..G..CT.A .AAAA..CA.

C.va G......T.. C.....G.T. GGC....T.. .........G C....CG... .T........ ..--A.A... AT.CA..G.T T.TG...TAG .TCAA..TA.

L.ga ......CT.. C......... .GC..C.T.. .....A...C C....C.... .....T.C.. A.--.T.... ...CAT...C T..GG.C..A ..CCG.....

A.po ......CT.. C......G.. ..C....T.. .....AA..A C..T.C.... .......T.T AC--TTA... ...T.T...T T..G..C..A ..CCA...A.

B.pa ......CT.. C.....G..T .C.....T.T .....AA..C G..TC..C.. ......GG.T A.--GCA... .....T...G T..G..C..A ..CCA.CGT.

L.la .......T.. C......T.. .GG....T.. .....A.... C..AC..... .....TG..T A.--GCAT.. ..GC.TCTTC T....GC..T ..GCA...T.

P.oli ......CT.. C......C.A ..C....T.. .....A.T.C C..ACCGC.. .....T.G.T C.--ATAAT. A.G..TA... T..G.TC..T ..ACC...T.

P.ste .....ACT.. .......C.A .G.....T.. .....A...C ...GCCGC.. .....T.G.. C.--TCAAT. ATG.AT.T.G T..G.....A ..GCC...T.

C.lu ......CT.. C.......TT .GC....T.. ....TAA..C G...CC.T.. .T........ A.--ATA... .TTTATA..C T.GG..CG.A .AGCAG.CT.

B.my ......CA.. CG....GG.. .CGA.T.T.. ...T.AC..C G...CC.C.. ......GT.T TG--G.A.T. .TG..T.T.G T.TATCTT.G ..ATGG.TC.

A.te GAAGGAAGTT AACACCTCCT GCGAGGGGAC TATAAAGATG AAAATATGTT TTATACAGTT GATGATGTGT TTACGGCTAA CTTATCCATT ATAAACGAGA

P.ii .......... C..C.....C ......AA.. .......... G......... .C........ .....A.... ....T..... .........C .G.C......

C.az ......CT.. C..C..GA.C A....CA..A .......... ........C. .C..G.G.C. .......... ....T..A.. .......... .G.CG.....

C.ko ......GT.. C..C..AA.. A....C.A.A .......... ........G. .C..G.G.C. .......... ....T..C.. .......G.. .G.CG.....

C.va ......GT.. C..T..GA.C A....CA..A .......... C.......A. ....G.G.C. .....C.... ...TT..C.. .......... .G.CG.....

L.ga .......... ...T.T.ATC ...G.CAA.. .......... ..C....... .CG.....C. .T.T.C.... ....T..... ......TG.C .G.CG.....

A.po .......... G..T....T. AG.G...A.. ......T... ..C....... .C..G...C. .T.T.C.... ....T..C.. .....T...C .G.C.T....

B.pa .......... G..C....TC .A.G...A.G C......... ..C....... .C......C. .T.T.C.... .......CGG .......... .G.CG.....

L.la ......G... G..C...T.C A..G.CAA.G ......A... .......... .C......C. .T.T.C.... ....T..C.. .........C .G.C......

P.oli .......... G..C.TG.TC CG.T.AAA.A ......AT.A ...GC.C... .A..G...C. ...T.C.... .......C.. .....T...C .G........

P.ste .......... G..T..G.TC AG.G.AA..A ......A... ....C..... .A......C. ...T.C.... .......CG. .........C .G........

C.lu A.....C... G......TTC A....CA.TA CC.GG..... .......... .C..G...C. .T.T.C.... ...AT..C.G .........C .G.TG.....

B.my ......T... ...T...TTC ...GC.A..G ...TC..C.. TGG....... .GG.....C. ...T.C..AC .......AG. .........C .G........

A.te AATCCTAGTG GGGACTTTCA GTTCGCTGGA AGGCCGGCTA CGGGAGCATG AGTCGAATAT TGCCCAACTA AA-GAACCCG GAGGGGAGAT GCTGCCCTCC

P.ii .......... .......C.. ......G... .......G.. .......... ...A...C.. .......GA. ..C.C.G..C .CAA...... ..........

C.az .......... ....T..C.. ......G... .......... .......... ...A...CG. ..T...GGAT .GA.C.A... .GAA...... ..........

C.ko .......... .......C.. ......G... .......... .......... ...AA..GG. ......GTAT GGA.T..... .GAC...... ........T.

C.va .......... .......C.. ......G... .......... ......T.C. ...AA..CG. ......GGAT ..A.C.A... .GA....... ..........

L.ga .......... .......C.. ......G... .......... .........A ...A...C.. .....G.TGG .GC.T.T..A .GAC...... ..........

A.po .......... .......C.. ......G... .......... .......... ...A...C.. .......GA. .GTAC....A .GAA...... ..........

B.pa .......... ....T..C.. ......G... .......... .A.......A .......C.. ..T...TGAG CGA...T... .GAA...... ..........

L.la .......... ....T..C.. ......G... .......T.. .A.......A ...A..C.G. ..A....TA. CCG...T..A .GA....... ..........

P.oli .......... ....A..C.. ...T..G... G......A.. .......... ...A.C.C.. ..TG.CTGGT ..A.CTT..C .CA....... ..........

P.ste .......... ....A..C.. ...T..G... .......... .......... ...A...C.. ..A...TGAT .GA.T.T..C .CA....... ..........

C.lu .......... .......... ......G... .......T.. .........A ...A...CGA ..A.....GT .GC.C.T..A .G.C...... ..........

B.my ......G... .......CT. ...GT.G... .......A.. ....G.T... ...TA..C.. ..T...TGGG .GA.C.T..A .CAC...... ..........

A.te TGAGGCACGT GCCCTAAAGG GGATCCGCGA CACGCACAAC AGTTTCGGCA AGTAGATGCA CAGGAAAGGT TATACCGCAA GGAGCACCCT GTCCATACGT

P.ii .......... .T........ .......... .C......G. .A.C.T.... .T........ ...A...... .......... .A........ ..........

C.az .......... .......... ......T.A. .C.....TG. .T.C.T..T. .C........ ...A...... .......... .A........ ..........

C.ko .......... .......... ......C.AT .C.....TG. .T.C....T. .C........ ...A...... .......... .A........ ..........

C.va .......... .......... ....T.C.A. .C.....TG. .T.C.T.... .T........ ...A...A.. .......... .A..T..... ..........

L.ga .......... .......... ........A. .C.....CG. .A.C...... .......... ...A...... .......... .A.......C ..........

A.po .......... .......... ......A.A. .C......G. .A.C...... .C........ ...A...... .......... .A.......C ..........

B.pa .......... .......... ........A. .CAAG...G. .A.C.T..G. .C........ ...A...... .......... .AG..G...C ..........

L.la .......... .......... ........A. .C......GT .A........ .C........ ...A...... .......... .A.......C ..........

P.oli G......... .......... ........A. .C..A...G. .A.C...... .T........ ...A...A.. .....T.... .A.......C ..........

P.ste .......... .......... ........A. .C..A...G. .A.C...... .C........ ...A...... .....T.... .A.......C ..........

C.lu .......... .......... ........AG .C.....CG. .A.C...... .....C.... ...A...A.. .......... .........C ..........

B.my G......... .......... ......A... .CACG.A.G. .A.C...... .T...C.... ...A...... .......T.. ....A....C ..T.......

A.te TAGACTATTG GAACACAGGC GACAAGGACA CACCATTTTA AGACGCAGAC TCGGTTTAGG AAGGCAATCT AGTGTGCACC GATCAGGTAC CGGTTGCAGT

P.ii ......C... T......... .......... .G......C. .......... .T........ ........G. ....C....T ...T...... .........C

C.az ......C... T......... .........C TG........ ..T..T.... .......... ........GC ........TT .......... .........C

C.ko ......C... T......... .........C TG.G....C. ..T..T.... .......... .........C ....C..... .......... .........C

C.va ......T... T......... .........C TG.G....C. ..T....... .......... ........G. ....C.T..T ...T...... .........C

L.ga ......C... T......... .........C .G.T...... .......... .......... ........GC ....CA...G ..GT...... ..A......C

A.po ....T.T... T......... .........C .G......C. .......... .......... .........C ....C....A ...T...... ..A......C

B.pa ......C... TG........ .........C ...T..A.C. .......... .......... .........C ....C....G ..G....... .........C

L.la ......C... T......... .........C TG.T....C. .......... .......... ....T...GC .........G ..GT...... .A.......C

P.oli ......T... T......... .........C ...T...... .......... .......... ....A....C ....C..... ...T.....T .C....T..C

P.ste ......C... T......... .........C .G.T....C. .......... .T........ ....T....C ....CA.... ...T..A..T .........C

C.lu ......C... TG....GA.. ......A..C T....G..C. ......G... .T........ ........GC ....CA..TT ...T.....T .CA...G...

B.my ......C... T......... .......T.T .G.G..G.AG ......G... .......... ....T....G ....C....T .........G .C........

A.te CCTTACTCAA GAGCGCGACC ACCGTGAATG GCAAATAAAC GAAGGATACA CCAAGGCAGA GAGCAAGCGC GTCAGCCAAA ACCCAACATT AACGTTCATA

P.ii ....C..... ..A....... ........A. ..T....... ...CA....C AT.C...C.. AG..G..... .....A.G.. ..A.CC.... ........CG

C.az ...GTT.... .....G.... ........A. ..C..G..GT A..C...... G..C...... A...G..... ....A..G.. ..A.GT.... ..T.....CG

C.ko ...GT..... ......A... ..T.....A. ..G..G..CT A..C.....C G......G.. A....C.... ....T..G.. ..A.CC.... ........CG

C.va ...CTTG... .........T ..T.....A. ..G......T A..CA....C G..G...... A...GG.... .....G.G.. ..A.TC.... ..T.....CG

L.ga ...A...... ....T.A..A ........C. ..C..C.... ...C.....T G..T...G.. AG..G..T.. ..T.AA.G.G .....C.... ........A.

A.po ...A.T.... ...TA.A..A ..T.....C. .....C.... ...C.....T ...C...... C......... ....AA.C.. G.T.TC.... .......G..

B.pa ...A.T.... ....TGAC.A ........A. ..C..C.... A..C.....T G......G.. AC..CG.... ..T.AA...G ..G.GC.... .......GCG

L.la ...AC..... ..AA.GA..A ........G. ..C......T ...C.....C GT.T...T.. AC......C. ..T.AA.G.G ..A.GC.... ........CG

P.oli ...ACAA... ..A...A..A ........A. ..C.T...T. T..C..A..C G..C...G.. AT..CG.A.A .GAGAA.TCC G.T..C.C.. C......CAC

P.ste ...AC..... ..A..GA... ........A. ..C....... C..C.....C ...C...G.. AC..CG.A.. .AT.AA.T.T ..AT.G.... .......GAG

C.lu ...C...... ..AGA.A... ........G. ..T..C..G. A..CA....C A..C...T.. AC..G..... ....AA.C.G ..A.GCT... ........AG

B.my ...G.A.... ..A.AGA..A ........A. ..T.....GA A..C..A..C T..T...G.. AC..CG..C. ..A..G...T GTAA.C...G GC......CG

A.te CCCGCAAGCG CGCGAGGCGC GCGTTGAGGC AAAGTTAAGC GTAAAAACCG AAAATCACTC C-TCCAGCTG T------AAA ACCATCCCCA AGAGGCGGCC

P.ii .....G.A.A ...A..A.AT ..A.A..... .G.TG...CA .AGG...... .G...T...T .GC....TAA C------T.. .......... ..........

C.az ..TA..G... G...G.A... ..A.C..T.. ..GAG...C. ...G...... .G........ TTC.AGAAAA C------C.. .......... ..........

C.ko ..TA...... G...G.A... ..A....T.. .G.A....CT ..GG..G... .G.....A.. .TC.G.AAGA C------... .......... ..........

C.va ...A.....A A..AC...A. ..A.C..T.. .GGA....AT ..GG...... .G........ TT..AGAAGA C------C.. .......... ..........

L.ga ...A.GC..A ...A..A..T ..A.C..... .G......CA .......... .G.G...... AT..A.CTCA C------C.. .......... ..........

A.po ......GA.. ...AC.A.CG ..A.CCG... .GG.G...C. ......G.A. G.....TA.T TTC.AGC.AA CTAGACTC.. .......... ..........

B.pa ......GA.. ...A..A.CG ..A.C..A.. .GGTGC..C. ..G...C... G......T.. .TC.G.TTGA C------C.G .......... ........T.

L.la ..TA....TA ...T....AG ..A.C..A.. .G.TGC..C. .GG....... GG...T.T.. .TC.G.C.C. A-------.. .T......T. ...A......

P.oli ......C..C ...T..A.AG ..A.C..A.. .G.T....C. ..G.....A. ......CT.G .TCG..A.AT A------..G ........T. .....T..T.

P.ste ...A..T... ......A.AG ..A.C..... .G.....CC. ..T....... .......... TACG..A.AC A------C.G .......... .....T....

C.lu ..A...G..A ...AG.A.C. ..A.C..... .GGTGA..C. T.....C... G.....GA.. .TG.G.TTGA .T----C..G ....A..... G.........

B.my ..G.TTT..A ...A....AG .A..C..A.. .GGTGC..CG ..TG..C... G..G.TGT.T GTG.G.CTAA A------T.G .A..A..G.. G.....A...

A.te GCTAATGTTA TGTCCGCTCA GCACAGTTCG ACTATGAGAG TTTGGCATCG GATGTAACAA CGAGGTTTTG TATACAGCGC TTGTTCTAGA TAGGGACCCC

P.ii .......... .......... ......C... .......... .......... .......... .........A .......... .....T.... ..A..G....

C.az .......... .......... ...G...... .......... .......... .......... .........A .......... .......... ..A..G....

C.ko .......... ....T..... ...G..C... .......... .......... .......... .........A .......... .......... ..A..GTT..

C.va .......... .......... ...G..C... .......... .......... .......... .........A .......... .......... ..A..G....

L.ga A......... ......T... ......C... .......... .......... .......... .........A .......... .......... ..A..G...T

A.po .......... .......... ......C... .......... .......... .......... .........A .......... .......... ..A..G....

B.pa .......... .......... .......... .......... .......... .......... .........A .......... .....T.... ..A..G....

L.la .......... .......... A.....C... ....GAG... .......A.. .....CC..C .........A .......... .......... ..A..G....

P.oli .......... ....T...T. A..T..C.T. .......... .A........ .....T.... .........A ...C...... .......... ..........

P.ste .......... ....T...TG A..G....T. .......... .A........ .....C.... .........A ...C...... .......... ..A..G....

C.lu ..G....... .......... ...T..C... .......AT. .G..A..A.. .C...C...C .........A ...G...... .......... ..C.......

B.my ..C.C..... ......T.TG A..G..C.T. ....C.G... .......A.. ..A..C...C .........A .........A .........G .....G....

A.te GAAAGTGGTG CCTGCATTTG GAGAAACCTG GCTGGAACGC TATGGTTGGA GACACTTGTC TCCCTGGCGG AGACCCTTCG AGTAACAGAA TGGGAGTTCC

P.ii .......... .......... .......... .......... .......... .......... .......... .......C.. .......... ..........

C.az .......... .......... ......T... .......... .......... .......... .......... .......C.. ........G. ..........

C.ko .......... .......... .......... .......... .......... .......... .......... .....T.C.. ........G. ..........

C.va .......... T......... ......T... .......... .......... .......... .......... .....T.C.. ........G. ..A.......

L.ga .......... .......... .......... ......G... .......... .......... ..T....... .......C.. ........G. A.T.......

A.po .......... .......... .......... ......G... .......... ..T....... .......... .......C.. ........G. ..........

B.pa .......... .......... .......... ......G... .......... .......... ......A... .......C.. ........G. ..........

L.la .......... T......... .......... ...A..G... .......... .......A.. ......A... .CC....C.. .......... .....AC...

P.oli .......... .......... .......... .......... .......... .......... ..T...AG.. .....A.C.. ........T. ..T...C...

P.ste .......... .......... .......T.. .....G.... .......... .......... ..T..AAG.. .......C.. ........C. ..T...C...

C.lu .......... .......... .......... .....GG..G .......... .......... .G........ ..C.TT.C.. T.....C.CG ..A..A....

B.my .......... .......... ......A... A..C.GG... ........A. ..T....A.. .AA..AA... .CG....C.. .......... ..TC.A....

A.te TTTTAGCCGT GGCACACAGC GCTTCACCGG CGATACGACC GACCCAACGT TTCTGTGACC TACAACGGGC GCCTTACGTA AGCTCCATAC CATATCCCAC

P.ii .......... .....GTGA. ......TT.. .........T .......... ........T. .......... ........C. C..C...... .GC.......

C.az ......TT.. .....G.GA. .......... .......... .......... .......... .......... ........C. C......... ..C.......

C.ko .......T.. .A...G.GA. .......... .......... .......... .......... .........T ........C. C...T..... ..C....T..

C.va .......... .......G.. .......... .......... .......... .......... .........T .......... T......... ..C.......

L.ga .......... ...C.GT... ......T... .......... .......... ........T. .......... ........C. GA.A...... .GC.......

A.po .......... .....G.GAT .......T.. .......... .......... ..T....... .......... ........C. GA.A...... ..C.......

B.pa ......A... .....G.GA. ......T... .......... .......... ........T. .......... ........C. GA.A...... ..C.......

L.la ..A....... .A.CAG.... .T........ .......... .......... .......... .......... ........CC C..A...... ..C....G..

P.oli .......... ...CAG.G.. .T........ .......... .......... .......... .......... ..T...TAC. C..A...... ..C....TT.

P.ste ..A....T.. ...CAG.GT. .......T.. .......... .......... .......... .......... ..T.....C. C......... ..C....G..

C.lu ...G..AG.. ...G.T.GCA ...A..GA.. .......... .......... .......... .........G ...C....CT C..A...... .GC.....T.

B.my ..G....T.. ..TGA..GAG .....TT... .......... ...G...... .......... .........A ..G.....CC G..CA..... .GC.....T.

A.te GCTTGCAGGG AATATAGGAC GGGCTAAAGC CGTGGTTTAT CACACGCGTC GAAAATA--- -TAACGATTC GTTACTTTTC TTTTCTCTTC TTAGATTGAT

P.ii ....A..... C.C....... .......... A...A..G.. .G.G.....G ....GCGA-- -.T.TAC... A......... .......... ..........

C.az .......... C......... ......C... ....A..G.. .GT....A.T ....GCTG-- -.T.TA.... A......... .......... ..........

C.ko .......... C......... .......... ....A..... TG....G... ....GCTG-- -.T.TAC... A......... .......... ..........

C.va .......... C.C....... ......CG.. .......G.. .GTG.AG... ....GCTG-- -.T.T..... A........G .......... ..........

L.ga ....A..... ..C....... .......... .......A.. ..TG..G..G ....G.GA-- -.CTTCC... A......... .......... ..........

A.po A...A..... .......... .......... ....A..G.. .G.G.AGA.G ....GCGA-- -..CTTC... A......... .......... ..........

B.pa ....AT.... ..C....... ......G... ....A..A.. .G.C..GA.G .....CGA-- -....AT... A......... .......... ..........

L.la ....TTG... G.C....... ......G... G...C..A.C .G....G..G ...G.CGA-- --TTTCC... A......... .......... ..........

P.oli T...T.G... ..C....... ..C..G.... G..TC...C. TGA...A... ....CGTC-- ---TTCC... A.....C..T C......... ..........

P.ste T...TTG... ..C....... ..C..G.... G..CA...C. .G...AG..A ...GCGTA-- ---TTCC... A.....C..T C......... ..........

C.lu TA....G... TGC....... ..A..GGG.. G..TT..... .T....G.A. ...CCA.--- -.T.TCT... A......... .......... ..........

B.my CA..T.G... ..C....... ......C... G......C.. TGT..CG..G ....GGGGTA G.CGTAT... A.C...C..T C......... ..........

A.te ATGAGCATTG CAGTTACTCA GTATTTAACT GCCCCCATTT TTCCTTCTCT CTCTGCCTCT CACTCATGTA TTTTTCTCGT CTACTTGCGG CCACAACTTG

P.ii .......... .......... .......... .......... .......... .......... .......A.. .......... .......... ..........

C.az .......... .......... .....C.... .......... .......... .......... .........G .......... .......... ..........

C.ko .......... .......... .....C.... .......... .C........ .......... .........G .......... .......... ..........

C.va .......... .......... .....C.... .......... .C........ .......... .......A.G .......... .......... ..........

L.ga .......... .......... .......... .......C.. .......... .......... .......... .......... .......... ..........

A.po .......... .......... .......... .......C.. .......... .......... .......A.. .......... .......... ..........

B.pa .......C.. .......... .......... .......C.. .......... .......... .......... .......... .......... ..........

L.la .......... .......... .....C.... .......C.. .C........ .......... .......A.G .......... .......... ..........

P.oli .......... .......... .......... ......C... .......... .......... ...CT..A.G .......... .......... ..........

P.ste .......... .......... .......... ......C... .......... .......... ....T..A.G .......... .......... ..........

C.lu .......... .......... .......... ..A....C.. .......... ..T....... .......A.G .......... .......... ..........

B.my .A........ ........G. .......... .......G.. ......G.G. ..T....... ....T..A.. .......... .......... ..........

A.te CTGCTCACTC AATGTGTTTT CCTTTCGCTC TATTCCCAAA CGTTTCCGCT CCTGTTCCTC ACAGCCTATC AGACATTCGT ATAACGGCTC TTTTCAACAT

P.ii .......... .......... .......G.. .......... T......... .......... .......... .......... .......... ..........

C.az .......... .......... .T........ .......... T......... .......... .......... .......... .......... ..........

C.ko .......... .......... .......... .......... T......... .......... .......... .......... .......... ..........

C.va .......... .......... .T........ .......... T......... .......... .......... .......... .......... ..........

L.ga .......... .......... .......... .........G T......... .......... .......... .......... .......... ..........

A.po .......... .......... .T........ .......... T......... .......... .......... .......... .......... ..........

B.pa .......... .......... GT.....G.. .........G .......T.. .G........ .......... .......... .......... ..........

L.la .......... .......... G......... .........G T......... G......... .......... .......... .......... ..........

P.oli .......... .......... AT.....G.. ..C.A....C T......... ....A..... .......... .......... .......... ..........

P.ste .......... .......... AT.....G.. ..C.A....C T......... ....A..... .......... .......... .......... ..........

C.lu .......... .......... GT........ .......... T......... .......... .......... .......... .......... ..........

B.my .......... .......... .T.....G.. .......... .......T.. .G........ .......... .......... .....C.... ..........

A.te TTTACTCCCT TTGCTAACCT CCTCCCATTT ATTCTCTTTT GTGCCTCGTG AAATTATTGA CTTCTCTGTT TGATCTCCCT CCTCCT-TAC ATTTTTTCCT

P.ii .......G.. ..C.C..... .T........ .......... .......... .......... .......... .......T.. ......-... ...CC..T..

C.az .......... ....C..A.. .....TT... .......... .......... .......... .......... .......T.C ......-... ...CC..T..

C.ko .......... ....C..A.. .....TC... .......... .......... .......... .......... .......T.C ......-... ...CC..T..

C.va ........T. ....CC.A.. ..C..G.... ..CT...... .......... .......... .......... .......T.C G.....-... ...CC..T..

L.ga .......... ....C.C... .TC....... ...T...... .......... .......... .......... .......T.. ......A... ...CC.CT..

A.po .......... ....CCC... .T....C... ...T...... .......... .......... T......... .......T.. ......C... T..CC..T..

B.pa .......... ....C..... .TC....... ..CT...... .....A.... .......... .......... .......TGC ......C... ...CC..TG.

L.la ....G..... ....CC..TC .T.G.....G ...T...... .......... .......... .......... .......... ......C.G. ....C.....

P.oli ........T. ....C.T... .....T...C .CCT...... .....A.... .......... A......C.. .......... .G...AT... T..CC..TG.

P.ste .......... ....C.T... .A..GT...C .CCT...... .....A.... .......... A......C.. .......T.. .G...AT... ...CC..TG.

C.lu .......... ....CC.A.C .TG....... ...T...... .......... .......... .......C.. .......T.. .....AC... ...CC..T..

B.my ...G...... ....C...GA A.CAT.T... ..C....... .....A.... .......C.. .......C.. C......TG. .T....C.G. ...GC...G.

A.te GTGCTCCTCG CAGTTCGTGT ATACTCTTCT TCAAAACGCT ACCCAATTCA CCCCCCTTTC GCCACGTCGA GATTATCACT CTCTCTCCTC TATGTCCTAC

P.ii ....CT.... .......... .......... .......... .......... .......... .......... ...C...... ......T... ..........

C.az ....CT.... .......... .......... .......... .......... .......... .......... .G.C...... ......T... ..........

C.ko ....CT.... .......... .......... .......... .......... .......... .......... .G.C.....C .......... ..........

C.va ....CT.... .......... .......... .......... .......... .......... .......... .G.C.....C ..T...T... ..........

L.ga ....CT.... .......... .......... .......... .......... .......... .......... ...C...... ......T... ..........

A.po ....CT.... .......... .......... ..G....... .......... .......... C......... .......... .C....T... ..........

B.pa ....CT.... .......... .......... .......... .......... .......... .......... ...A.C.... .C..T..... ..........

L.la ....CT.... .......... ...T...... ..G..C.... .......... .......... CT...A.... ...C...... .C..T..... ..........

P.oli ....CT.... .......... .......... .......... .......... .......... .T........ ...A...... .C..T.T... ..........

P.ste ....CT.... .......... .......... .......... .......... .......... .......... ...A...... .C..T.T... ..........

C.lu ....CT.... .......... .......... ..G.....G. .......... .......... .......... .G.G.CT... .C..T.T..G ..........

B.my ....CT.... .......... ..G....... ..GG...... ...T...... .......... C......... ...G.C.... .C.....G.. .........T

A.te GTCATTAGTA TCCGTTTCCG AATCCTCTTT ATACCACCAC TTTTGTTCCT TGGGGGTAAC ATGATTCACC TCATGGTTTT TATCCCTCTT CTTTATCTCC

P.ii .......... .......... .......... ........C. .......... .......... .......... .......C.. .......... ......T...

C.az .......... .......... .......... .....T..C. ..C....... .......... .......... .......C.. ...T...... ...C..T...

C.ko .......... .......... .......... .....T..T. ..C....... .......... .......... .......C.. ...T...... ...C..T...

C.va .......... .A........ .........C ...G.T..C. .......... .......... .......... .......C.. ...T...... ...C......

L.ga .......... .......... .......... .....C.GC. ..C....... .......... .......... .......C.C ...T...... ...C..T..T

A.po .......... .......... .......... ........CT ..C....... .......... .......... .......... ...T...... ...C..T...

B.pa .......... .......... .......... .....CT.C. .......... .......... .......... .......C.. .....G.... ......T...

L.la .......... .......... .........C .....GT.C. ..C.....T. ...T...... .......... .........C .......... ..C......T

P.oli .......... .......... .......... ...TA...-. ......C... .......... .......... .......... .....T.... ...C..T...

P.ste .......... .......... .......... ........-. .......... .......... .......... .......... .....T.G.. ...C.....T

C.lu .......... .A........ .......... .....C.GC. .....C..T. .......... .......... .......... ....GG.G.. G........T

B.my .......... ....G..... ......T..C .....GG.CT ..C...C.T. .......... .......... .......... .....A.... ......T...

A.te CTTTCTACAA CCCTGATCTC GCACCCTCCT CCTTTTCTGG TCCTCGTTCA GTTTAATCAA ATTCTCCTCC TCCTTGTATA TGTCACTCTC CCCAATTACC

P.ii ....T..... ....A..... .....T...C .......... .......... .......... ...T...... .......... .......... T.....CG..

C.az ....T..... ....A..... .........C .......... .......... ....T..... ...T...... .......... .......... T...G.CCG.

C.ko ....T..... ....A..... .....T...C .......... .......... ....T...C. ...T...... .......... .......... T...G.CCG.

C.va ....T...G. ....A..G.. .....T.... .......... .......... ....T..... ...T...... .......... .......... T......C..

L.ga ....T..T.. ....A..... .........C .......... .......... .......... ..C....... .......... .......... T.....CG..

A.po ....T..A.. ....A..... .........C .......... .......... .......... ..C....... .......... ......C... T.....CC..

B.pa ....T...C. ....AC.... .T........ .......... .......... .......... ..C....... .......... .......... T.....CC..

L.la ...AT..A.. ....A..... .....T..T. G..C...... .......... .......... G.C....... .......... ...A...... T.....GG..

P.oli ....T..T.. ....AC.... .....T.... T......... .......... .......... ..C....... .......... .......... T.....CG..

P.ste .C..T..T.. ....AC.... .......... .......... .......... .......... ..C....... .......... .......... TT.....GT.

C.lu .......... ....A..... .......... .......... .......... ....C..... G......... .......... .......... T.....CG.T

B.my ......CT.C T.A.AC.... .A...G.... .......... .......G.. ....T..GG. G.......T. .......... .....AC... T...G.CG.G

A.te CGGTGCCATA CTTCCCCCCC TCTTCTCCGT TCTT---CCT CCGGTTCCAA AATGCTATTT GCGCGTTGCC TGTTTGCATG ACGCTTGAAA TAATTTCCAC

P.ii ....C.T..C TC..TT.... .......... C...--C.TC .......... .......... .......... .......... .......... ..........

C.az ....C.A..C TC..TT.... .G....T... C...CCT.TC TT........ .......... .......... .......... .......... ..........

C.ko ....C.A..C TC..TTG... .G....T... C...--T.TC TT........ .......... .......... .......... .......... ..........

C.va ....C.G..C TC..TT.G.. .G....T... C...--C.TC TT........ .......... .......... .......... .......... ..........

L.ga ....C.T..C TC..T..... .......... ....--C.TC -......... .......... .......... .......... .......... ..........

A.po ....C.T.CC TC..T..... ........C. ....--C.TC T......... .......... .......... .......... .......... ..........

B.pa ........CC TC..TTG.G. .T........ C...--A.TC .......... .......... .......... .......... .......... ..........

L.la ....C.A..G TC.....GT. .G......A. ....--CGTC .......... .......... .......... .......... .......... ..........

P.oli ....C....C TC..T.G... CT......CC ....--C.TC T......... .......... .......... .......... .......... ..........

P.ste ....A....C TC..T..... CT......CC ....--T.TC T......... .......... .........G .......... .......... ..........

C.lu G...TT.GCC TC..T...T. ........A. C...--T.TC .......... .......... ....C..... .......... .......... ..........

B.my .C..A.A.AC TC..T..TT. .G....T.C. CT..--TATC .......... .......... ..T.C..... .........C .......... ..........

A.te TTTTTTTTTC TTTGGTGAGT TCTTTGCCAT CTCGTAATGT GTTCCCTTTT TCCCGTACGC GCGGCTACCT CGATCACGCC TATCTTCTAT CGTCCTTGCT

P.ii .......... .......... .......... .......... .......... .......... .......... .......... .......... ..........

C.az .......... .......... .......... .......... .......... .......... .......... .......... .......... ..........

C.ko .......... .......... .......... .......... .......... .......... .......... .......... .......... ..........

C.va .......... .......... .......... .......... .......... .......... .......... .......... .......... ..........

L.ga .......... .......... .......... .......... .......... .......... .......... .......... .......... ..........

A.po .......... .......... .......... .......... .......... .......... .......... .......... .......... ..........

B.pa .......... .......... .......... .......... .......... .......... .......... .......... .......... ..........

L.la .......... .......... .......... .......... .......... .......... .......... .......... .......... ..........

P.oli .......... .......... .......... .......... .......... .......... .......... .......... .......... ..........

P.ste .......... .......... .......... .......... .......... .......... .......... .......... .......... ..........

C.lu .......... .......... .......... .......... .......... .G........ .......... .......... .......... ..........

B.my .......... .......... .......... .......... .......... .......T.T .......... .......... .......... ..........

A.te ATTCCTTATA CCCTCTAACC TTTGCTTTCC TTTTTCTCTT CCGTCTTTCA GATACCTTAC TGGGACTTAA ATTGTTGACA TATTTTCGTG TTCATTCAAC

P.ii .......... ........T. .......... .......... .......... .......... .......... .......... .......... ..........

C.az .......... .......... .......... .......... .......... .......... .......... .......... .......... ..........

C.ko .......... .......... .......... .......... .......... .......... .......... .......... .......... ..........

C.va .......... .......... .......... .......... .......... .......... .......... .......... .......... ..........

L.ga .......... .......... .......... .......... .......... .......... C......... .......... .......... ..........

A.po .......... ....G..... .......... .......... .......... .......... C......... .......... .......... ..........

B.pa .......... ........T. .......... .......... .......... .......... C......... .......... .......... ..........

L.la .......... ........T. .......... .......... .......... .......... C......... .......... .......... ..........

P.oli .......... ........T. .......... .......... .......... .......... C......... .......... .......... ..........

P.ste .......... ........T. .......... .......... .......... .......... C......... .......... .......... ..........

C.lu .......... ........T. .......... .......... .......... .......... C......... .......... .......... ..........

B.my ......C... ..T...T.T. .......... .......... .......... .......... C......... .......... .......... ..........

A.te GAAACTGATG TTGCTTCTGT TGTTTGCAAT TCTGTATACG CATCCCCTTT CTCCGTATTG GTCCTAGGAT AGACCTTGCT GTTTTTCTGG TCGTTTCACC

P.ii .......... .......... .......... .......... .......... .......... .......... .......... .......... ..........

C.az .......... .......... .......... .......... .......... .......... .......... .......... .......... ..........

C.ko .......... .......... .......... .......... .......... .......... .......... .......... .......... ..........

C.va .......... .......... .......... .......... .......... .......... .......... .......... .......... ..........

L.ga .......... .......... .......... .......... .......... .......... .......... .......... .......... ..........

A.po .......... .......... .......... .......... .......... .......... .......... .......... .......... ..........

B.pa .......... .......... .......... .......... .......... .......... .......... .......... .......... ..........

L.la .......... .......... .......... .......... .......... .......... .......... .......... .......... ..........

P.oli .......... .......... .......... .......... .......... .......... .......... .......... .......... ..........

P.ste .......... .......... .......... .......... .......... .......... ........G. .......... .......... ..........

C.lu ...T...... .......... .......... .......... .......... .......... .......... .......... .......... ..........

B.my .......... .......... .......... .......... .......... .......... ........T. .......... .......... ..........

A.te TATTTAACAA TTCATAATTC TGCTTCTTCG TTAGTCTTCG ACTACCGCAT ATGTTTCGTA TCTTCAATTG TCGTCGGACA ACACACTGAC TCCTGCTTCT

P.ii .......... .......... .......... .......... .......... .......... .......... .......... .......... ..........

C.az .......... .......... .......... .......... .......... .......... .......... .......... .......... ..........

C.ko .......... .......... .......... .......... .......... .......... .......... .......... .......... ..........

C.va .......... .......... .......... .......... .......... .......... .......... .......... .......... ..........

L.ga .......... .......... .......... .......... .......... ..T....... .......... .......... .......... ..........

A.po .......... .......... .......... .......... .......... ..C....... .......... .......... .......... ..........

B.pa .......... .......... .......... .......... .......... ......T... .......... .......... .......... ..........

L.la .......... .......... .......... .......... .......... ......T... .......... .......... .........G ..........

P.oli .......... .......... .........C .......... .......... ......T... .......... .......... .......... ..........

P.ste .......... .......... .........C .......... .......... ......T... .......... .......... .......... ..........

C.lu .......... .......... .......... .......... .......... .......... .......... .......... .........G ..........

B.my .......... .......... .......... .......... ......T..C .......... .......... .......... .........T ..........

A.te TCTTTTTTTT GACTACAGAT TCTATCACAT AGTAGGCCCA ACTAACCTTA CATGT----- ---------- --CACCATGT AACCCCTTAA TTATAAACTT

P.ii .......... ....G..... ......T... .......... .......... A..AGAT--- ---------- --........ .......... ..........

C.az .......... ....G..... .....TC... .......... .......... ...A.ATGCG ACGATATCGA AT........ .......... ..........

C.ko .......... ....G..... .....TC... .......... .......... ...A.----- ---------- --........ .......... ..........

C.va .......... ....G..... .....TC... .......... .......... ...A.----- ---------- --........ .......... ..........

L.ga .......... ....G..... ......T... .......... .......... A..A.----- ---------- --...T.... .......... ..........

A.po .......... ....G..... ......T... .......... .......... A..A.----- ---------- --...T.... .......... ..........

B.pa .......... ....G..... ......C... .......... .......... ...A.----- ---------- --...T.... .......... ..........

L.la .......... ....G..... ......C... .......... .......... A..CCGAC-- ---------- -T........ .......... ..........

P.oli .......... ....C..... ...T..C... .......... .........G .C.A------ ---------- -T........ .......... ..........

P.ste .......... ....C..... G.....C... .......... .......... TG.ACAG--- ---------- -T........ .......... ..........

C.lu .......... ....G..... .....GT... .......... .......... AGATCGGTC- ---------- -T...G.... .......... ..........

B.my .......... ....G..... ...G.A.A.. .........T .......... TT.AA----- ---------- -T...T.... .......... ..........

A.te TTTTTGCTTT ATTTCTTCCA TCAATTTACA ATATTGCTTC CTTTTTTCTC CTGTTATTAA TATCATCTAC TGAAGAGCAA ACAAAATATA CATTCCCATC

P.ii .......... ...C...... .......... .......... .......... .......... .GC....... .......... .......... ..........

C.az .......... .C.C...... .......... .......... .......... .......... .GC....... .......... .......... ..........

C.ko .......... ...G...... .......... .......... .......... .......... .GC....... .......... .......... ..........

C.va .......... ...G...... .......... .......... .......... .......... .GC....... .......... ....G..... ..........

L.ga ....C..... .......... .......... .......... .......... .......... .GG....... .......... .......... .........A

A.po .......... .......... .......... .......... .......... .......... .GC....... .......G.. .......... ..........

B.pa .......... .......... .......... .......... .......... .......... ..C....... .......... .......... ......A...

L.la .......... .......... .......... .......... .......... .......... ..C....... .......... .......... ......A...

P.oli .......... .......... .......... .......... .......... .......... ..A....... .......G.. .......G.. ...C..A...

P.ste .......... .......... .......... .......... .......... .......... ..A....... .......G.. .......G.. ......A...

C.lu ......T..C ...C.C.... .......... .......... .....C.... .......... ..C....... .......... ...T...... ....A.A...

B.my C......... ....T..GG. .......... G..C...... .....C.... ......A... A.A....... .......... ...T.G.... ..........

A.te CGATGTTACA AGTTTCCTCC TGTTTCCAAT TACGCTCCTG TATACTCGGT AACCTTCAGC GTTAGAGCAT GGCAAGTTCT TTACCCTAAT TAGCCTTCAA

P.ii .......... .......C.. .......... .......... .......... ......T... .......... .......... .......... A.........

C.az .......... .......C.. .......... .......... .......... ......T... .......... .......... ........C. A......T..

C.ko .......... .......C.. .......... .......... .......... ......T... .......... .......... ........C. A......T..

C.va .......... .......C.. .......... .......... .......... ......T... .......... .......... ........G. A......T..

L.ga .......... .......A.. .......... .......... .......... ......T... .......... .......... .......... AG..T.....

A.po .......... .......A.. .......... .......... .......... ......T... .......... .......... .....T.... AG........

B.pa .......... .......A.. .......... .......... .......... ......T... .......... .......... .......... AG........

L.la .......... .......A.. .......... .......... C......... ......T... .......... .......... .......... AC........

P.oli .......... ....C.TA.. .......... .......... .......... ......TG.. ...T...... .......... ....T..... A......T..

P.ste .......... ......TA.. .......... .......... .......... ......TG.. .......... .......... ....T..... A......T..

C.lu .......... .......GG. .......... .......... ..C....... ......T... .......... .......... .......... A...T..T..

B.my .......... ....C.GA.. .......... .......... .......... ...G...... .......... ..T....... .......... AT..T...G.

A.te ATCATACCCG TATTTTCGTT TTCTTCAATT CAGTCAACCC TACACCAACC GCGCGATTTG TTAATACCCG TGTCTTTTCT CTCGTTTCTC CGGGTCAGTT

P.ii C......... .T........ .......... A.C....... C.T..A...T .......... .....C.... ......C... ........C. .A........

C.az T......... .T........ ..T...G... A.CA....AT AC........ .......... .....C...A ......C... ......A.C. .A.....C..

C.ko T......... .T........ ......G... A.CA....AT AC........ .......... .........A ......C... ........C. .A.....C..

C.va C......... .T........ ..T...G... A.CA..C.AT ACT....... .A........ .....C...A ......C... ......A.C. .A.....C..

L.ga C......... .T........ .......... A.AC....T. A......... .......C.. .....C.... ......C... ......A... .....T....

A.po C......... .T........ .......... A.CC....T. A...AG.... .......C.. .....C...A ......C... ......A.C. ..........

B.pa C......... .T........ ......G... G.CC....T. A...TT.... .....C.... .....C.... ......C... ........C. .....T....

L.la C......A.. .T......C. ......G... ..CC....A. A..C...... .A........ .....C..TA ......C... G.......C. .....A....

P.oli C......... .T........ ......C... ..C....... AG..A..CA. .A........ .....T..TA ......C... T...C...C. .....A....

P.ste C......... .C........ ......C... ..CA...... AG..A..CA. .A........ .....T..TT ......C... ....C...C. TC...A....

C.lu C......... .T........ ......T..C ..TC.G..A. GT.C.....T .......C.. .....C...A ......C... ........C. .C..CT....

B.my G......... .T........ .......... G.CC...TA. A...T.C.T. .T...C.C.. ........TT A.....C... ....C.A.C. ..........

A.te CTACGCTGGT CGATTCCTCC GGAAGCCTTT CTTTTTTCTA TTGTTCACTC CCCATCTATC TCTCTGTGCT TTGTGAACCA CTGATTCAGC CCCTTCTTTT

P.ii .C.....A.. .A...T..A. C........C .......... .......... .........G .......C.. .......... .......... ..........

C.az .C.....A.. .C...T..G. C........C .......... .......... .........G .......C.. .......... .......... ..........

C.ko .C.....A.. .....T.... C........C .......... .......... .........G .......C.. ....T..... .......... ..........

C.va .C.....A.. .C...T..GA C........C .......... .......... .........G .......C.. .......... .......... ..........

L.ga G......A.. .A...T..A. C........C .......... .......... .........G .......C.. .....C.... .......... ..T.......

A.po G......A.. .A...T..A. C........C .......... .......... .........G .......C.. ....A..... .......... ..........

B.pa G......A.. G....T..A. C........C .......... .......... .........G .......C.. .....C.... .......... ..........

L.la .C...T.A.. .A...T..A. C........C .......... C......... .........G .......C.. ....A..... .......... ..........

P.oli ...G.T.A.. .C...T..AT .........C .......... .......... .........G .......C.. .......... .......... ..........

P.ste ...G.T.... .C...T..A. ......T..C ....A..... .......... .........G .......C.. .........T .......... ..T.......

C.lu .......A.. .....T..A. C........C ...CA..... .......... .......G.G ..C....... .C......A. ..C....... ..T.......

B.my .C...T.A.. .A...T..A. C.....G..C .......... .......... .........G .......C.. .....C.... ..C....... ..........

A.te TACTGTTTGC TCTGTGTCCA TCCGATTTAT TCCCCTTTCC TTCCTCCTCC TTTTTTCTTA TCTCTTACAT TTTTTGTATA AATTCAACAC AATTCCGCGC

P.ii .......... .......... .......... .....A..T. ......T..T C......... .......... .......... .....C.... ....G.....

C.az .......... .......... .......... ....GA..T. ......T..T .......... .......... .......... .....C.... ..........

C.ko .......... .......... .......... ....GA..T. ......T..T .......... .......... .....C.... .....C.... ..........

C.va .......... .......... .......... ....GA..T. ......T..T .......... .......... .......... .....C.... ..........

L.ga .......... .......... .......... .....A..T. ......T... .......... .......... .......... .......... ....A.....

A.po .......... .......... .......... .....A..T. ......T... .......... .......... .......... .......... ....A.....

B.pa .......... .......... .......... .....A..T. ......T... C......... .......... .......... .......... ....A.....

L.la .......... .......... .......... .....A..T. ......T..T G..A...... .......... .......... .....G.... ....A.....

P.oli .......... .......... .......... ........T. ...T..TC.. C......... .......... .......... .......... ....A.....

P.ste .......... .......... .......... ........T. ...T..T... C......... .......... .......... .......... ....A.....

C.lu .......... .......... .......... .G...G..T. ......T... C......... .......... .......... .....C.... ....A.....

B.my .......... .......... .......... .G......T. C.....T..T G......... .......... .......... .....G.... ....A.....

A.te TCGCTCCTTT CCGCCTGTAT ACTCTTCTGT TTTTTCTGAG GGATTGAGCT AGAACCCTAA GTGAGTTTTT CCATTTTTGT TGCTAACGTC CCCATGGCGC

P.ii ....C..... ...T...... .........C .......A.. .......... .......... .......... T......... .......... .......T..

C.az .......... ...T...... ..C......C .......A.. .......... .......... .......... .......... .......... ..........

C.ko .......... ...T...... ..A......C .......A.. .......... .......... .......... .......... .......... ..........

C.va .......... ...T...... ..A......C .......A.. .......... .......... .......... .......... .......... ..........

L.ga .......... ...T...... ..CT.....C .......... .......... .......... .......... .......... .......... ..........

A.po .......... ...T...... ..CT.....C .......... .......... .......... .......... .......... .......... ......C...

B.pa .......... ...T...... ..C......C .......... .......... .......... .......... .......... .......... .......T..

L.la .....G.... ...T...... ..CG...... ......C... .......... .......... .......... .......... .......... ..........

P.oli .......... ...T...... ..C......C .......T.. .......C.. .......... .......... .......... .......... .......T..

P.ste .......... ...T...... ..C......C .......T.. .......... .......... .......... .......... .......... .......T..

C.lu .....G.... ...T...... ..CTC..... C......A.. .......... .......... .......... .......... .......... ......CT..

B.my .......... ...T...... ..CT..A..C .......T.. .......... .......... .......... .......... ....T..... ..G....T..

A.te CCGTCCTACT ATCTTACCTT TCCGTCTCGC AAGTTAGAGA ACTACTCTCT TTGGATCATA CTAAAACCTC TCAGTAGGCT TTCCGTAGTA TTTGCCTTCT

P.ii .......... .......... .......... .......... ..C....... .......... .......... .......... .......... ..........

C.az .......... .......... .......... .......... ..C....... .......... .......... .......... .......... ..........

C.ko .......... .......... .......... .......... ..C....... .......... .......... .......... .......... ..........

C.va .......... .......... .......... .......... ..C....... .......... .......... .......... .......... ..........

L.ga .......... .......... .......... .......... .......... .......T.. G......... .......... .......... ..........

A.po .......... .......... .......... .......... .......... .......T.. G......... .......... .......... .....G....

B.pa .......... .......... .......... .......... .......... .......T.. G......... .......... .......... ..........

L.la .......... .......... .......... .......... ......G... .......T.. G......... .......... .......... ..........

P.oli .......... .......... .......... .......... ......T... .......T.. .......... .......C.. .......... ..........

P.ste .......... .......... .......... .......... .......... .......T.. G......... .......C.. .......... ..........

C.lu ....A..... .......... .......... .......... .......... ...T..GT.. G......... .......C.. .......... ..........

B.my .......... G......... .......... .......... C......... .......T.. G......... .......... .......... ..........

A.te GTTGATGAAT CCAAATGTAC CCGAGATTAT TGTTTATCTA GGGCTATTTC TTTTCCCTGT TTTTTCTGTC TTCCAAAATC CAAGGTACTG CCGTCTCTGT

P.ii ..C...A... .......... .......... .......... .......... C....TT..C ...C...... .......... .......... ..........

C.az ..C....... .......... .......... .......... ........C. .....T.... ..G....... G......... .......... ..........

C.ko ..C....... .......... .......... .......... ........C. ........CC ..G....... G......... .......... ..........

C.va ..C....... .......... .......... .......... ........C. .....T.... ..A....... G......... .......... ..........

L.ga ......A... ..G....... .......... .......... .......... .C...TT... ..CC...... .......... .......... ..........

A.po .....CA... .......... .......... .......... .......... .C...TT.CC ..CC...... .......... .......... ..........

B.pa ..C...A... .......... .......... .......... .....G.... .....TT... ..C....... .......... .......... ..........

L.la ..C...A... .......... .......... .......... .....G...T ..C..TT... ..C....... .......... .......... ..........

P.oli ......T... .......... .......... .......... .....G.... ..C...T.CC ..C....... A......... .......... ..........

P.ste ......T... .......... .......... .......... .....G.... ..C...T.CC ..C....... A......... .......... ..........

C.lu .....CA... ..T....... .......... .......... .....G.... ..G...T..G .......... ..A....... .......... ..........

B.my ......A... ..G....... .......... .......... .......... CCC....... ..G....... ..A..C.... .......... ..........

A.te TTTCTTTTTT ATATCTTTCT CGGAATCCCC CCTCGCCGTT TTTCTGTTAA GTAGGTAGCA TCCCATCTCC CTCTGTCGTC TAGAATTCCT TGTAGTTTCT

P.ii .......... .......... .........G T......... .......C.. .C........ ...G...... .......... .......... ..........

C.az .......... .......... .......T.A ......G... .......... .......... .......... .......... .......... ..........

C.ko .......... .......... .........A ......G... .......... .......... .......... G......... ...G...... ..........

C.va .......... .......... .......T.A ...T..GC.. .......... .......... .......... G......... .......... ..........

L.ga .......... .......... .........T T......... .......... .......... ...G...... .......... .......... ..........

A.po .......... .......... ......GA.. T..T...... .......... .C........ ...G...... .......... .......... ..........

B.pa .......... .......... .......... T......... .......... .C........ .......... .......... .......... ..........

L.la .......... .......... .........T T..T...... .......... .C........ ...G...... .......... .......... ..........

P.oli .......... .......... .........T T......C.. .......... .......... .......... ..T....... ...T...... ..........

P.ste .......... .......... .........T T......C.. C......... .......... .......... ..T....... ...T...... ..........

C.lu .......... .......... ......T... G......C.. .......... .......... ...G.....T .......... .......... ....C.....

B.my .......... .......... ...G..A..T .......C.. .......C.. .C........ .......... .......... ...G...... ..........

A.te TTCTCTGCTA TGCCGTCTCC TTTTCTCCGA CGCGTGTTTC CCGCAGCAAT AGTATTAGTT ATTTCCTTTT CCTGTCACAG TGCCCTCAGT GTCTTGTGGT

P.ii .......... .......C.. .......... .......... ........G. .......... .......... .......... .......... ....C..C..

C.az .......... .A.....CT. .......... .......... .....C..G. .A........ .......... ..C....G.. ......G... .......C..

C.ko .......... .A.....CT. .......... .......... ........G. .A........ .......... ..C...G... .......... .......C..

C.va .......... .AG....CT. .......... .......... .....C..G. .A........ .......... ..C....G.. .......... .......C..

L.ga A......... .A..A..C.. .......... .......... ........G. .A........ .......... ......G... .......... .......C..

A.po .......... .A.....C.. .......... .......... ........G. ...G...... .......... ......G... .......... .......C..

B.pa .......... .A..T..C.. .......... .......... ........G. .......... .......... .......... .......... .......C..

L.la .......... CAG.A..... .......... .......... ........G. .......... .......C.. .......... .......... .......C..

P.oli .......... .A..A..C.. .......... .....C.... ........G. .......... .......... ..C..T.... .......... ....C..C..

P.ste .......... .A..A..C.. .......... .....C.... ........G. .......... .......... ..C..T.... ...T..T... .......C..

C.lu .......... .......C.. .......... .......... ........G. .A.G...... .......... ........G. ....T..... .......C..

B.my A......T.. .A..T..C.. .......... ..T..C.... .........C .......... .......... ......G... .....CT... T..C......

A.te AAAGACCGCC TGCATCCACT CCCTTTTCGG TTCTTTTCGA AACACACTCG AGTATTTTCT TATTTTTCTG CCATCTTATT TACCTTCCTT TTCGGGAACA

P.ii ..CC...... ..A....... .......... .......... ...G....A. .......... .......... ....T..... .......... ......G...

C.az G.CC...... .......... .......... .......... ...G...... .....C..T. .......... ....T....G .......... ..........

C.ko C.CC...... .......... .....C.... .......... ...G...... .....C..TC .......... ....T....G .......... ..........

C.va G.CC...... .......... .......... .......... ...G...... .....C..T. .......... ....T....G .......... ..........

L.ga ..CC..G... .AA....... .......... .......... ...G....A. .......... .......... ....T..... .......... ..........

A.po ..C...G... ..A....... .......... .......... ...G....A. ...T.C.... .......... ....T..... .......... ..........

B.pa ..CC..G... ..G....... .......... .......... ........A. ..C....... .......... ....T..... .......... .........C

L.la ..CC..G.T. ..G....... .......... .......... ...C...... .......... .......... ....T..... .......... ..........

P.oli ..TC..G... .ATG...... .......... .......... ...C....A. .....C...C .......... ....T..... .......... ..........

P.ste ..TC..G... .ATT...... .......... .......... ...C....G. .....C...C .......... ....T..... .......... ..........

C.lu GCCC..GCTT ..A....... .......... .......... ...C...... .....C..TC .......... ....T..... .......... ..........

B.my ..GCT.GA.. ..GT..T.A. .......... .......... ....G...G. ...T.C..TC .......... .A..T..... .......... ......G..C

A.te GTACGCATTT ACTCGCTCTT TCTTTTTAAC GCTCTTCTAA CAGTATTCAG AATTGCGGTT CTTT-ATCTA GTATGTCACA TACCTCGCTT TCCTTTATGG

P.ii ..C....... .......... .......... .......... .GC....A.. ..CG...... ....-..... .......... .......... ..........

C.az .......... .......... ....C.C... .........T G.C.....G. ...G..C... ....-..... .......... .......... ..........

C.ko .......... .......... ....C.C.G. .........T ..C.....G. ...G..C... ....-..... .......... .......... ..........

C.va .......... .......... ....C.C.C. ......T..T ..C.....G. ...G..C... ....G..... .......... .......... ..........

L.ga ..G....... .......... ....C..G.. .......... .GC....... ...G..C... ....-..... .......... .......... ..........

A.po ..G....... .......... ....C..... .......... .GC....... ...G..C... ....-..... .......... .......... ..........

B.pa .......... .......... .T..C....G .......... .CC.....T. ...G.T.... ....-..... .......... .......... ..........

L.la ..G....... .......... .......G.. .......... .GC.....G. ...G...... ....-..... .......... .......... ..........

P.oli .......... .......... ......A.C. .......... ..CC..C... ...G...... ....-..... .......... .......... ..........

P.ste .......... .......... ......A.C. .......... G.C.C.C..C ...G...... ....-..... .......... .......... ..........

C.lu .......... .......... ......C... .........T .CA.CCC.T. ...G..C... ....-..... .C........ .......... ..........

B.my ..C..A.... ...T...... .T..C.C..G .......C.T ..C..A..TC ...G.T.C.. ....-..... .......... .......... ..........

A.te AGTTGTTCTT CCTCAATCAC TTTTCTGGTT TCGCTGTGAC ATACTTCACC TGATGTTTGC TTTACCGCTC GCCTTTTCAG TCCCCTTGTC ATAAAGCAGG

P.ii .......... A......... .......... .......... .......... .......... .......... .......... .......... ..........

C.az .......... G...C..G.. .......... .......... .......... .......CC. .......G.. ..T....... .......... ..........

C.ko .......... G...C..G.. .......... .......... .......... .......CC. .......G.. ..T....... .......... ........C.

C.va .......... G...C..A.. .......... .......... .......... .......CC. .......G.. ..T....... .......... ..........

L.ga .......... A......G.. .......... .......... .......... .......... .......G.. .......... .......... .C........

A.po .......... A......G.. .......... .......... .......... .......... .......G.. .......... .......... .C........

B.pa .......... A...G..G.. .......... .......... .......... .......... .......G.. .......... .......... .C........

L.la .......... A......... .......... .......... .......... .......... .......G.. .......... .......... .C........

P.oli .......... A......G.. .......... .......... .......... .......C.G .......G.. ..T....... .......... .C........

P.ste .......... A......G.. .......... .......... .......... .......C.G .......G.. ..TC...... .......... .C........

C.lu .......... A......G.. .......... ...T...... .......... ........C. .......G.. .......... ....G..... .C......C.

B.my .......... A..G...T.. .......... .......... .......... .......... .......G.. ...C...... .......... .C........

A.te CTTTCGGTAT TTCTTCCGGT TGGTCATCTC CTCATTGATT TTCCTTGGCC GCTTTCGTGC TTCCCACTAT TTCCAGGACC CACTCTACCA CGAATTTCTA

P.ii .......... .......... .......... .......... .......... .......C.. .......... .......C.. ..T....... .......T..

C.az ..C..A.... .......... .......G.. .......... ..A...A... .......... ....G..... .......C.. ....A..... ..........

C.ko ..C..A.... .......... .......G.. .......... ......A... .......... ....G..... .......C.. G.T.A..... ..........

C.va ..C..A.... .......... .......G.. .......... ..A...A... ...C...... ....G..... .......C.. .G..A..... G.........

L.ga .......... ....C..... .......... .......... .......... .......... ....G..... ..T....C.. ..T....... ..........

A.po .......... ....C..... .......... .......... .......... .......... .......... ..T....CA. .......... ..........

B.pa .......... .......... .C........ .......... ......C... .......... .......... ..T....C.. ..T....... ..........

L.la .........C .......... .C........ ......C... ..T....... .......... .......... ..T....C.. .T..G..... ..........

P.oli .......... C......... .C........ .......... ..T...A..G ...C...C.. ....G..... ..T....CT. ..T....... ..........

P.ste .......... C......... .C........ .......... ......A..G ...C...C.. ....G.G... ..T....CT. ..T....... ..........

C.lu .......... .......... .C........ .......... ......A... .......... .......... .......CA. ..T....... ..........

B.my T......... .....G.... .CC....G.. .......... ......C... .......... .......... A.T....C.. A.TAA.C... .......T..

A.te TTCTTTTCTA CATTGGTCTT TACTCTCCCC CCTTTTTGTT TCCTTCCTGC ACCCGCATAT ACCTATCTTC GTTCTCTATA GGCAATTCTG CGTACACTAT

P.ii ........C. ........C. ...CT..... TT...C.T.A CT.CCT.CA. ..A...TC.. .........T .........C A....CC... .....C....

C.az .......TC. ........C. ...C.C.... TT.....T.A CT.CCT.CA. CAA...TA.. .........T .......... A.....C... .....C....

C.ko .......TC. ........C. ...C.C.... TT.....T.A CT.CCT.C.. ..A...TC.. .........T .......... A.....C... .....C....

C.va .......TC. .......G.. ...C.C.... TT.....T.A CT.CCT.CA. T.A...TC.. .........T ......C... A.....C... .....C....

L.ga ........C. ........C. .C.CTC.... TT...C.T.A CT.CCTTCC. G.....CC.. .........T .....T.... A.....C... .....C....

A.po .......T.. ........C. .C.CTC.T.. TT...C.TCA CT.CCTTCC. ..A...CC.. .........T .....T.... A.....C... .....C....

B.pa .C.....T.. ........C. ...CAC..G. TT...G.T.A CTTCCTACC. G....ATC.. .....C...T .........G ..A...C..T .....G....

L.la .......TC. ........C. .CACAC.... TT.....T.A CTTCGTTCC. G.A...CC.. .........T .....T.... A.T...C.C. .....C....

P.oli .......T.. ......G.C. CCA.T.T..G T....C.TC. CT.CCTTCC. GATA...C.. CT.......T .......... A...C..... ........G.

P.ste .......T.. ......G.C. AC.CT.T..G TTC..C.TCA CTTCCTGCC. .G.A..CC.. .........T .......T.. A...C...A. ..........

C.lu .......TG. ........C. ..A.A..... TT...C.T.A CT.CCTTCCG ....AGCC.. .........T .....T.T.. A......T.A ...GTCG...

B.my .C.....T.. .......... ...GA..T.T AT...G.T.A CTT.CGACA. .GGG..CC.. .....C...T .....T.T.. A.T...C..T T.CCTG....

A.te ATGTATATAC TTTTCTCTAT CGCTTATCCG ATACACATAG TTAATTTTTT CTTTTTCCAA TTATTTGGAG TGTTCTTTTG GGACGCACAC CCTACTTAAG

P.ii .......... .......... .......... .......... .......... .......... .......... .......... .......... ..........

C.az .......C.. .......... .......... .......... .......... .......... .......... .......... .......... ..........

C.ko .......C.. .......... .......... ........G. .......... .......... .......... .......... .......... ..........

C.va .......C.. .......... .......... ..C....... .......... .......... .......... .......... .....A...A ..........

L.ga .......... .......... .......... .......... .......... .......... .......... .......... .......... ..........

A.po .......... .......... .......... .......... .......... .......... .......... .......... .......... ..........

B.pa G......... .......... .......... ........G. .......... .......... .......... .......... .......... ..........

L.la .......... .......... .......... .......... .......... .......... .......... .......... .......... ..........

P.oli .......... .......... .......... .......... ......C... .......... .......... .......... ...G...... ..........

P.ste .......... ...C...... .......... .......... ......C... .......... .......... .......... ...G...... ..........

C.lu .......... .......... .......... ..G...T.G. .......... .......... .......... .......... .......... ..........

B.my T.A....... C......... ........T. ...A.....T .G........ .......... .......... .......... ..G....... ..........

A.te TGATGTTTCT CGTCTATACG ATAAT-TAGA CTATCTCTCG TTTCCCGACC ATGTACGTCC CTAGCCCTCC TTACGCTTTC GTTTTTGTGC TTGAAACTTC

P.ii .......... ....C..... .....TCCC. .....C..T. .......... .......... .......... .......... .......... ..A..C.C..

C.az .......... T...C..G.. .....C.CC. .......... .......... .......... .......... .......... ........C. ..A..C.C..

C.ko .......... T...C..G.. .....CCCC. G......... .......... .......... .......... .......... ........C. ..A..C.C..

C.va .......... T...C..G.. .....CCCC. T...G..... .......... .......... .......... .......... ........A. ..A..C.C..

L.ga .......... ....A..... ....CTCCC. G....A..T. .......... .......... .......... .......... .......... ..A..CTC..

A.po .......... ....A..... .....TCC.. A.C..C..T. .......... .......... .......... .......... .......... ..A.GC.C..

B.pa ........G. ....C..... .....TCCCC GC...A..T. C......... .......... .......... .......... .......... ..A..C.C..

L.la .......... ....CG.G.. .....TCCC. AC...A..T. C......... .......... .......... .......... .......... ..A.GCTC..

P.oli .......... .......... .....TCCC. A.......T. .......... .......... .......... .......... .......... ..A..CTC..

P.ste .......... ....C..... .....TCCC. A....A..T. .......... .......... .......... .......... .......... ..A..C.C..

C.lu .......... ....C..G.. .....C.CC. G....A..T. .......... .......... .......... .......... .......... ..A..CT...

B.my .......... ....A..... .....TCCC. GC...A..T. .......... .......... .......... .......... .......... ..A..G.C..

A.te TGTGTGCTCC TTCCTGCTCA AATAATTCTC CCGATGTTTT CTGTAACATC TTATGCACTT ACTTTTGCGC TTAGTAAAAA TGATGGTAAT CCTCCCGTCT

P.ii .......... .......... .......... .......... .......... .......... .......... .......... ........G. ..........

C.az .......... .......... .......... .......... .......... .......... .......... .......... .......... ..........

C.ko .......... .......... .......... .......... .......... .......... .......... .......... .......... ..........

C.va .......... .......... .......... .......... .......... .......... .......... .......... .......... ..........

L.ga .......... .......... .......... .......... .......... .......... .......... .......... .......... ..........

A.po .......... .......... .......... .......... .......... .......... .......... .......... .......... ..........

B.pa .......... .......... .......... .......... ....G..... .......... .......... .......... .......... ..........

L.la .......... .......... .......... .......... .......... .......... .......... .......... .......... ..........

P.oli C......... ....C..... .......... .......... .......... .......... .......... .......... .......... ..........

P.ste C......... ....C..... .......... .......... .......... .......... .......... .......... .......... ..........

C.lu C......... .......... .......... .......... .......... .......... .......... C......... .....C.T.. ..........

B.my C......... .......... .......... .......... ....G..... .......... .......... .......... .......T.. ..........

A.te GGTCTCGCCT TCGTTCAACT TACTACCATA CGCTCTCTTC CCTCCTAGTG TTAATCTGAC GTCGTCCTAA AACCTCACTA GTCGGGTTCG TTTCCATGTA

P.ii .......... .......... .......... .......... .......... .......... ...C...... .......... .......... .......CC.

C.az .C........ .......... .......... .......... .......... ..T....... ...C...... .......... .......... .......CC.

C.ko .C........ .......... .......... .......... .......... ..T..A.... ...C...... ......G... .......... ....G..TC.

C.va .C........ .......... .......... .......... .......... ..T....... ...C...... .......... .......... .......CC.

L.ga .......... .......... .......... .......... .......... .......... ..A....... .....T.... .......... ....A..CC.

A.po .......... .......... .......... ....T..... .......... .......... ..A....... .....T.... .......... ....A..CC.

B.pa .......... .......... .......... .......... .......... .......... ..G....... ..A..T.... .......... ....T..CC.

L.la .......... .......... .......... .......... .......... .......... ..AC...... .....T.... .......... .......CC.

P.oli .......... .......... .......... .......... .....C.... ..T....... ...C...... .....T.... .......... ...T...CCT

P.ste .......... .......... .......... .......... .......... ..TT...... ..GC...... .....T.... .......... .......CCT

C.lu .......... .......... .......... .......... .......... .....T.... .......... .G...T.... .......C.. ...TA..TC.

B.my .......... ....A..... .......... .......... .......... .....T.... ...C...... ...A.T.... .......... ...T...CC.

A.te ACCTTCTCCA TATCCTTTCC TGTTCCTATC CTCCAATATA CATCCAATCA TTGATCATCA GACCACGTTT GACTCGATTA ACGTAAGGCA CTCTGACCCC

P.ii ........TG .........T C......... .........C ...AT..... ...T..C.T. ....CTC... .....A.C.. ......T... ...G....T.

C.az ..A.....T. ......C..T ...CT..... ........CC ...T...... ......G.T. .TG..G.... .......... ......C... ..TCA.TT..

C.ko ..A.....T. ......C..T ...CT..... ........CC ....T..... ......G.T. .TG....... .......... ......C... ...CA..T..

C.va ..A.....T. ......C..T ....T..... ....G...CC .......... ........T. .TG....... .......... ......C... ..TCA..A..

L.ga .......... ......C..T C......... ...G....CC .G........ ...T..C.T. .....T.... .....A.... ......C... ...CA...T.

A.po ........T. .........T .......... ...G....CC .C........ ...T..C.T. C.T..TA... .....A.... ......C... ...CA.....

B.pa ........T. ......C... ....T..... ...G....CG .CC....... ......C.T. .....TA... .....A.... ......C... ...CT...T.

L.la .........C .........T .......... ...G.....C .G........ ...T..C.T. .T...TC.C. .....T.C.. ......C... ...ATG..T.

P.oli .....G...C ......C..T C...T..C.T ..TA.CC.CC ..GA.....T ...TA...T. .T...T.... .......... ......C... ...CC.T.T.

P.ste .........T ......C..T ....T..... T..A....CC .T.AT..... ...T..C.T. .T...T.... ..A..A.... ......C... ..TCC.T.TT

C.lu ........TG ...G..C..T .........T ...G....CC ......C... ......C.T. .....T.... .....A.... .T....C... ...CT...T.

B.my ........TC ......C..T ....TG...G T.GGGCA.AC AT.AT....C ......C.T. .GT.CTC.C. .TT..C.... T.....TC.. .C.CT.A...

A.te CCTGATAAGC CACTTCTTTT CTCTTTTT-T T------TCA TGACACTTAT CAACTTATCC CCATCCGGAT GCTTGTGTTC ATTCGTTTCT AACCATCCCT

P.ii .........T .......... ........C. .------..G .......... .......... .......... .......... .......... ..........

C.az .......G.T T......... ...C...CC. .------... .......... ..G....... .....T.... .......... .......... ..........

C.ko .......G.T T....G.... ...C...CC. .------... .......... ..G....... .......... .......... .......... ..........

C.va .......G.T T......... .C.....CC. .------... .......... ..G....... .......... .......... ..C....... ..........

L.ga .......G.T T.G....... ...C....C. .------... .......... .......... .....T.... .......... .......... ..........

A.po .T........ T......... ...C....C. .ATACGT... .......... .......... .....T.... .......... .......... ..........

B.pa ..C....G.. T.G....... ...C.A..C. .------... .......... .......... .....T.... .......... .......... ..........

L.la G.C....G.. T.GA...... ........C. .-------.G .......... ..G....... .....T.... .......... .......... ......AT..

P.oli ..C......T T....TC... ........TC .------..G .......... .......... .....T.... .......... .......... ..........

P.ste ..C....G.T T..A...... ......CCT. C------... .......... .......... .....T.... .......... ..C....... ..........

C.lu .......G.. T.G....... ...C..C.TG ATA----..G .......... .......... .....T.... .......... .......... ..T...AA..

B.my .G.....G.. T......... .C.C....TG A------..G ......C..C T.G....... .....T.... .......... ..G....... ......AT..

A.te CCTCATGGAT AAGGTTGATA CAGCCTTTTG TATATGGGTA AGCTTAAACG ATGTTTTTTT TTCCTTGATT CGGATCTGGC CTTCATTCCT CATGACTTAG

P.ii .......... .......... .......... .......... ...A...... .......... .......... .......... .......... ..........

C.az .......... .......... .......... .......... ...A...... .......... .......... .......... .......... ..........

C.ko .......... .......... .......... .......... ...A...... .......... .......... .......... .......... ..........

C.va .......... .......... .......... .......... ...A...... .......... .......... .......... .......... ..........

L.ga .......... .......... .......... .......... ...A...... .......... .......... .......... .......... ..........

A.po .......... .......... .......... .......... ...A...... .......... .......... .......... .......... ..........

B.pa .......... .......... .......... .......... ...A...... .......... .......... .......... .......... ..........

L.la ......C... .......... .......... .......... ...A...... .......... .......... .......... .......... .....A....

P.oli .......... .......... .......... .......... .......... .......... .......... .......... .......... ..........

P.ste .......... .......G.. .......... .......... ...A...... C......... .......... .......... .......... ....G.....

C.lu A.....C... .T........ .......... ..C..C.... ...A...C.. .......... .......... .......... .......... ..........

B.my ...T..C... .......... .......... .......... ...A...... .......... .......... .......... .......... .T..GA....

A.te TGGGTCTAAC CTCGTTCTAT TTCTTTTCCC TTATTTTAAC GCAACCGTGC ACAATCTACA TCAAATTGTC GTTTCTTTTC TTCCATTGAC AATCCCACTT

P.ii .......... .......... .......... .......... .......... .......... .......... T......C.. .......... ..........

C.az .......... .......... .......... .......... .......... .......... .......... C......C.. .......... ..........

C.ko .......... .......... .......... .......... .......... .......... .......... C......C.. .......... ..........

C.va .......... .......... .......G.. .......... .......... .......... .......... C......... .......... ..........

L.ga .......... .......... .......G.. .......... .......... .......... .......... C......... .......... ..........

A.po .......... .......... .......... .......... .......... .......... .......... C......... .......... ..........

B.pa .......... .......... .......... .......... .......... .......... .......... C......C.. .......... ..........

L.la .......... .......... .......G.. .......... ........A. .......... .......... T..C..GC.. .......... ...T......

P.oli .......... .......... ......C... .......... ........A. .......... .......... C.....CC.. .......... ..........

P.ste .......... .......... ......C... .......... ........A. .......... .......... T..C..CC.. .......... ..........

C.lu .......... ..A....... .......... .......... .....T..T. G......... ..T....... C...T..... .....C.... ..........

B.my .......G.. .......... .......... .......... .......... .AT....... AT........ T.CCT.GC.. ..A..A.... ..........

A.te CCCATACAGA TTTCACTTGC TCAATGGTTC TTTCTTTTTC TCTTACCAAG GTCTGCTCAC TTGCTTCATT TTCGTGGTCT AACATTTGAT CCTTATCTTT

P.ii .......... .......... .......... .........T .......... .......... .......... .......... .......... ..........

C.az .......... .......... .......... .........T .......... .......... .......... .......... .......... ..........

C.ko .......... .......... .......... .........T .......... .......... .......... .......... .......... ..........

C.va .......... .......... .......... .........T .......... .......... ......G... .......... .......... ..........

L.ga .......... .......... .......... .........T .......... .......... ...T...... .......... .......... ..........

A.po .......... .......... .......... .........T .......... .......... ...T...... .......... .......... ..........

B.pa .......... .......... .......... .........T .......... .......... ...T...... .......... .......... ......G...

L.la .......... .......... .......... .........T .......... ..T...C..T .......... .......... .......... ..C.......

P.oli .......... .......... .......... .........T .......... ..T......T .......... .......... ...T...... ......T...

P.ste .......... .......... .......... .........T .......... ..T......T .......... .......... ...T...... ..........

C.lu .......... .......... .......... .........T .......... .........T ...T...... .......... .C.......C ..........

B.my .......... .......... .......... ..C......T ..A..A.... ..T......T C..T..G... ......C... ...T...... ..CC..T...

A.te TTCCCTGGTA AACTTC---- CACAGGCTTG GTCCTGGCCT TACTGTCGGC TCTAGTTAAA ATTACACATG CAAGTATCTG CACACCCGTG AGGATT-CCC

P.ii .......... .....GA--- ..T...T... .......... .......A.. C........G .......... .......... ...C..T... .....G-...

C.az .......... ......A--- T.A...T... .......... .......A.. ........G. .......... ........A. ...C...... ......-...

C.ko .......... ......A--- ..A...T... .......... NG.....A.. .A......G. .......... ........C. ...C..T... ......G...

C.va ..T....... ......A--- T.A...T... .......... .GA....AA. ........G. .......... ........C. ...C..T... ......-...

L.ga ..T....... ......A--- T.A....... .......... .......... ......C... .......... ........C. .GTC...... .....G-...

A.po ..T....... .T....A--- T.A....... ......A... .......... ........G. .......... ........C. .GT....... .....A-...

B.pa ..T..C.... ......C--- ..A....... .......... .......... ......CG.. .......... ........C. .G.C..T... ......-...

L.la ..T.....C. ....C.A--- ..A....... .......... .......A.. .T....C... .......... .....C..C. .G.C..A... .....G-...

P.oli ..T.TC.... ..T.GGA--- ..A....... ......A.T. ........A. ....AC..G. C......... ........C. .C.C..T... ..A..G-...

P.ste ..T..C.T.. ..T.GGA--- ..A....... ......A.T. ........A. .T..AC.... C......... ........C. .C.C..T... ..A..G-...

C.lu G.T.TC.C.. ..T..A---- ..A....... .......... .......... .G..AC.C.. .......... ........C. ...C...... ..A..A-...

B.my ..T.TCC.C. ..T.A.TTGA ..A...T... .......... ....A..A.. ......C.G. .......... ........C. .G.C..T... .....A-...

A.te ACAGCCA--- --CCTATGTG GTGACGTGGA GTAGGTATCA GGCACGACC- ---------- --AC------ ----TCAGCC CAAGACACCT TGTTCAACCA

P.ii ..GAT..--- --..CCCAA. .....A.... .......... .....A...C --------AA AT.T------ ----.TC... .......... ......G...

C.az .TGCT..CCC CC..CCCCC. .....AC... .......... .....A...A CAGGTTAATA AAC.------ ----.T.... ......G... ....T.G...

C.ko GT.CT.---- -C..CC.CA. ....GAC... .......... .....A..TT ---ATAAGTT CAC.------ ----GA.... ......G... ..C.A.G...

C.va .TGCT..--- --...C.TA. .....AC... .C........ .....A.A.C CAGGTAATAA CAC.------ ----...... .......... ....T.G...

L.ga .TGAT.G--- --...TC.AT .....A.... ..T....... ...C.A...A CAACTGGC-G TA..ACCAGT ACCT...... .......... ......G...

A.po .TG.T.G--- --..C.CAC. AC...A.... ..T....... ...C.A...A ATGCTACTTG AA..CCTCTC GTAG.T.... .......... ....T.G...

B.pa GT.AT.T--- --T.CGAAC. AA.T.AC... .....C.... .....AC.-- ------CCCG AAGT------ --GGG..... ......G... ....T.G...

L.la ....T..--- --.....T.. .A...A.... ..C....... .....AC..A TTCACGGG-C AAG.TACTTG CCCTAG.... .......... ......G...

P.oli .T.A.GC--- --...GCTC. .GA..AA... .CT..C.... .....AGA.A T--------- ---------- --ATCTG... ..C...G... ..C.T.G...

P.ste ...A.TC--- --...GCT.. .GA..TA... .CC....... .....A.G.C C--------- ---------- --AGCT.... ..C...G... ..C.T.G...

C.lu .T....C--- --...T.A.. AG.G.A.... ..T....... ...G.AC--- ---------- -G..TAGATC TAATGT.... ..GA...... ..C.T.G...

B.my ...A..T--- --...GA... .A..A..... ..C..C.... .....TC..T -----GCTTA GGGTCA---- -AGA.T.... ......G... ....A.G...

A.te CACCCCCAAG GGTACTCAGC AGTGATAAAC ATTAAGCCAT AAGCGAAAGC TTTACTCAGT TAAGACTAAG -AGGGCTGGT -AAAGCCCGT GCCAGCCACC

P.ii .......... .......... .......G.. C......... G......... .......... .......... -.....C... -......... ..........

C.az .......... ....T..... .......G.. C......... .......... .......... C.TA.....T -......... -....T.... ..........

C.ko .G........ ....T..... .......G.. C......A.. .......... ......T... C.TA.....T -..A...... -....T.... ..........

C.va .......... ....T..... .......G.. C......... .......... .......... C.T......T -......... -....T.... ..........

L.ga .....T.... ....T..... .......G.. C......... G......... .......... .......... -.....C... -...A.T... ..........

A.po .......... ....T..... .......T.. .......... G......... .......... .......... -.....G... T...ATT... ..........

B.pa .......... ....G..... .......G.. .......... G......... .......... .......... -.....C... -T..A.T... ..........

L.la .....T.... ....T..... .......G.. C..G...A.. G......... .C........ C......... -.....C... -G..A.T... ..........

P.oli .....T.... ..A....... .........T .......... ...T.C..A. ..G...T... ....GT.... -.....C... -...A.T... ..........

P.ste .....T.... ..AC...... .........T .......... ...T....A. ..G...T... ....GT.... -.....C... -...A.T... ..........

C.lu .......... .......... ......TG.. .......... G.....G... .C........ ..GAGT...C C..A..C... -...A..... ..........

B.my .......... ....A..... .......... T......... G......... ......T... C........A -.....C... -...A.T... ......A...

A.te GCGGTTACAC GGACAGCCCA AGTTGACAGA CTGACGGCGT AAAGGGTGGT TAGGGAGATA TATAAACTAA AGCCGAAGAT CCCCAAGACT GTAGTACGTC

P.ii .......... ..G.G..... ........C. .-A....... ....A..... ......A.C. C..C...... .........C .......... ..G.....C.

C.az .......... ..G....T.. ........A. G-A....... ....A..... ......CTA- C.G....... ..A....AGA .T.......C ..T.C...CT

C.ko .......... .......T.. ........A. G-........ .......... ......ATA. .......... ..A....AGA .TT......C ..T.C...CT

C.va .......... .......T.. ........A. --A....... ....A..... .....GCCC. .TA....... ..AA...AGA .T.......C ..T.C...CT

L.ga .......T.. .AGAG..... .......... .-AG...... ....A..... ......A.C. C.CC...... ..T....AGC .......... ..CA....C.

A.po .......T.. .......... .........T .-AG...... ....A..... ......A... A......... .......AGC ..T....... .C.A....C.

B.pa .......T.. .AGAG..... .......... .-AG...... .......... ......AG.. CCCC.G.... .......AGC .GT....G.. ..GA....C.

L.la .......T.. .AGAG..... ......T.-. AC........ ....A..... ..A...A.C. .G-.T.A... .........C .TT..GAG.. ..CA....C.

P.oli .......T.. .AGAG....G .......... .-AG...... .......... .....G.T.G ACC....... G......CGC T.....AG.. ..TA..A.CA

P.ste .......T.. .AGAG..... ........A. .-A....... ....A..... .....GAT.T AC.......G .......CGC TTT...AG.. ..TA....CA

C.lu .......T.. ..GAG..T.. .......... ACC....... .......... ..A....GA. .......... ..T....AGA ATT..GAGT. ..GA..A.C.

B.my .......T.. ..GGG..... ......T..G .-TT.....C .......... ......A.C. AGA.T..... ..TT.....C ..T....G.C ..GA......

A.te ACCTCGGAGG AAAGAAGATC AACTACGAAA GTGGCTT--- ---------- ---------- ---------- ---TACAACA CCTGACCCCA CGAAAGCCAG

P.ii .T.....G.. .GT....... .........G .......--- ---------- ---------- ---------- ---......G .......... .......T.A

C.az G........C CCT...A... .C........ ...A...--- ---------- ---------- ---------- ---....... ..C....... .......TGA

C.ko G........C CCT...A... .T.C...... ...A...--- ---------- ---------- ---------- ---..T.CT. ..C....... .......TGA

C.va G........C CCT...A... .C.C...... ...A...--- ---------- ---------- ---------- ---..T.... ..C...G... .......TGA

L.ga ......AGA. C.CT....C. .......... ...A...AGG TCTGCGAGAG TTCAACGACT TCTGTTCAGA TAT..TTTT. .......... .......TGA

A.po .T....AG.A C.T.....C. G......... .......--- ---------- ---------- ---------- ---...T... .......... .......TGA

B.pa C.T...AT.. C.C.....C. G......... .......--- ---------- ---------- ---------- ---..ACCT. ......T... .......TGA

L.la T.TC..A..C CGT....CC. G......... .......--- ---------- ---------- ---------- ---...TTA. .......... .......TGA

P.oli C.--...GA. T.T...ACC. ..T....... ..A..C.--- ---------- ---------- ---------- ---..TTCAC .....A.... .......T.A

P.ste C.--..A.A. T.T...ACC. ..T....... ..A...C--- ---------- ---------- ---------- ---...CTAT .....A.... .......T.A

C.lu CA--..A..A C.T.....A. .C.C...... ...A...--- ---------- ---------- ---------- ---..AT..C ......T... .......TG.

B.my .T....AGA. G.C....C.. ..T.G....G ..AA...--- ---------- ---------- ---------- ---..TT.G. .....AG... .......TGA

A.te GGCACAAACT GGGATTAGAT ACCCCACTAT GCTTGGCCCT AAACATTGAA TAAATTTAAC CAATTTGTTC CGCCTGAGAA TTACGGACAC TAGCCTAAAA

P.ii .A.C...... .......... .......... ....A..... ......C... .G..C..... T....CA... ......G... .....A.... ...TT.....

C.az ..T....... .......... .......... ...CA.T.T. .......... .G........ ..T...A... ......GAG. C........T ...T......

C.ko ..T....... .......... .......... ...CA.T.T. .......... .......... ..T...A... ......GA.. C........T ...T......

C.va ..T....... .......... .......... ...CA.T.T. .......... .G........ ..T...A... ......GA.. C........T ...T......

L.ga ..G....... .......... .......... ...CA..... ......C... .G........ .....CAC.. ......G... .......... ...T......

A.po .......... .........G .......... ...CA..... ......C..T .G........ ..G..CA... ....C.G... .......... ...T......

B.pa ..A....... .......... .......... ...CA...A. .........T AG........ TG...CCA.. ....C.G... .......... C..T......

L.la .AA....... .......... .......... ...CA..... ......A..T CG........ ......TA.. ....C.G... .........T ...T......

P.oli ..A....... .......... .......... ....A..... ......C..T .GT.CA.T.. ACTCAACA.. ....C.G... ....AA..GT ...TT.....

P.ste ..A....... .......... .......... ....A..... ......C..T .GC...AT.. ACTCCATA.. ....C.G... ...T.A..GT C..TT.....

C.lu .A........ .......... .......... ...CA..... ......A..C .G..A..... AC...CTA.. ....C.GC.. C......... ...T..G...

B.my ..A....... .......... .......... ...CA...A. ......A..T AG........ T.T..CTG.. ......G.G. C........G CG.T......

A.te ATCAAAGGAC TTGGCGGTGC TTTAAACCCC CCTAGAGGAG CCTGTCCTAT AACCGATAAT CCCCGTTAAA CCTCACCTTC TCTAG--CCT TATCCACCTA

P.ii .C........ .......... ..A.G..... .......... .......... .......G.C .......C.. .......C.. ...C.--.TC ..........

C.az .C........ .......... .......... .......... ........G. .......G.C .......C.. .......C.. ...C.--... AGC....T..

C.ko .C........ .......... .......... .......... ........G. .......G.C .......T.. .......C.. ...C.--... AGC....T..

C.va .C........ .......... .......... .......... ........G. .......G.C .......C.. .......C.. ...C.--... AGC....T..

L.ga .C........ .......... ....G....A .......... .......... .........C .......... .......CC. C..T.--.TC ....T.....

A.po .C........ .......... ..C......A .......... .......... .........C .......... .......C.. C..T.--.TC ....T.....

B.pa .C........ .......... ....C.T..A .......... .....T.... .........C .......... .......CA. C..T.--... ..........

L.la .C........ .......... ..C.C.T..A .......... .....T.... .........C .......G.. .......CC. C..T.--..C GTC..G....

P.oli CC........ .......... ..A.C.T..A .......... .....T...G .........C .......... .......C.. ...T.--... C....G....

P.ste CC........ .......... ..A.C.T..A .......... .....T...G .........C .......... .......C.. ...T.--TT. .....G....

C.lu GC........ C.....A... ..C.C.T..A T......... .......... .......... .......... .......CC. C..T.CT... GTC..G....

B.my .C........ .......... ....T.T..A .......... .....T..G. .......G.. .......... .......C.. C..T.--.T. A...A.....

A.te TATACCGCCG TCGTCAGCTT ACCCTGAGAA GGGAAAGTAG TATGCAAAGT TGGCACAGCC CAAAACGTCA GGCCGAGGTG TAGTAAATGA GAAGGG-AAG

P.ii .......... C......... .......... ..A.T.A... ......G.A. .......... .T........ ..T....... ....GC...G ..G...-...

C.az .......... C.......C. .......... ..A.T.A... ......T.A. .......... .C........ ..T....... ....GC.... ..G..A-...

C.ko .......... C.......C. .......... ....T.A... ......T.A. .......... .T........ ..T....... ....GC.... ..G...-...

C.va .......... C.......C. .......... ..A.T.A... ......T.AC ...T.A.... .T........ ..T....... ....GC.... ..G...-...

L.ga .......... C......... .......... ..A...A.C. ......G.A. .......... .T........ ..T....... ....G....G ..G...-...

A.po .......... C......... .......... ..A.G...C. ......G.A. ...T..C... .C........ ..T....... ....G....G ..G...-...

B.pa .......... C......... .......... ..AG....C. .G.....GA. .....T.... .......... ..T....... .........G .TG...A.G-

L.la .......... C.......C. .......... ..ACT.A.C. ..C...G.A. .......... .......... ..T....... ...CG....G AGG...-...

P.oli ......A... .......... ......T... ..CTT.AC.. ..A....... .....A.... .......... ..T....... ....G..... .GG...-...

P.ste ......A... .......... ......T... ..ATTTAC.. ..A.....A. .......... .......... ..T....... ....G..... ..G...-...

C.lu .......... .......... ..A....... ....T.AA.. ..A.....AC .A...A.... ..G....... ..T....... C..CTC...G ..G...C...

B.my .......... C........C .......... ..A...A.C. .GG..GC.AC ......T... .......... ..T....... .........G ..G...ATTC

A.te AGATGGGCTA CATTTGCTAA ATACAAGCAA AC--ACGGAT GAAT-TATTG AAACATAATA CTGAAGGAGG ATTTAGTAGT AAGGGGGAAG CAGAGTGCCC

P.ii .......... ........CC CC.AG..... .TTT.....C .CC.-..... ........G. .......... .......... ...T.A...A ..........

C.az .......... ..C.....T. G.CT.....G .T--...... .TT.-.G... .....C..G. .......... .......... ..AT.A.G.A .........T

C.ko .......... ..C.....CG CCTTG..... .T--.....C .TT.-.G... .....CG.A. .......... .......... ..AT.A.G.A .........T

C.va .......... ..C.....T. .CCT...... .T--...A.. .TT.-.G... .....C..AG .......... .......... ..AT...G.A ..........

L.ga .......... ........T. CC........ ..--...A.. .TCC-C.C.. C....AGGG. .......... .......... ...CAT...A .......T.T

A.po .......... ........CT CCG....... .T--...... ATT.-..... ......G.A. T......... .......... ...CA....A .......T..

B.pa .A........ ........CT -.GA-..... .G--...... .TC.-CG... ......G.G. .......... .......T.. ...C..A..A T......TT.

L.la .A........ ........T. CCC....... ..--...A.. .TC.TCGC.. ...A.CG.A. .......... ......C... ...A...G.T .....C....

P.oli .A........ .......... .GCT-..... .T--...A.. A.TG-C.... ......GCA. T......... .......... ...CA..G.A T......T..

P.ste .A........ .......... T..T-..... ..--...A.. .TTG-C.... ......GCAG .......... ......C... ...CA..... T....C.T..

C.lu .......... ........C. C.TAG..... .T--..A... ACC.-CG... .....CG.G. T......... .......... ...CA..G.A .....C....

B.my G......... ........GT C.G.-..... ..--...... ATC.-..... ........GG T......... .......T.. ...C..A..A T......TT.

A.te CCCTGAAACC GGCCCTGAAG CGCGCACACA CCGCCCGTCA CCCTCACCAA GCACA--AGC ACGTCAATAA CTAATACATT AT----CTTG CACAAGGGGA

P.ii .A........ .......... .......... .......... .....C..G. ....C--.AA .TA.T.T... A...ACTGC. T.---A.C.. ..A....A..

C.az .A.......T .......... .......... .......... .....G..G. ...TT--.A. .AACTTTA.. T...ATA.CC T.---AAAC. ..TT...A..

C.ko .A........ .......... .......... .......... .....G..G. ...TT--.A. GAACATTA.. T-..A.AG.C T.---GAAA. ...T...A..

C.va .A........ .......... .......... .......... .....G..G. ...TT--.AA ..TATTT... ....A.GCCC T.---ATAC. ...C...A..

L.ga TG......TT .......... ........T. .......... .......... ....C--C.G .AAA..G... A.-.AG.CC. ..TCAA.AC. ..-.......

A.po .G......T. .......... ........T. .......... .......... ...TC--T.A ..AA.TC..T ..T.AG.CCC G.TCAATAC. ..........

B.pa .G......T. .......... ........T. .......... .......... ..C.C--CAG ..AG.CG... T...C.AGCC TGA--CA.GT GCG.......

L.la ........TT ...T...... .AA.....T. .......... .........T ....T--T.G CA.AAC.... G...A.TGAG CCCC-A..C. ..-.......

P.oli .G......TT ......A... .......... .......... .....C.... ..C.TAC.AG ..TAA.C... ....A.-CCA .CA--A.CC. .G-.......

P.ste .G.......T ......A... .......... .......... .....C.... ..C.C-CT.A ..TAATC... T...A.GCCA .CA--A.CC. .G-.......

C.lu T......... ...A...... .A..T..... .......... .......... ..G.C--T.G .AAA.CC... ....G.TGAG CCTA-A..-. ..-....A..

B.my .G........ .......... T...T...T. .......... .....G.TG. ..CTT---AG ...ATCG... A...CC.GC. T.T--AAGAA T..C.A....

A.te GGAAAGTCGT AACATGGTAA GTGTACCGGA AGGTGTACTT GGAAAATAC- ACTAAAGAGC TAGCCTAATA ATTAAAATGC AACAA-CCAA CATCAAT-AA

P.ii .......... .......... .......... .....C.... ...G....T- ..CC.TA... ........CC .CC....CA. .....-A..C ...T...-..

C.az .......... .......... .......... ......G... ..GT....T- ..CC.CC... .......CCC .CCC.C.... .....-...- ...T...-..

C.ko .......... .......... .......... ......G... ...C....T- ..CT.CT... .......CCC .CCC.C..A. .....-...- AC.T...-.C

C.va .......... .......... .......... ......G... ...T.....- ..CT..A... ......GTCC .CCC.CCCA. .....-...- TG.T...-..

L.ga .......... .......... .C........ .....CG... ...T.....- ..CC.TA... .....C..AC .CC....CT. .....-..CT ...T...-..

A.po .......... .......... .C........ .....CG... ...T.....- G.CC.CA... .....C..AC .CC...GAA. .....-G.CC .......-..

B.pa .......... .......... .C........ .....CG... ...T..ATAA G.CC.CT... ....TC..CC .CCC...CC. ...C.C..GC T..TC..T..

L.la .......... .......... .C........ ......G... ......AC.C ..CT...... ......G.CC .ACC...CTA .....-..C. T..T...-..

P.oli .......... .......... .......... .....C.... ......AT-- G.CC.TT... .....C..CC -CCTTC.CCT .....A..C. T..A...-..

P.ste .......... .......... .......... .......... ......AT.- G.CT.TT... .....CC.CC -CCTT..CA. .....A...C ...TC..-..

C.lu .......... .......... .......... .....C.... ..TC..G..- ..CC.CAG.. .....C..CC .ACC....C. .C...T..CC .T.....-..

B.my .......... .......... .C........ .....CG... ...C..ACAT G.....C... .....C..CC .A.T...A.. ...-.G...C AT.T...T..

A.te CCCCAAACAC CTTAAAAATA AAC---CTTA AAACATTCTT CCCCCTTAGT ATGGGTGACA AAAAAGGG-- AATAAGAGCT ATAGAACTAG TACCGCGAGG

P.ii ....CT.A.. .C....T.GG ...---.C.. .......... .....C.... .....C.... .......A-- .CC......C .......... ......A...

C.az A......T.. A.C..T...T C.AG-CA... .......T.. ..T..CC... ..A..C..TG G...C..ACC C...-..... .....TAG.. ..........

C.ko ....T..T.- GGC..CTTA. G..T-AA... .......T.C ..T..CC... .CA..A..TG ....C..ACC C..CT..... ......AA.. ..........

C.va A...C...G. ACC..TC.C. ...--AG... .......T.. ..T..C.... .CA......G ....C..ACC ....-..... .......A.. ......A...

L.ga .......... .CCCC.C.AC C.A--C.C.. .......T.. .....C.... .....C.... .......AG- .CA.G.C..C .......... ......A...

A.po A......T.. .CCC-.C.C. TT.--CAC.. .......... .......... .....A.... G......A-- .CA.G....C ......TC.. ......A...

B.pa ....CG.T.G .C.C.CCGC. .C.---.C.. .......T.. ..T.TC.... .....C.... G...GA.A-- CC..GA...G .....GA... ......A...

L.la ....CC.... AC..GGTTA. G..---ACC. G......T.. .....C.... ..A..C..T. G.......-- CCC....... ....C.AC.. ......A...

P.oli ....T..AG. ACGT..C.CC C..GTAGCC. ..C....... ..A...G... CCA..C..T. .......AA- .TCCG....A ......AA.. ......A...

P.ste ....T..AG. ACG...C.CC C..GTAGC.. ..C......C ......A... CCA..C..T. .......AA- .T.TG....A ......AA.. ......A...

C.lu A......... .CACC..TAC .CA---AG.. .........C ..T..C.... ..A..C.... G......A-- ..G.G..... ....C..... ......A...

B.my .....C.T.. .CAT..T..C TG.---.C.. .......T.C ..T.TC.... .....C.... G...GA.A-- CCC.G..... .....GAC.. ......A...

A.te GAACGCTGAA AGAGAGATGA AACAGAACAG TAAAGTAAAA CAAAGCAGAG CTACCACCTC GTACCTTTTG CATCATGAAT TAGCAAGTAC ACCCAGGCAA

P.ii ...A...... ......G... ..A....... ......CT.G AG......GA ...AAC..C. .......... .......... .........T .AA.......

C.az ...A...... ........T. ..T....... ......C.C. T......... ..TAT.G... .......... .......GGC ....C....G .A........

C.ko ...A...... ........T. ..T...C... .G....C... T......A.. A.TAG....T .......... .......GGC ....C....G TA........

C.va ...A...... ......C.T. ..G....... ......GCCT T......A.. ...AAC...T .......... .......GGC ....C....A GAG.......

L.ga ...T...... .....T.... .......... ......GT.. A......... ..TAA.G... .......... ........T. ....T..... .AG.GA....

A.po .......... .....AG... ..T.A.T... .....C.T.G AC......G. ..TAA...C. .......... ......TG.. ....T....A CAG.......

B.pa ...A...... ......G... ..A..TT... .....CG..G A......... A.TGGC.... .......... ........G. ....C....T .AG.GA....

L.la .......... ......G... .....T..CA .......C.G A......... A.TAAG.... ........C. .......... ....T..... TAT..A....

P.oli ...A...... .A........ ..T..CC... .....CTT.. T......... A.TAG.A... .......... ........T. ....T..C.. TTT..A....

P.ste ...A...... .......... ..A..CC... .....CTT.. AG.......A ..TAA.G... .......... ........T. ....T..C.. TTT..A....

C.lu ...G...... ....CA.... ....ACG.-- .........G A.C....... ..TA.C.... .......... ........G. ....T....T .....A....

B.my ...G...... .......... ..T..G...A .C...C..GG A......... ..TAAG.... .......... ........G. C.A.T....T .AG..A..G.

A.te GAAGCACC-C TAGCCTGACG CCCCGAAACT GGGTGAGCTA CTCCAGGGCC GCCTACTG-- AAGGGCAAAC CCGTCTCTGT GGCAAAAGAG TGGGAAGACC

P.ii .GG.G...-A C..T...C.A .......... .A........ .......A.. .....T.A-- C......... .......... .......... ........T.

C.az ........-A ...T...C.. .......... .......... ....GC.A.A .....T..-- C......... .......... .......... ........T.

C.ko ........-A C..T...TT. .......... T......... ....GC.A.A .....T.A-- T.....G... .......... .......... ........T.

C.va ..G.G...-A ...T...T.. .......... A......... .C...C.A.A .....T.A-- T......... .......... .......... ........T.

L.ga .G..G...-A .....C.C.. .........C .A........ .......A.A .....T.T-- C......... .......... .......... ........T.

A.po .G..A...-A .......CAA .......... .......... ....GA.A.A .....TAC-- T......... .......... .......... ........T.

B.pa ......A.-A .....C.C.A .........G AA........ .......A.A .....T.T-- T.....C... .......... .......... ........T.

L.la .G..A...-G ....T..TAC A......... .A........ .......A.A .....T.--- T......... .......... .......... ....G...T.

P.oli AG..A..TTA A..TT..TAA .......... .A........ .....A.A.A .....T.TA- T......... .......... .......... ........G.

P.ste AG..A...TA A..TT..TAA .......... .A........ .....A.A.A .....T.TA- T.....G... .......... .......... ........G.

C.lu .G......-A ....T...TA .......... .......... .......A.A .....TA--- T......... .......... .......... ........T.

B.my .GC..G..-A C..TT..C.C .........G AA........ .......A.A ....TT.TAC ......C... .......... .......... ........T.

A.te CTCGAGTTGA AGTGATAAAC TTACCGAACC CAGTAATAGC TGGTTGTCTG GAAAGTGTAT GTAAGTTCAG CCCCTTTTAT TCTT-TAGTT AAAAGCAGAA

P.ii .......A.. .......... .......... ....G..... .......... ....A..G.. A......... ....ACC.C. ....-..C.C .....T....

C.az G......A.. .....C.... .......... T...G..... ........CA AG.CA..A.. A......... .T.TCCCG.. .T..-.GA.C C.CT-.G.C.

C.ko G.T....A.. .....C..G. .......... T...T..... ........CA AG.CA..A.. A......... .T.TCCAAC. .T..-.GA.C C.CC-TG.CG

C.va G...G..A.. .....C..G. .......... T...G..... ........CA AG.CA..A.. A......... .T.TCCCC.. .T..-.GA.C T.GC-TG.C.

L.ga T......A.. .....C.... .......... .G..T..... .......... AG..A..G.. A......... .....CCCC. ....-.GT.C CTCG.T.ACG

A.po TC.....A.. .......... .......... .T..T..... ......C... .G.....G.. A......... ....ACC.C. ....-.TCCC .T..A..ACG

B.pa T......A.. .....C.... .........T .C..T..... ......C.C. ....A..G.. A.C....... .....CACCC C.C.CC.C.C .C.CA.CATG

L.la T......A.. .....C.G.. .........T ...CT..... ......C... AG..A..G.. A......... ....C.GAC. ....-C.C.C .T.C--GCCC

P.oli T.T....A.. G....C.G.. C........T ....T..... ......C.C. TG..T..G.. AG........ ...TC.GGG. ..CC-C.C.C .TGCA.T.TT

P.ste T.T....A.. G....C.... C........T T...T..... ......C... TG..T..A.. AG........ ....C.GG.. ...C-C.C.C .TGCA.TTT.

C.lu ..T....A.. .....C.G.. .........T ....T..... .......... AG..A..G.. A.T....... ...TCC.CC. .T..-ACC.C .TGG--GCC.

B.my .......A.. .....A.CG. .........T TC..T..... ......C... .G..A..A.. A.T....... ......A... ....AC.C.C CCC.AGGA..

A.te TCT-GCAAGT CT--AATATT AAGAA--TAA ATGGGAGTTA GTCGAAGGGG GTACAGCCCC TTCGATGAAG GAAACAACCT TTAAA-GGAG GATAAAGATT

P.ii AA.-A.C.C. A.--G.C.AA .....--G.. GG........ C..A...... .......... ..T..GAG.A ........T. ..T..-.C.. .....G....

C.az .GC-..CCT. -.--G.CCCA ...T.TTA.. GGA.A..... C......... .......... .....G.G.A ........T. ..TC.T.... .T...G....

C.ko .GC-..C.T. -.--G.CCCA G....CACC. GGA.A..... C..A..A... .........G ..T..G.G.A ........T. ..TC.T.... .T...G....

C.va CTC-..CCC. -.--G.CC.A .....-GG.. GGA.A..... C.....A... .......... .....G...A ........T. ..T..T.... .T...G....

L.ga CAATA.C.T. A.--G.C.A. ...G.--G.. GG........ A..A...... .......... ..T..AA... ..T.....T. ..T.G-.... ..........

A.po .A.AA.--T. G.--GGC.A. .....--GGG .G........ ...A...... .......... ..T..AT... ........T. ..T..-.... .....G....

B.pa .TAT.GT.T. A.--G.C.GC G.CGGAGGCC .-........ T..AC..... .......... .CT..ACC.A ..T.....T. .CT..-.... ..A..G....

L.la C-----CC.. A.--G.C.AA ....TTTATT .G........ T..A...... .........G ..T..AA..A ..C.....T. ..T.G-.... .....G...A

P.oli AT.A-.CCA. .A--G..GCA .C..GAAACT .GA..T.... ...A...... .......... ..T..AAC.A ..C.....T. ...C.-.C.. .........C

P.ste .T.AA.CCT. .A--G.CGCA GC..GAAACC .G...-.... ...A...... .......... ..T..AAC.A ..C.....T. .CCC.-.C.. .........C

C.lu CAGT..TCT. A.--G...AA .....GGG-- GGA....... .......... ..T....... ..T..AA..A ..C.....T. ..T..-.... .........A

B.my .TACCAG.A. G.AGG.C.GC T.A.GACAT. .A........ T..A...... .......... .....ATT.A ..C.....T. .CT..-..T. .T...G....

A.te ATA----ATA CCCAAGAGT- TAGCGCCCCA GTAGGCCTAA GAGCAGCCAC CTGAATAGAA AGCGTCAAAG CTTAAGCACA C-----TTTA ACCCTATTAT

P.ii .A.----... TTT...G.A- ...TAA..T. ..G....... A......... ..AT...... .......... ..C..A...G A-----C.CC G...C.....

C.az ..G----..T .A.TG.G.A- CTCT....T. ..G....... .......... ..ATGC.... ......G... ..CG....GG A-----C.AC ....C.....

C.ko ..G----..T GA.CG.G.C- .TCT....T. ..G......G .......... ..AT...... ......G... ..CG....GC A-----..-T T...C.....

C.va ...----..T TG..G.G.A- CTCT....T. ..G....... .......... ..A.GC.... ......G... ..CG....GG AACCCCCCCC C...C.....

L.ga ...----... TA....GAA- GG.TAG.... ..G....... A......... ...T...... .......... ..C....... A-----CACC ....GCA...

A.po ...----GCC TT....G.A- .G.TAG.T.. ..G....... A......... ......C..G G......... ..C....... A-----CACC ....GCA...

B.pa ...CTAC.AG AG....G.AC CT.....T.. ..G....... A......... ...G.CC... .....T.... ..C....G.. AA----ACC. G...CCC...

L.la -CG----T-- --G...GAC- .C.T.....C ..G.....G. AG........ .CCCGC.... .....T.... ..C....... G-----CAAC ....ATA...

P.oli ...----C.C AATC.AG.AC A.AT.TTTT. ..G....... A......... ..T..C.... .....T.... ..C.GA..T. G-----ACAC CT..ATA...

P.ste ...----T.C AAAT.AG.AC AGAT.TTTT. ..G....... A......... ..T..C.... .....T.... ..C.GA..TG A-----AGCC CT..CG....

C.lu GC.----.-- --.C.AG.G- C.....TT.. ..G.....G. A........A ..T.GC.... .....T.... ..C....G.. A-----CA.. ....ATAA..

B.my ...C-AC.CT TAT...G.-- ..ATA..AT. ..G.....G. A........T ..ATTA.... .....T.T.. ..C....G.. AA----AGC. ....ATAC..

A.te ACCATTAAAC TCAAACAGAA GCCCCAACCT TAAACCAGAC CCTCCTACTT G--TAGTGGG AGAGAATATG CTAGTATGAG TAATAAGAG- GCC-TAAG-A

P.ii ....A..... --.GC.CA.. .......A-. A.C....... .TCT.C...G C--A.....A G.....G... .......... ........A- A..-C...-.

C.az ....G...GT A-..C.CA.. A......AT. AGT.TTG... ..GT...... A--A...A.A GA...TC... .......... ........A- ...A.C..-.

C.ko ....C....T A-..C.C... A.......T. .GT.TTG... .ACT...... .--A...A.A .A...TCC.. ....C..... ........A- .T.A....-G

C.va ...TA..G.T A-TCC.C... A.......T. .GT.TTGA.. ..CT...... .--A...A.A GA...TC... .......... ........A- ...A....-G

L.ga C...AC.... C-CCT.C... .........C ..T....... .T.T.C..GC A----....A ......C... ...A...... ........AA ...CCG.---

A.po C...A..... C-C.T.CA.. .....G-..C ATT.....G. .T.T...TAC C--CCA.A.A ......C... .......... ........AA A..T.G..-.

B.pa ..T.A...GA C-T.T.CC.. .....C.AAC -CT...G.G. TT.T.....C A----..A.A ...A..A... ....C..... ...C.....- --GG..C.-C

L.la T...AC.... C-CGT.CA.. .....G..AC C.T.T...G. TGCT....C. AACCC..A.A ...A.TA... .......... .....G..AA T--CC.C.-.

P.oli ...GA...C. .-T.T.TT.. T....T.AA. -TT.A.GAG. ..C....TGC CAAACA.A.. GAT..CC... ...A...... .........A ATAC....T.

P.ste ...GA...C. .-T.T.TT.. T....C..A. -TT.A...G. .......TGC ATCACA.A.. .AC..C.... ...A...... ........AA .TA-...CC.

C.lu CT.......A C-TTT.TA.. .....-.TAA .TT.T..... .T.T.C..C- ---CT....A ......C... ...A...... ...C....AG A.AC..C.-.

B.my G...G....A C-T.T.CA.. A....GT.AC -GG.....G. .T.T.C..AG A----....A ..G....... ..GA...... ...C.....- --ACCCCA-C

A.te ATCTCTCCCC GCACGCGTGT AAGTCGGCAT GGACCACCCA CCGACAACTA ACGGACCCAA ATAA-AGAGG GTAATGAAGG ATAA----GA C----ACTGG

P.ii G.T......A ....A..CT. ...C.A...C ....A..... ....A..T.. .......... .G..-..... .C......A. .CG.CG--A. .T--A...A.

C.az GCT.....AG ...TA..... .CAA.A.A.C ....A.A... ....A..T.. .......... .GT.-..... .......TAT C..TTT--A. GC--A..C..

C.ko .CT......G ....AA.... .TAC.A.A.C ....A.A... .T..A..T.. .......... CGCC-..... ..G.....AT C..CCT--.. TA--A.....

C.va GCT......A ....A..... .TAC.A.A.C ....A.A... ....A..T.. .......... CAGC-..... .......TAT C.GCCT--.. GC--G...A.

L.ga C.T.....TA ....A.A... ..C..A...C ....A....G ....A..T.. .......... .AG.-..... ...C...CA. ...CCA--C. .C--A..A..

A.po C.T.....T. ....A..... ..CC.A...C ....A..... ....A..T.. .......... TGC.-..... .C.....CAA GC..CG--C. GT--G..C..

B.pa .C.......G ....A..CA. ..A..A...C .....CG..G ....AG.T.. .......... CA..-..... .A.G..GCAA GA.GCA--.. .C--G.....

L.la ..T......G ....T..C.. ..A..A.A.C ....A..... ....GC.T.. .......... GAG.-..... ...C..---A ..TGCC--C. .----..C..

P.oli C.......T. ....AT.... ..A....A.C .....C.... .....TCT.. ....C..... TC..-..... .A.C..G.AA ..GTCATC.. TT--G...A.

P.ste C.T.....TT ....AT.... ..A....A.C .....C.... ....ATCT.. ....C..... TC..-..... ...T..G.AA CC.CCACAA. TTTAGG.CA.

C.lu GCT....... ....A.CCA. ..A....A.C ....A..... ....AT.T.. .......... .AC.T..... .CCC..GGAA .C.GCA--A. TG--A.....

B.my G........A ...TAT.... ..A.....GC ....T.G... ....A..T.. ....C..... TC..-T.... ........TC .C.GTA--.. TC--AT....

A.te AAAATAACTC ACCC------ TCCCCCGTTA ACCCCACACT GGTGTGCAAC A-GGGAAAGA CAAAAGGAAT AGAGAAGGAA CTCGGCAAAA C----CACAA

P.ii ..G.AC.... ....------ C.A......G .......... .......CC. .-A....... .......G.- .......... .......... TA---TC.C.

C.az ..G.GC.T.. ..A.-----C CA-......T .........C ..A....CCG .-A....... .......... .A........ .........C T-------TT

C.ko ....GC.T.. .TA.-----C CAA......T ....T....C ..A....CCT .-A....... .......... .......... .........C T-------TC

C.va ....GC.... .TA.ATACAC CT.......T .A........ ..A....CCA .-A....... .......G.. TA........ .........C T-------T.

L.ga ....AC.T.. .A..------ C.G....... .........C .......TCA .-A....... .T...A...- .......... .........C AA----.TT.

A.po ....AC.T.. .A..------ C.AA...... .........A .......TTA .-A....... .T...A..G- G......... .........C AATCCT....

B.pa ....AC..C. ....-----C .A-....... .......... .......CC. .-A....... .T.....G.- .A........ .........C .G------C.

L.la ....GC.T.. GA..-----T CAAA.....G .........C ..A....CTA .-........ .TG......- .......... .......... TG----.AG.

P.oli G...AC.TC. .ATA-----C AA........ .......... .......CC. .GA....... .CC....GG- G......... .........C ATA--.C.C.

P.ste ....AC.TC. .ATG-----A AAA....... .......... .......CCA .AA....... .C...A.GG- G......... .........C ATA--.C.C.

C.lu ..G.CC..C. ....-----C CAG......G .......... ..C.....TG .-A....... .T...A..G- .......... .........C AA----.TG.

B.my .....C.A.. .TTA-----C .AT....... C...T..... ...A...CCG .-A...G-.. .T...A..G- .A........ .........C TAA----TT.

A.te AGCCTCGCCT GTTTACCAAA AACATCGCCT CTTGCAAAA- TTA---AGAA TAAGAGGTCC AGCCTGCCCA GTGACATTTT ---TTAACGG CCGCGGTATT

P.ii .......... .......... .......... ........C- A..TAT.... .........T GA........ ......C.A. -GT.C..... ..........

C.az .......... .......... .......... .....G.G.- A-.TTT.... .......... .A........ ........A. -GT....... ..........

C.ko .......... .......... .......... ........C- C-.TAT.... .......... .A........ ......C.A. -GT.C..... ..........

C.va .......... .......... .......... .......TT- A-.CAT.... .......... .A........ ........A. -GT....... ..........

L.ga .......... .......... ....C..... .....C..C- AC.TTT.... .......... .A........ .......--. -GT....... ..........

A.po .......... .......... .......... .....G..C- AC.TTT.... .......... .A........ ......CCC. -GT.C..... ..........

B.pa .......... .......... .......... ........C- GCTTGT.... .........G .......... ......C.A. -GT.C..... ....A.....

L.la .......... .......... .......... .....T..C- GCTTGT.... .......... .......... ......A.G. -GT.C..... ..........

P.oli .......... .......... .......... .......G.- --CACA..T. .......... C......... .......CA. AGT.C..... ..........

P.ste .......... .......... .......... ......T..- --CCAC..T. .......... C......... ......AC.. AGT.C..... ..........

C.lu .......... .......... .......... .....T..CT ACTTGT.... .........T .......... ......A.A. -GT.C..... ....A.....

B.my .......... .......... .......... .....T...- ACCT--.... .......... .......... ......C.A. -GT....... ....A....A

A.te TTGACCGTGC TAAGGTAGCG CAATCACTTG TCTTTTAAAT GAAGACCTGT ATGAAAGGCA TCACGAGGGC TTAACTGTCT CCTCTTTCCA GTCAATGAAA

P.ii .......... .......... .......... .......... A.G....... ........T. .G........ ..G....... .....CC... ..........

C.az ..A....... .......... .......... .......... .......... ........AC .A........ .C.G...... ...T...... ..........

C.ko .......... .......... .......... .......... .......... ........AC .A........ .G.G...... ...T...... ..........

C.va .......... .......... .......... .......... .......... ........AT AA........ .C.G...... ...T...... ..........

L.ga .......... .......... .......... ...C...... .GG....... ........T. .A........ ...G...... ...T...... ..........

A.po .......... .......... .......... ...C...... .GG....... ........T. .A........ .......... ....CC.... ..........

B.pa ..A..T.... .......... .......... .......... ..G....... .........G .G........ .......... ...T..C..G ....G.....

L.la .....T.... .......... .......... .......... .GG....... ........T. .T........ .......... ........T. ..........

P.oli .......... A......... T......... .......... .......C.. .....T.... .A........ ...G...... ...TCCC... ..........

P.ste .......... A......... T......... .......... .......... .....T.... .A........ .......... ...TCCC.TG ..........

C.lu .....T.... .......... .......... .......... .......... ........T. .G........ ..G....... .....C...G ....G.....

B.my ..A..T.... G......... .......... .......... ....G..... .........G .A........ .......... ...T....AG ....G.....

A.te TTGATCTCTC CGTGCAGAAG CGGGGATTTA AACATAAGAC GAGAAGACCC TGTGGAGCTT GAGACG-CTA GCGCAGCCCA TGTAAA-ACA CCCAATGTTC

P.ii ........C. .......... .......C.T .......... .......... .......... .....C-.AG .T.TG..A.. ...T..-.A. AA.TT.AC..

C.az ........C. .......... .......CCC .......... .......... .......... A..G.C-.C. .TAT...... ...T..T.A. .T.TCAACA.

C.ko .....T..C. .......... .......G.. C......... .......... .......... A..G.C-.C. ..AA..T... ...T..TGA. GT.TT.A.GA

C.va ........C. .......... .......C.C C......... .......... .......... A....C-TA. ..AA...T.. ...T..T.AG .A.TTAACG.

L.ga ........C. .......... .........T .......... .......... .......... A....A-.C. .A........ C..C..--AG A..TCC.CC.

A.po ........C. .......... ........CT .......... .......... .......... A....--.C. .A.....T.. C..C..--TC AA.CGCAC..

B.pa ........C. .......... .......CG. .C........ .......... .......... ......-TC. .G....T... ......C.GC .TTTCCCCCA

L.la .......... .......... .......C.. GC........ .......... .......... .....A-.C. .A........ C..CC.--A. A..CCATC-A

P.oli ........C. .......... .......AAC CT........ .......... .A........ T.....-.A. .G....AT.. ...C..ATAC A..C.GC.AA

P.ste ........C. .......... .......GA. .C........ .......... .A........ T....A-.AC AG.TG.A... ...C..ATAC ...C.GC.AA

C.lu ........C. .......... ........C. .G........ .......... .......... A....AA.CG TG.T...... ...C..--GG AT.CCACC.A

B.my ........C. .......... ........A. C..G...... .......... A......... .....A-AC. .G........ .A.C.CC.GC .TTTT----A

A.te CAGGAATCAA --CTGA-TGG GGACTGCGCC TTTGTCTTCG GTTGGGGTGA CCACGGAGTA AGAATAACCA CCGTGCAGAC AGGGAGCAAA T-AGGGACAA

P.ii A.A.G.CA.. --..A.G..A AAC.CA.A.. .A........ .......... ...T..G... GACC.....C ..A..A.... ........C- .-..A.GA..

C.az A.ACC..A.. --..A.A..A .A.....TA. .GG.C..... .......... ...T..G... TA....G..C ..AA..G... ........C- .-.CA.GA.C

C.ko GGACT.CA.. --..A.A..A AT...AGTG. .GG.C..... .......... ...T..G... GA..C.G..C ..AG..G... .....A..C- .-GAA.GA.T

C.va A.ACT.GA.. --..A.G..A .T...AA.A. .AG.C..... .......... ...T..G... .A..C....C ..AA..G... .....A.TC- .-GCA.GA.T

L.ga .C..CCAG.. --....A... ..C....T.. .A........ .......... ...T..G.A. ..C.A....C ..A..TG... ........CT A-GA...A.C

A.po A....T.A.. --.CAGG..A .AG..C.T.. .C........ .......... ...T..G.C. .ACCC....C ..A..T.... ....G...CT A-GA.....C

B.pa AG.CC.A... GCA.A.T-.. .CC..A.C.T AG........ .......C.. ...T..G.A. TATCA....C ..A..A...A T.......CT AAC.A.GG.T

L.la A....CCG.. --....G... CAT....C.. AA........ .......... ...T..G.A. ..C.A....C ..AC..G... C.......C- .-..A.GA.C

P.oli GG.CCC.G.. --..A.A..A AAC.A..C.T GA........ .......C.. ...T..G.A. CAC.A....C ..AC.TG..A .A....T.C- ----------

P.ste GG.CC-.G.. --..A.A... AAC....CTT GA........ .......C.. ...T..G.A. TAC.A....C ..AC.TG..A ........C- ----------

C.lu AG....AG.. --..A.T... ACC....C.T AG......A. .......C.. ...T..G.A. CACCC....C ..A..AG... C.......C- .-GCA.GG..

B.my AG.AGC.... GCT..CT... ATC....C.. AA........ .......C.. ...T....G. .ACCC....C ..A..T.... T....C.CCC C-T..A.G.C

A.te TTACCCCCA- --CCCACAAC TGCCCTCCTC AAAACCAGAG CTCCGGCTCT AGTTAA-CGA AA-CTTTCGA CTTAAAATGA TCCGGGAT-- AACCGATCAT

P.ii .CTT..AT.A A-TTT..... .C.......A ...G...... .C..AC.... ..C...-... ..-.A..... ...G...... .......--- .........A

C.az ..T.A.AT.A A-TA..T... .C.A...... ...G.A.... .CG.AA.... .AC...-... ..-....... ..C...C... ......TATA T........A

C.ko ...A..AT.A A-.A..T... .C.A...... .....A.... .CT.AA.... .AC...-... ..-T...... ..C...G... ......TG-A T........A

C.va ...A..AA.A A-.A..T... CCTA...... ...GTA.... .CT....... ..C..G-... ..-....... ..C...G... .....AC.-A T........A

L.ga ..CA..A..C AC........ .T.A...... C......... .C........ ......-... ..-TG..... ....G..... .......A-- .........A

A.po C.TA..A..A AT.......T .T.A....CA C......... .......... ......-... ..CTC..... ....G.T... ......C.-- .........A

B.pa C.C...A.CA AA........ GC.G.....A G......... .C..C..... G..C.G-..G ..-TA..... ....G.C... .....-.C-T .........A

L.la ..TT...A.C --.AT..... CAAA.....A ...G.T.... .C..AA.... ..C...A... ..-.A..... .C..G.C... .....C..-A -G.......A

P.oli --.....T.C ATT-.----. .CT......A C..G.T.... .GA.A..... .A.C.GCA.. ..--..CT.. .C-...C... .....C.A-- CG.......A

P.ste --.....T.A GTTA.----T .CTT....CG C..G...... .AA.A..... .ACA.GCA.. ..--G.CT.. .C-...C... .....T.A-- .........A

C.lu AAT......G --..TTTG.. CA.......A G.....C.G. .......C.. ....C.-... ..-AA..... .C..T.C... .....CCC-A .G.......A

B.my A.CT.TA.CA AA..T----T A.TA.....A G....T.... GCA.T....C .A....-..G ..-TA..... ..A.C.T... .......C-A .C.......A

A.te CGAACCAAGT TACCCCAGGG ATAACAGCGC TATCCCCTTC AAGAGCCCAT ATCGCCAAGG GGGTTTACGA CCTCGATGTT GGATCAGGAC ATCCTAATGG

P.ii .......... .......... .......... .......... .......... .......... .......... .......... .......... ..........

C.az .......... .......... .......... C......... T......... ....A..... .......... .......... .......... ..........

C.ko .......... .......... .......... C......... T......... ....A..... .......... .......... .......... ..........

C.va .......... .......... .......... C......... T......... ....A..... .......... .......... .......... ..........

L.ga .......... .......... .......... .......... C......... .......... .......... .......... .......... ..........

A.po .......... .......... .......... .......... C......... .......... .......... .......... .......... ..........

B.pa .......... .......... .......... C......... C......... .......... .......... .......... .......... ..........

L.la ..G..T.... .......... .......... .......... C......... .......... .......... .......... ........G. ..........

P.oli .......... .....T.... .......... A........T T......... ....A..... .......... .......... .......... ..........

P.ste .......... .....T.... .......... A........T T......... ....A..... .......... .......... .......... ..........

C.lu ..G....... .......... .......... ........C. C.....T... ....A.G... .......... .......... .......... ..........

B.my ....AT.... ......T... .......... A.......C. C....T.... ......G... .......... .......... .......... ..........

A.te TGCAGCCGCT ATTAAGGGTT CGTTTGTTCA ACGATTAA-A GTCCTACGTG ATCTGAGTTC AGACCGGAGC AATCCAGGTC AGTTTCTATC TATGAAAACG

P.ii .......... .......... .......... ........-. .......... .......... .........T .......... .......... ......CG.A

C.az ....A..... .......... .......... ........-. .......... .......... .......... .......... .......... .....TCTAA

C.ko .......... ...G...... .......... ........-. .......... .......... .......... .......... .......... .....CC.A.

C.va ....A..... ...G...... .......... ........-. .......... .......... .......... .......... .......... ......C.G.

L.ga .......... .......... .......... ........-. .......... .......... .......... .......... .......... .....CT...

A.po .......... .......... .......... ........-. .......... .......... .........T .......... .......... ......CG..

B.pa .......... .......... .......... ........-. .......... .......... .......... .......... .......... ....-.C..A

L.la ....A..... .......... .......... ........-. .C........ .......... .......... .......... .......... ....-.CGT.

P.oli .......... .......... .......... ........-. .......... .......... .........T .......... .......... ....-TG.T.

P.ste .......... .......... .......... ........-. .......... .......... .........T .......... .......... ....-.C.T.

C.lu ....A..... .......... .......... ........-. .......... .......... .........T .......... .......... ....G.G...

B.my ....A..... .......... .......... ........T. .......... .......... .......... .......... ..C....... ..C.-CTG.A

A.te CTTTCTTCCA GTACGAAAGG ACCGAAGAAA GGGGGCCCAT GCTTATAAGC ACGCCCAACC CCC-ACCCGA TGAAGCCAAC TAAACTGGGT AAAGGGG-AC

P.ii ..C.....T. .......... ........G. .A......C. ...CCCC... ......G... .TT-..TT.. .....T.... .....CAAA. .......CG.

C.az ........T. .......... .......... .......... .T.A.-.... ......C... .T.-.T.T.. .A..TTACCA .....CA.A- ..G....A.G

C.ko ........T. .......... .....G.... .......... C..A.-.... ......C... ...C.T.T.G .A..C.AGGA .....CA..- G.....AA.G

C.va ........T. .......... ........G. ........C. ...A.-.G.. ......CG.. .A.-CT...G ....CTTG.A .....C...- .C.....ATG

L.ga ......C... .......... ....G....G .......... ..C.TCGG.. ......G... ...-...T.. .....T.... ...G.CA..C ..G....CGT

A.po ......C... .......... ....G..... .......... ....GAG... ......GC.. ...-...T.. .....A.... .....CA..C ..G...ACG.

B.pa T....C..T. .......... .....G.... A......... ....TAC... .......... ...-...T.G ...CAA.... .....CAT.C ..C....TGT

L.la T...T...T. .......... ....G...G. A......T.. ....TC.... ......G... T..-..TT.. ....AA.... ....TCAA.. ..G....C..

P.oli A.C.T...T. .......... ......A.G. A......... ...CT-T..T .....TC... ...-...TA. ....AAA.T. ......A... ...A...C.T

P.ste A.C.T...T. .......... ......A.G. A......... ...A.-.G.T .....TC... ...-...TA. ....AAA.T. ......A..C ...A...C.T

C.lu ....T...T. .......... .......... .A........ ....CCC..T ......T... ...-...T.. ....AA.... ....TCA..C ..G.A..CG.

B.my .....C..T. T...A..... ......A... .......... .T..TAT..T .A........ ..T-.TTA.G .....A..T. .....CTT.- ..G..A.TG.

A.te AAATAT--CA TGCTATAGAA AATAGTACAA GGCCTTAGCT TAA-TTAAAG TTTCTGTTTT GCATGCAGGT GATGTGGGGT AATGTCCCGC AGGTCTTAAG

P.ii ..GGCC--.. .A..-....T .T.....T.. .......... ...-...... .......... ........A. ....C..... .G.A...T.. ..........

C.az G.CA.C--T. CA..-..... .......... A......... ...A...... .A.....C.. ..GGT...AA ....C..AT. ..AT.T...T ....T...T.

C.ko .GCCT.--TC .......... G.....GT.. .......... ...A...... .A.....C.. ..GGT...AA ....C...C. ..ATC....T ....T...C.

C.va ..TCC.--.C .AT....... .......T.. A......... ...G...... .GC....C.. ..GGT....A ....C..AT. ..AT.T...T ....T.....

L.ga ...ACC--.. ....-....T .....C.T.. A......... ...-...... .......... ........A. .......... ...AC..... ....T.....

A.po ..C.CCT-.. ....-....T ..C....T.. A......... ...-...... .......... ........A. .....A.... ...A...T.. ....T.....

B.pa GCGACC--.. G...-....T .....C.T.. A......... ...-...... CA.A..C... ...C...T.A ....CT..A. ....C..G.. ....T.....

L.la CTCCC.T-G- CA..-..A.T ..A...GT.. .......... ...-.C.... C.C...C... ........AG ........A. ...TC..... ...C......

P.oli ..CC..C-TG ...CGA...T ..CG.CGT.. ...T...... ...-...... .G........ ........T. ........A. ..CAG..... .A........

P.ste ..CC..T-T- ....GG...T ..C..C.A.. ...T...... ...-...... .G....C... ........AA ........A. .G........ .A......-.

C.lu ..CCC.C-GG CA..-....T .---..GTT. .......... ...-...... ..C...C... .........G ....C.A.A. ..CAA.TT.. ..........

B.my .T.A.CTTTG ....-..A.C .TA..CCG.. .......... ...-...... CACT..C... .......A.A .....C..A. .G.CCT.G.. ...CT.....

A.te CCGGCTAGTT TAACAAAAAC CCTTGATTTC GGCTTAAGAA TTTGTGGTTC GAACCCACAG CCGCCTA-TA GACAGATGCT CGCTGGTTTG GGCGCTTAGC

P.ii GA........ ...T...... .......... ....C..... C......... A.CT...... ..C....-.. .........C .......... ..........

C.az G......... .......... A......... ....C..... C........A A.TT...... ..A....-.. ..T....... ..T....... ..........

C.ko GG........ .......... ATA....... ....C.TA.. A........A A.G....... ....TA.-.. .......... ..GA.TA.A. ..........

C.va G......... ...A.....T T..A...... ....C....G C........A A.GT..G... ..A....-.. .......... ...G...... ..........

L.ga G......... ...TG..... .......... ....C...C. C......... ..CT..G... ..C....-.. .......... .......... ..........

A.po GAC....... ...T...... .......... ....C...C. C......A.. ...A..G... ..T....-.. ...G.....C .......... ..........

B.pa G.A....... ...AC..... .T........ ....C..AC. C......... A.CT..G... ......G-.. .........C .......... ..........

L.la G......... ...G...... .......... ....C...C. C.......C. A.G...G... ..CT...-.. .......... ......C... ..........

P.oli G.A.T....C ...G...... AT........ ....C..A.. .........T A..T..G..A .......-.. ..T....... ......A... ..........

P.ste GT..T....C ...G...... AT........ ....C..A.. C........T A..T..G..A ....T..A.. ..T....... ......A... ..........

C.lu G.AA...... ...TT..... T......... ....C...C. C........T A.....G... T..T...-.. ..T...G..C .......... ..........

B.my G.AT...... ...AT..... .......... ....C..AT. .........A A..T..G... .T.....-.. ..T......C .......... ..T.......

A.te TGTTAACTAA GATTTTGCAG GGTCGAGGCC TGCCTGTCTA GAAGACGCTA GTAAAAGGT- ATTACAGTGC CTTGTCGAGG CACAATTTTG AGCTCACACC

P.ii .......... .....C.... .A....AA.. .......... .......... ......CT-- ......C... .......... ..G..G..C. G.T.A.AC..

C.az .......... .......... .A........ .....A.... .G....A.G. ...TCGCA-- -..G..TC.. .......... .GA.GGC.C. G.T.A.AG..

C.ko .......... ..G..C..G. .T.AA..T.. C......... .G.AG.A.G. ....CGCA-- -.....TC.. T.....A... .GA.GGC... G.T.A.AT..

C.va .......... .......... .A........ .......... .G..G.A.G. ....CGCA-- -..G..TC.. .......... .GA.GGC.C. G.T.AGATT.

L.ga .......... .......... .......... .......... .....TA... ......A.-- ......C... .......... ..G..G..C. T.T.A.AC..

A.po .......... .....C.... .A........ ....C..... ......A... ......AA-- ......C... .......... ..G..G..C. G.T.A.AC..

B.pa .......... ..G..C.... .......... .......... .G...G.... ......CA-- ......CCA. .......... TGG....... G.T.A.AC..

L.la .......... ..C....... .A........ .......T.. .....TT... ......AC-- .....GC... T.....A... ..G.G...CA G.T.AGAG..

P.oli .......... ..AG...T.. .A........ .A...A.... .....TA... ......TTCT ..A..GC... ......A... ..G...CG.. G.T.A.AG..

P.ste .......... .......T.. .A..A..... .T...A.... .....TA... ......C..T ..A...C... ......A... ..G....G.. G.T...AC..

C.lu .......... ..G..C.... .......A.. .......... .G..GT.... ......AT-- .....CC... .........A ..G.G...CA G.T.AGAC..

B.my .......... ..G..C.... ......A... .....A.... .....TA... ......AA-- ......CG.. GA......T. .CGG....C. ..T.A.A...

A.te TCAAGCGTCT TGA-GCTCTG GGGTGTTATC ATGTCAGATT GCAAATCTGA AGAAGCGGGC TAGCCGCCCG TCGGGGCTTT AGAAATTGGT GTAGTGGAAG

P.ii C.G...A... .A.-...... ......A... ....T..... ........A. ......A.AT G.ATA.T.T. C...A..... .....G.... ....A.....

C.az C.G..T.... .T.-.TC... ........A. .....G.... .......C.. ...G.....T ..ATA..... C.A....... ....GG.... ..........

C.ko C.G..T.CT. CT.-.TC... .A......A. .C...G.... .......C.. ...T....C. ..AAGCT... C......... ....GG.... ...A..C.-.

C.va C.G..T.... CT.-.TC... ........A. .C..TG.... .......C.. ...G.....T C.A.A..... C.A....... ....GG.... ..........

L.ga C.G..T.... ...-...T.. ......G... .C........ .......... ...T..A..T GGATA...T. C...A..... .....G.... ..........

A.po C.G..T.... .A.-....C. ......G... .C........ .......... .....TA..T G.AGA...T. C......... .....G.... ..........

B.pa C.....A... .A.-....C. .......... .C.C...... .........G ...G.TA... G..TA...T. C......... ...G...A.. ....C.....

L.la CTG..TC... .A.-...... A.....G... .C..T..... ........A. .....TA... G.AGA...TA C......... ...G.G.A.. ...T...G..

P.oli C.GC.TA... ...-..C... ........C- .C..T..... ........A. .....TA... ...T....TA C......... ..G..G.... ....A.....

P.ste C.GC.TA... .A.A..C.C. .CAGACG..- TA...T.C.. CTTT.GA.TT GC..T.TAA. AT.TAA.A.C C.A......- ..G..G.... ....A.....

C.lu CTG...AC.. .T.-...T.. ........C. .C..T..... ........T. ......A..T G.AAG..... C...A..... .....G.A.. ...C......

B.my ..G..TT... .A.-.TCT.. ......C... .CA.T...C. ........AG ......A..A G.AGAT..T. C..A.A.... ...G.G.A.. ...TA.....

A.te CACTAAGAGT TTTGATTTCT TCAGGCTGGG TTCGAATCCC ATGTTTCTAA GTTCTCGTAG TTGAAT-ACA ACAGTGGTTT TTCGAGTCAC AAGTCTTGGT

P.ii .......... ......C... .....T.... ...A...... ..C....... ...GCT.... ......-... ..G....... ...AGC.... T.....C...

C.az ...A.G.... ......C..C .....T.A.. .........T G.CC.....T ...ACT.... ......-... ..G....... ...ATTC..T TG....C...

C.ko ...A.G...C ....GCC..C .A..CT.A.. ...A.G...T .GTC.....T ...ACT.C.. ......-.G. .TG....A.C ...ACT.... TG.C.CC...

C.va ...A.G.... ......C..C .....TCA.. .........T C.CC...... ...ACT.... ......-... ..G....... ...ACT.... TG....C...

L.ga .......... ......C... ..G..T.... .....G.... ..T....... ...ACT.... ......C... ..G....... ...AGC.... T.....C...

A.po .......... ......C... .....T.... .....G.... ..T....... ...ACT.... ......T... .......... ...ATTC... C...T.C...

B.pa .......... ......C... .....T.... ......C... ..A....... ...GC..... ......A... ..G....... ...A.TC... T.........

L.la .......... ......C... .....T.... ...A.G.... ..C....... ...GAT.... .....AT... ..G......C ...AGCC... T.C.......

P.oli ...A...... ......C... .....T.... .....GC... T.C....... ...T.T.... ......G... ..GAC..... ...A..C.GT G....C....

P.ste ...G...... ......C... .....T.... .....TC... T.C....... .....T.... ......A... ..G....... ...A..C..T T....C....

C.lu .......... ......C... .....T.... .....GC... ..C....... ...GAT.... .....AG... .......... .....A..G. C.........

B.my .......... ......C... .....T.... ...A.CC... ..C....... ...ACT.... ......T... ..G....... ....TA.... T.....A...

A.te TAGACCCCAA GCGAGAACTG TCTTTCTAGT AT-AATT-CA GTATAGGTGC CTTCCAAGCA CCCCGTCTTG GTTTAAACCC AAGGAAAGAT A-GCAGTCAT

P.ii ..A.AT..G. ..AG.....A .......... ..T....-.. .....A.... .......... ..TG.C.... ...AG..T.. .......... .-....A...

C.az ..A.AT..GG ..AGA....A ....CT.... ..T...A-A. ....GA.... ......C... .A.G...... ...C...T.. ....G..... G-....A...

C.ko ..A.AT..GG ..AGA..T.A ....CT.... ..T...A-T. ....TA.... .......... .A.G...... ...C..CT.. ....G..... .-.T..AT..

C.va ..A.AT..GG ..AGA....A ....CT.... ..T..CA-.. .....A.... ......C... .A.G.C.... ...A...T.. ...AG..... G-....G...

L.ga ..A..T..G. ..AGA....A ...C...... ..C..C.-.. .....A.... .......... ...G...... ...A...... .....G.... .-....A...

A.po .T..GT..GG ..AGA....A .......... .CC..C.-T. .....A.... .......... ..TG...... ...A..GT.. .......... .-....A...

B.pa ....AT..TG ...G.....A ...C....A. ..C..-.TT. .....C...A .......T.. ...A...... ...AG..... .....GG... .-..G.A...

L.la ..A.GT.... ..AT...... .....T.... ..A.GG---. A...GA...A .......T.. .TTGAC.C.. ...-G..T.. .G.A.....C .-.T..A...

P.oli ..A.GT..TG ..AGA....A .......... ..T..GCG.. .....A...A .......T.. ...G...... ...A..TT.. .......... .-.T..G...

P.ste ..A.GT..TG ..AG...T.- ....C..... ..C..A.CT. .....A...A .......T.. ..TG...... ...A...T.. ....G..... .A.T..GT..

C.lu C.A.TT.... .TATT...C. .....T.... .CA.C..--. .........G .......C.. ..TG..T... ...AG.G... ..AA...... .-....ATG.

B.my GGA.GA..T. ..AG.....A .T.C.T.... ..T..-.--- ...C.TA..A .......T.. TAAA...CC. .C.A-.C... GG...G.A.. .-....G...

A.te AGTTTAATGC AAAACCCTAG ATTGTGATTC TGAAAATAGA CGTTAATCCC G-TCTTGACT GCAGAAGCTG TGCCTGAACC TTA-AAGGAA CACTTTGATA

P.ii ......G.AA ..--.AT... .......... ....G.C... ......A... .-.....T.C ..C...A... ........TT ...-.....G ..........

C.az ......GCTA ...-TAT... .......... ....G.C... G.....AAT. C-.....T.C ..C..GA.C. .......TTA A----...G. ..........

C.ko ......G.TA ...-T.T... G.....GCG. ....GTC..G G.....AAT. C-....AT.. A....GA.C. .......TTA A----...G. ..........

C.va ......GCTA ...-TAT... .......... ....G.C... T.....AA.. C-.....C.A ..C..CA.C. .......TTA A----....T T.........

L.ga ........CA ...-TGT... .......... ....G.C... G.....AG.. C-.....T.C ..C....... ........TT ...-.....C ..........

A.po .......AAA ....TAT... .......... .A..G.C... .....GA... CC.....T.C ..C.G.A... ........T. C..-G-..GC ..........

B.pa .......CTA ...-.AT... ......G... .AG.G.C..G T.....AAT. C-C....T.A ..C.G..... ....C..G.. .G.A.G...C ..........

L.la .......-T. .....A.... .......... .AGC..C.AG .......... .-.T...T.C A.C.GG.... ....C...T. -C.--....C ..........

P.oli .......ATA ...-.AT... .......... .A....C... G...G.AG.. C-.....C.C A.C.G..... .........T .A.--....C ..........

P.ste .....T.ACA .....AT... .......... .AG.....AG G.....AAT. C-CT..TC.C A.C.G..... ........TT .A.--...GC ..........

C.lu .......CTT .....GT... .......... .A.....G.. ....G.AA.. C-C.C.ATT. ..C.G..T.. ........A. AC.--...GC ..........

B.my .......CTA ...-TAT... .......... .A..G.A... .....GA.T. .-.....C.C ..C.G..AC. ....C..GTG AG.T.G.... ...C......

A.te GAGTGAACTA TGGGGGTTAA AATCCCCCCA GCTTCCGTTG GTGTGGCAGA GCC---CGGC TA-TGCAAAG GCCCTAAAAA CCTTGTACGG AAGTTCAAAC

P.ii ........C. .......... .G........ .T...T.... .A........ ...---...T G.A....G.. .T.....G.T ...CCC.... .G......GT

C.az ....AT.TC. ..C....... .GC...G..G .TC..T.... .A........ ...---...T ..A-..GC.. .T.....G.T ....C...AA .G.......T

C.ko ....AT.T.. ..C....... .GC...G..G .TC..T.... .A........ ...---...T ..A-..GC.. .T.....G.C ....C...AA .G......GT

C.va ....AT.GC. ..C....... .G....G..G .TC..T.C.. .A........ ...---...T ..A-...C.. .T.....G.T ....C...AA .G.......T

L.ga .....TCT.. .......... .G.......G .....T.... .A........ ...---...T ..-....G.. .G.....G.T ...CCG...A .G.......T

A.po .....T.AA. ..A....... .G....T... ...C.T.... .A........ ...---...T ..-....G.. .G.....G.T ...CC...AA .G.......T

B.pa AG...T..C. C......... .T....T... T..C.A...A .C........ ...CGGTA.T ..A....G.. .G.....G.T ...CCC..AA .G......CT

L.la ......CT.. ..T....... .G........ ..CCTT.... .G........ ...---...T G.T....... .A.....G.C ....CA.... .G.A.....T

P.oli .......T.. .......... .......... A..C.T...A .G........ ...---.... ..A......A .A.....GCC .T..CC..A. .G......GT

P.ste .......TC. .......... .G........ A..C.T.... .G........ ...---.... ..A......A .A.....GCC .T..CC..A. .G......GT

C.lu .....G.AC. ....T....G .......... A..C.T.... .A........ ...---.... A.T-...GGA .A.....G.T ..CCCG...A ........GT

B.my .G...CTT.. C......... .C....T..G ...C.A.C.. .G........ .TA-GG.AAT -.A...G.G. .G.....G.C ....AG..AA GG.......T

A.te CCTCCCCCCA ACTGCTTTCA AAGGATAATA GTTCATCCGT TGGTCTTAGG AACCAAAGAC TCTTGGCGCA ACTCCAAGTG GAAGCTCACT AAGAAGCTAA

P.ii ...T...... ......CCT. .......... .......... .......... .......... .......... .A.......A .T.....G.. ..........

C.az ...TT.TT.. .......CT. .......... .........C .......... .....G.... .......... .A.......A .T....-GT. ........G.

C.ko ...TT.TT.. .......CT. ........C. .........C .......... .....G.... .......... .A.......A .T.....G.. ........G.

C.va ...TT.T... .......CT. .......... .......... .......... .....G.... .......... .A.......A .T.....GT. ........G.

L.ga ...T...... ......CCT. .......... ........A. .......... .......... ......T... .M.......A .C.....G.. ..........

A.po ...TT..... ......CCT. .......... .......... .......... .......... ......T... .........A .C.....G.A ..........

B.pa ...TT.T.T. ...A..CCTG ........C. ..CT...... .......... .......A.A ...C..T... .A...G..CA .T..T..G.. ........-.

L.la ...T...... ......GCT. T......... .C......A. C......... ....G..A.. ...C..T... ..C......A .T.....G.. ........T.

P.oli ....T..TT. ......CCT. ........C. .C........ .......... .......A.. ......T... .A.......A .C........ ..........

P.ste ....T..TT. ......CCT. ........C. .C........ .......... .......A.. ......T... .A.......A .C.....G.. ..........

C.lu ...T...... .......CT. ........C. .C........ .......... .......A.. C.....T... .A.......A .C.....G.A G.........

B.my ..CTT..... ......CCTG ........C. .C.A....RC .......... .....GGA.. ......T... .A...G..CA .T.....GT. ........T.

A.te CAAGTAACAG CGCCAGACTT TTGATCTGGA GACCGGTGAC CACCGCCCAC CCTTAGTGAA GTAAGGTAAG CTAAT--TAA GCTGTCGGGC CCATACCCCG

P.ii AG....CA.. .A....C... ..A....... A.TT....GT T.A....... ....T.C... .C...A.... ....A--G.. ...C.T.... ....G....A

C.az A......A.. .......... ..A....... A.TT....TT T.A..T.... ....T.C..G .C...A.... T....GTA.. -..T.T.... .........A

C.ko A......A.. .......... ..A....... A.AT.....T T.A..A.... ....T.C..G .C...A.... T...CACA.. -..T.T.... .........A

C.va A......G.. .......... ..A....... A.TT....TT T.A....... ....T.C..G .....A.... T...AGGA.. A..T.T.... .........A

L.ga AT....CA.. ......C... ..A....... ..TT....GT T.A.A..... ....T.C... .....A.... ....G--C.. ...C.T.... ....G....A

A.po AT....CA.. ......C... ..A....... ..TT....G. ..A.A..... ....T.C... .....A.... ....C--... ...C.T.... ....G....A

B.pa A.T...-A.. ......C... ..A....... ..TT...... T.A.A..... ....T.C.G. ....AA.... .....--C.. ...CCT.... .........A

L.la ATGT..GA.. .A....C... ..A....... ..TT....GA T.A.-..... ....T.C.GG .C...A.... ....A--A.. ...C...... ..........

P.oli T....CC... ..TT..C... ..A.G..AA. A.TT....C. T.A.AA.... ........G. ....A..C.. ....A--... ...C.T.... .........A

P.ste T.....CA.. ..TT..C... ..A.G..AA. ...T....C. T.A.AA.... ......C... ....A..C.. ....G--C.. ...C.T.... .........A

C.lu AC...T.A.. ......C... ..A....... ..TT....G. T.A....... ....T.C... .......... T...A--... A..A...... ..........

B.my A.....CA.. ......C... ..A.G....T ..T-...... T.A.....G. ......C... .....A.... .....--C.. .....T.... .........A

A.te AATATGCAGG TTGAAATCGT -GCCCTTACT AGCGACCGTA GCTTAAT--T AAAGCATGAC CCTGAAGATG TTAAGATGAA CT-GTAAAAA G-TTTCGGTA

P.ii ..A...T... .....CC.C. -..TT..G.. ...A...... .......--. .......A.. A......... .......A.. .C-T..G... .-......G.

C.az ..C....G.. A.A..T..CC C..T...G.C ...CG..... ......C--. .......A.. A......... .......... ..GT-..... .-..C...A.

C.ko .......G.. A.A.....CC -..T...G.C ...CG..... ......A--. .......... A......... ........T. .CGT-..... .-..C...AG

C.va G......G.. C.A..C..CC T..T.....C ...A...... ......A--. .......A.. A......... .......... ..GT-..... .-C.C...G.

L.ga .......... .GAG.CC.C. -..TT..... ...A.T.... ......A--. .......A.. A......... ...G....G. .CATCGT..G .-.C...AGG

A.po ..C....... .GA...C.C. -..TT..... ...CCT.... ......G--G .......... A......... ........G. ..CT.GT..G .-.C...AG.

B.pa G......C.. .GA..CC.CC -..TT..... ...C..T... ......A-G- .......A.. G......... ........GG ..CTC.C..G .-CC....GG

L.la .CC.C...T. .GAG.CC.A. -..T...G.C G..C..T... ......A--G .......... A......... ...G....G. ..GTCG...- .-.C....A.

P.oli .C....T... ..C.....C. -T..T..... ...T.A.... .......-T- .......A.. A......... ........GG .CCT-.G... .-CCC..TAG

P.ste .CC...T... ..A.....C. -...T..G.. ...T.A.... .......-T- .......A.. A......... ........GG .CCT-.G... .-CCC...GG

C.lu ..C.....A. .GAG..C.CC -......... ...AG.T... ......ACGA .......A.. A......... ........GG .CCTC.CCTC A-CCC.....

B.my .TC..KYM.. .GA..CC.C. -..TT..... G.TT.T.... ......A-GG .......... A......... .C....CAGG .CCTC.C... .ACC...AC.

A.te GCACAAAGAG TAGTTTAAA- -TAAAACATT GGCTTTGGGA GCCAAAAATG GGGGTTAGAC CCCCTTCTCT TTGAAAGAAA ATGGCAGAGT GGTTATG-TG

P.ii ......G... ........-- -..G..TTC. .......... ....GGGG.A ..A....... ..T....... .......... G........A .....C.-C.

C.az .....GGA.. .......G-- -..G..TTC. .........G ....G.GGCA ..A....A.. ..TTC...TC C......... G..T.....G ..C....-..

C.ko .....GGA.. .......G-- -..G..TTC. .........G ....G.GGCA ..A....A.. ..TTC...TC C......... G..T.....G ..C....-..

C.va .....GGA.. .......G-- -..G..TT.. .........G ....G.GGCA ..A....A.. ..TTC...TC C...G...G. G-.C.T.CAG C.C.GAA---

L.ga ......G... ........-- -..G..TCC. A......... .TT.GGGG.. ..A....A.T T.T....... .......... G..A.....C .......-..

A.po .....GG.G. ........-- -..G..TTC. A......... ..T.GTGG.A ..A...TA.. T.T.C....C .......... G........C .......-C.

B.pa ......G.G. ........TG TG.G..TCC. .......... ....GGGGCA ..A....A.. ..T......C .......... G......... ...AT..-..

L.la ......G... .......GGG CA.G..TCC. .......... ....GGGGC. ..A....A.T ..T.C....C .......... G......... ...C...-C.

P.oli .....G.A.A ........CG C..G..TCC. A......... .TT.GGGG.A ..A...GA.A T.T.C.T.T. C......... G........C .......-C.

P.ste .........A .......GTG C..G..TCC. A......... .TT.GGGG.A ..A...GA.A T.T.C.T... .......... G..A.....C .......-..

C.lu .....GG.G. .C..C...TT TA.G..TCC. A......... ..T.GGGG.A ..A...CA.. T.T......C C...G...G. GGCT.GC..C AA.G.A.ACT

B.my ......G.G. ........TG CG.G..TCC. A......... ..T.GGGG.A ..A....A.T A.T....C.. .......G.T G..A....CA .AA....-..

A.te GTCGACTTGA AATCGGCACA CGGG-GGTTC GATTCCCTCT TTTCTGG-GA GAGAGGCTTG -TAGCACTGG AGACTGCTAA TCTTC-ACGA CC-TGGGTTA

P.ii .CT....... ....A.TTT. T...-..... ......TC.C .......-.. ........C. -C.......A G......... .....-...C ..-CC....G

C.az ..T....... ....AA.TT. T.A.-..... ..C....C.C .....T.C.. .......C.. -C...G.... .......... .....-.T.T ..-CC....G

C.ko ..T....... ....AA.TT. T.A.-..... ..C....C.C .....T.-.. ........C. -C...G...A .......... .....-.T.T T.-CC....G

C.va ---....GCT ....TT..TG TACCC....G ...C..GGGC .CA..C.-A. ..A..TG.CA -G.TTGGCTA T.--..G.TG A...G-.AAT .A-GCTTA.G

L.ga .CT....... ....A.TTT. T...-..... A.C...T..C .....C.-.. ........C. -C.....G.A .......... .....-.T.. ..-.T....G

A.po .CT....... ....A..TT. T...-..... A.C...T..C .......-.. ........C. -C.....G.. G......... ..CC.-.... ..C.T....G

B.pa T......... .....A.CT. ....-..... A.....TC.C .....C.-.. ........C. -C.......A .......... .....-..A. .T-.T....G

L.la ..T....... ....TC.TT. TA..-..... ........TC .....C.-.. ....T..... -C.....CAA .......... ....TTG.AC ..-CC....G

P.oli A.T.G..... ..C.AATTG. T...-..... A.C...TC.C .....C.-.. ........G. -C....A... .A........ .T...-.T.. ..-.T....G

P.ste ..T.G..... ..C.AATTG. T...-..... A.C...TC.C .....C.-.. ........C. -C....A... GA........ .TCC.-.... ..-.T....G

C.lu .CTA.T..TC .TGACC.TAG TT..-ACCC. AGGG.---TC AC..CAA-.. A..T...AGA A.G.TCA..T G.T.GA..TG AAA..G.TAT A.-G...G.T

B.my ..T....... ....AATTA. T...-....A ..A......C ..C..C.-.. ........C. -C....A..A G......... .....-..C. .T-.T....G

A.te AAATCCAAGG CTCACTCG-- -----TCAGC GGTAGCTCAG -CTTAAGAGC ACCGGCTTTG TAACCCGGAG GCCGGCGGTT TAAA--TCCG CCCCGCTGAA

P.ii G.CC..GG.. ........-- -----G.GA. A......... A.C.-..... G....TC... ...G.....T .....G...G A...--C..C ...T.GC.C.

C.az ..TC..-G.. ...C....TA CCCCCG..A. T......... GGACT..... G....TC... ...G.....T .....A.... ...C--C..T .....T..CT

C.ko ..CC..-... ........-- -----G.... T......... GGACT..... GT...TC... ...G...A.T .T...A.... ...C--C..T ..T.....CT

C.va .GGGTT-C.A ...C.C.CTT TCTTGG..C. T......... GGA.T..... G.....C... ...G.....T .T...A.... ...C--C..T ...A....CT

L.ga G.CC...G.. .......A-- -----G.... A......... A.C.-..... G....TC... ...G.....T .....G.... A...--...C ........CC

A.po G.CC...... ........-- -----G.... .......... ATC.C..... G....TC... ...A.....T .....G.... A...--...C ........CT

B.pa G.CC....A. ........-- -----G.... .......... A.-.C..... G....TC... ...A.....C .....G.... AG..--...C ........CC

L.la G.....G... A.......-- -----G.... A......... A.-....... G.....C... ...G...... .T...G.... A...CCC..C ...TC...CT

P.oli G.C...TG.. ........-- -----G..TT A......... TG..-..... G..A.TC... ...A.T..CT .T..AG.... A...--...C T..T.T..CT

P.ste G.CC..C... ........-- -----G..CT A......... CG.CC..... C..A.TC... ...A.T.ACC .T...A.... A...--...C ...TA...CT

C.lu CG....CCTC ..TT....-- -----G.... A......... AA-CC..... G....TC... ...A...... .T...G..C. A.---TC..C ...TC...GG

B.my G.CC....A. ........CA -----G..T. .......... G.ACT..... GT....C... ...G...A.T .A...G..C. .GG.--A..C ..T..A..CC

A.te AGTGGCTTAG GTTAAAAC-- AAACCAGGGG CCTTCAAAGC CCCCATCGTG GGTGAAGCCC CCACAGCCTC TG------GG CGGGGTGGCT GAATGAT-AA

P.ii ..G....... .C.T..G.-- .......A.. .......... .T.A..T.G. ....G.A... ..T.....C. ..------.. .........C .....T.C.G

C.az .AG....... .C.CG.T.-- .......... .......... .....A..G. A.....AAA. T.C.....CT ..------.C .........C ..G..T.G.G

C.ko .AG....... .A.CG.C.-- .......... .......... ...T.A..G. A....GAAT. T.C.....CT ..------.C .........C .....C.T.G

C.va .AG....... .A.CG.C.-- .......... .......... ...T.A..G. A....GAGT. T.C.....CT ..------.A ........TC ..G..T.T.G

L.ga ..G....... .C..G...-- ......A... .......... ..TA....G. .....GAA.. ..C.....C. .A------.. ....A..... ..G..TCC..

A.po ..G....... .C.TG.G.-- .......... .......... ...A....GA ....GGATT. .TC.....C. .A------.. A...A..... ..G..TCC..

B.pa ..G....... .C.CG...-- ......A... .......... ..TA....G. ....G.AG.. ..T.....C. ..------.. .A........ ..G..T.AT.

L.la ..G....... .C.CG-.AC- .......... .......... ...A.C..A. ......AA.. ..C....... ..------.. .A........ ..GA.-GT..

P.oli ..G....... .A..GC.TTA .G....A... .......... ..TA.G..G. A.....AAT. T.C.....C. ..T-----.. TAA.AA.AG- .GC.-CGA--

P.ste ..G.A..... .C..GC.ATT .G....AC.. .......... .GT..G.... A.....AAT. T.T...T.C. ..------.. TA..A..... ..G.-TAA--

C.lu ..A.A..... T.A..T.AT- .G.A.GA... .......... ...AGGT.G. ....T.AAT. ..C....... ..------.A .A........ ..G..CAT..

B.my ..GC...... ....GC.T-- .....GA... .......... ..TA....AA ......A.T. .TT.....C. ..CCCCAC.. G......... .....TGA-.

A.te GCGGCGGATT GTAGCTCCGT ACACGGAGGT TTAATTCCTC TCGCCGCCAC AGGACGTAGC TAAA---AAG -ATAGCATCT CCCTTACACC GAGAAGTCAC

P.ii .......... ....TC.... .A........ .......... ..CA..T... .......... ...G---... T......... .........T ..........

C.az ....T..... .........C CG........ .....C.... C.C...T... .......... ...G---... A......... .........T ..........

C.ko .T........ .....C...C TG........ ....G..... C.C...T... .......... .G.G---... A......... .........T ..........

C.va ....T..G.. ........AC CA........ .........T CTC...T... ..AG...... ...GT--... A.....G... .........T A.........

L.ga .......... ....TC.... GA..A.T... ........C. .TC....... .......... ...G---... T......... .......... .....C....

A.po .......... ....TC.... GA..A..... ........C. .TC..TT... .......... ....---G.. A......... .......... ..........

B.pa ....T..... ....AC..A. GG........ .AG......T .TC.T.T... .......... ...G---... T......... .......... ..........

L.la ....T..... ....AC..A. .A..AA.... ....C....T .TC.T..... .ACC...... ....A--G-- -......C.. .........T ...GC...G.

P.oli A.CT.T.TC. A.G.GG.TAC .AT.CACC.C ....C..AG. CAT..TA.C. ...GT..... ...GCC-.GA CCC....C.. .A........ ......A..T

P.ste ....T..... ....AC..A. .G..A..... GC..GC.... .TCTTAT... ..AG...... ...GAT-.GA T.C....... .A........ ......A.GT

C.lu .T.CT..C.. ....TC..A. GA..A..... .G..A....T .TC.T.T... ...G....A. ...GA--... A....T.... .A........ ........C.

B.my ..T.T..G.. ....T...A. TG.TA..... .AG....... ..C..T.... .......... ...GACA.C. C.....G... .......... .........T

A.te CCGTGCAAAT CGGGTCGGCC TGA-

P.ii ..T......A .......... ...-

C.az .T......GC .A.....C.. ...-

C.ko .T......GC .A.....C.. ...-

C.va .T.......C .A.....C.. ...-

L.ga ......G... .......... ...-

A.po T.....G... ..A....... ...-

B.pa .........C .......... ...-

L.la ........C. ....C.A.GT ...-

P.oli ........G. ...A...C.. ...C

P.ste ........G. ...A...C.. ...-

C.lu .......... ....G..A.. ...-

B.my ....T...G. ...A...... ...-

**S1 Fig.** **Alignment of the sequences of 12RT dataset from mitogenomes of 13 species of flatfishes.**

Abbreviations of species names are shown in Table 1.
