# Supplementary material for: Concerted Evolution of Duplicate Control Regions in the Mitochondria of Species of the Flatfish Family Bothidae (Teleostei: Pleuronectiformes)
Source: PLoS One. 2015 Aug 3;10(8):e0134580. doi: 10.1371/journal.pone.0134580 (PMC4523187; doi:10.1371/journal.pone.0134580)
Supplement: S2 Fig — Abbreviations of species names are shown in Table 1. Numbers following species names represent the types of the CRs: CR1 or CR2. “1” represents the CR1, and “2” represents the CR2, respectively. (DOCX) [file pone.0134580.s002.docx]

A.te1 CCATTCACTT ACTTGGACCA TTCAAGTGCT A-CACTAACC TCAGGGATGG CTAGGGTAG- -GCCCCCCAT AATATATACA T-TCCATAGT AGTCA-TGCG -CT---AGTA AATTAAACCC

A.te2 .......... .......... .......... .-........ .......... .........- -......... .......... .-........ .....-.... -..---.... ..........

L.ga1 .....A.A.. .TC.TA.... ....G.GA.. TA..TCC.AT CT.A.AGATA ...AAAGCC- -AAA.AT.TC .TG-ATCTT. .T.T...GAA .C...-A.TA CAC---.A.. G..A.G.TAA

L.ga2 .....A.A.. .TC.TA.... ....G.GA.. TA..TCC.AT CT.A.AGATA ...AAAGCC- -AAA.AT.TC .TG-ATCTT. .T.T...GAA .C...-A.TA CAC---.A.. G..A.G.TAA

L.la1 A.....CTGC GT..TC.AAT A.A.C.CA.A GCA.GA.C.G A.CATTTGTA TG.TTACTT- ----AATAC. ..ACCCCTTG AC.TA.CT.C TTATGAAA.T CAAGAACA.. .CACT.CTT.

L.la2 A.....CTGC GT..TC.AAT A.A.C.CA.A GCA.GA.C.G A.CATTTGTA TG.TTACTT- ----AATAC. ..ACCCCTTG AC.TA.CT.C TTATGAAA.T CAAGAACA.. .CACT.CTT.

P.ii1 .......A.. .TC.C..... ....G.GA.. TG..TA..AT CT.A..GAAC GCTAAAGTA- -AAA.AT.CC .TC-.T.GT. CCCT.....G .T...-A.TT CAA---.AAT TG.G..GTTA

P.ii2 .......A.. .TC.C..... ....G.GA.. TG..TA..AT CT.A..GAAC GCTAAAGTA- -AAA.AT.CC .TC-.T.GT. CCCT.....G .T...-A.TT CAA---.AAT TG.G..GTTA

A.po2 ....CGCT.. .TA..A.G.. ....G.G..- -AA.GAC.A. CT.A..TA.A ....A-G.T- -AAA...GG. .T--ATC.G. CCCTT....A .T...-A.-A C.A---...C T..A....T.

B.my2 .AGAGGCAC. .TG.AT.AT. AG..TTG.T. TATTT...A. AATTTAG.CT TCCATTA.TC ----AAAG-. .T--AT.GG. CTAGAGACAG ...AGT--GC AAA--T.TA. .--.TCT.AT

B.pa1 ....AGGT.. TA..A..A.. A...G.AC.. TA..T.CT.- .T.A.A.G.. .A..AACCTG -AT.AAG.TC .CG.AGAG.. .GGTAGC... --CT.G.A.A CAA---C.CT .T....TA..

C.az2 .......A.. .TA.CA.G.. .......TTA GA...GTTAT GT.AT...C. GA.AA.GCA- -CAT...TGA .G--.GG... CACA.....G ...AGGAA.A -.A---C... GGCA..G...

C.ko2 .....AT... ..C.TA.GA. A...G.AATG .TAC....AT GA.......A .AGTA.GCA- -TT.ATT.GA .GCC.GA.G. CAAT.....A .A.G.G...A T.G---CAC. .GAG..T...

C.va2 ....G..A.. .TA.CA.... AA.....TT. GG..TA.TTA .G.ACTGCTC TC.AA.G.A- -ATAAG---C GGG-.GCGT. CAAT.....G TT...-A.T- ------.AAC TCAAGG.TAA

C.lu2 TT..AGC... .TG.ATTATC AC..TA.AG. GAATT...A. A-TACAGGTC .C.ATT..TC ----.TA--C .T--AT.GT. CAAATTA..A TT.A.T.TAC AA.--TCAAC .--...TAAT

P.ste ..TAATCTAG TAA.AT.G.. C...TTCAAC .A..T.TTTA A.TAA...A. -ACTA.A.CC -CAAT...CC .C.GACCTT. CA.TAC.G.A .C.ATGG.GC CA--GCC.AG .T....GA..

P.oli A..AAACA.G GTG.CA.A.. ....TA.A.C .G.TA..TA- A.TAAATATA TACAAAA.CC ----AAA.C. .T--A.GGT. .ACAATA.AG .A.T.AA.AC TAG--TC.A. .C..T.CA..

A.te1 A-ACATTC-T TGATAAGTT- --AAACATAT ATACCAAGTC CCCACATCTC TGAAGT-TTC CCCAATAATG CACAGCAAGA ACCGACCTAC ACTA-CTATT TCTTAATGAT AAC-GGTTAT

A.te2 .-......-. .........- --........ .......... .......... ......-... .......... .......... .......... ....-..... .......... ...-......

L.ga1 .-...GGA-C GA.C.CC.AA AC.T.T...C CA.G..CCCA A.-.T...-T GA-.A.A..A GAA..A.T-. .....T.... .......... ....-..... ........C. ...-......

L.ga2 .-...GGA-C GA.C.CC.AA AC.T.T...C CA.G..CCCA A.-.T...-T GA-.A.A..A GAA..A.T-. .....T.... .......... ....-..... ........C. ...-......

L.la1 TA....CG-. CTTG..CAAA ACGG.T.C.C CA.GT.C--- T...T..... ..TGTA-... AAAT...G.. .......... .......A.. GTAT--G... A......ACC C..-.T....

L.la2 TA....CG-. CTTG..CAAA ACGG.T.C.C CA.GT.C--- T...T..... ..TGTA-... AAAT...G.. .......... .......A.. GTAT--G... A......ACC C..-.T....

P.ii1 .-...GCT-C AATC.TTAAA TC.T.T...C CA.GT.CCAT G.-.T.CTAT GAT...CC.A -ATT.AT--. .......... .......... GAGT--.... ........CC ...-......

P.ii2 .-...GCT-C AATC.TCAAA TC.T.T...C CA.GT.CCAT G.-.T.CTAT GAT...CC.A -ATT.AT--. .......... .......... GAGT--.... ........CC ...-......

A.po2 .-....AA-A AATC.T...G AC.CCT.C.C CA.GT.CA.G A.-.T...-T GA-.AC-.AA .ATG.GTT-. .....T.... .......... GG..AT.... A.......C. ...-......

B.my2 GA..TCAA-A CAT-.TA.AC CA.GT.TCCA GC...T---- ..-----GCA GCTCA---AA GAAGT.GC.- --...T.... .......AT- --CT-.G.A. AAAG.G.ATA .T.-.T....

B.pa1 -A..TCAA-A GTCCGC.AA- -AC.CTC..C CA.GT.---- T.A...A.AA ATG.A.-G.. AAA.GG.T.. .....T.... .....G.A.A CTAC--...A ....T...CA ...-.T....

C.az2 .-....AT-. AA.C...AGG GA.CC..CCA CGTTT.G... G.AT.CATG. AACCTC-CAA TGTTG---.. AT...T.... .....G.... .AGT-AC... C......... ...-......

C.ko2 CT.GG.AT-C AAT..G.AC- --.CT..G.C .GT--..TC. G.A.TGGGG. .A.CAC-.GA .A.T.CCT.. AG...T.... .....G.... .AGC-AC... ........CA TGG-CC....

C.va2 C-T..ACA-A AA..C.AAGA ACTGG..CGC CCGG..G.AA ATG..CC.AT AAGG.CC... .AAT.ATT.. AG...T.... .....G.... .AGC-AC..A A.....G.CA TGG-CC....

C.lu2 CT..T.AA-C CAT-.TA.AC CA.GTAGAGA CC.T.T---- .TT.T.AACT ..TGA---CA TGAGT.GG-- --..ATT... G..C...A.T GACT-.C.C. C.AGGG..TA ..GA.A..C.

P.ste GA...CAA-C ACTC.TCAGT CG.GTT...C CA.GAC---- T.A.A..... -TTCAC--AT .AG.TCCC.A TGT..T.... G..T...A.. CGGT--G... A......... ...-......

P.oli GA...CAA-C CTTC.TA.GT CA.GTT...C CA.GAC---- T.A.AC.TCT GTCGA.--C. .AA.T.CCCA TG...T.... G..T...AT. -AGT-TG... A.......CC ...-......

A.te1 TGATGGTCAA GGTCAATAAT T-GTGGGGGT TTCACTTAGT G-CACTATTC CTGGCATTTG GTTCCTATTT CAGGTACACT -CATTTATTT G-TCCCTCAT TCTTTCATCG ACGCTTGCAT

A.te2 .......... .......... .-........ .......... .-........ .......... .......... .......... -......... .-........ .......... ..........

L.ga1 .........G ..A...C... C-........ ......C... .-A.T..... .......... .......C.. .....C..T. -A...G..A. TA.T....GC A.......T. .....G....

L.ga2 .........G ..A...C... C-........ ......C... .-A.T..... .......... .......C.. .....C..T. -A...G..A. TA.T....GC A.......T. .....G....

L.la1 .......... ..A...G... .-........ CG...CCC.. .-A....... .......... .C.....CC. ....GT..T. GGT..AGA.- -AAT..A..C A......... ..A.......

L.la2 .......... ..A...G... .-........ CG...CCC.. .-A....... .......... .C.....CC. ....GT..T. GGT..AGA.- -AAT..A..C A......... ..A.......

P.ii1 .........G ..A..G.... .-........ .....C.... .-A....... .......... .......... ....GC..TG -AGCC...A. CA.T..G... A.G....... ..........

P.ii2 .........G ..A..G.... .-........ .....C.... .-A....... .......... .......... ....GC..TG -AGCC...A. CA.T..G... A.G....... ..........

A.po2 .......... ..A...A... C-........ .A...CCC.. .-A.T..... .......... .......C.. ....GC..T. GA...G..G. TA........ A.......T. ..........

B.my2 .........G ..A...CT.. C-........ AA...AC... .-A....... .......C.. .......... ....GC..TG -G.C.G-AAG TAGT..C... CGA.CG.CT. ..........

B.pa1 .........G .........C .-........ .....ACTA. .-A.T..... .......... .......C.. ....G..-A. G.C..GGA.A CG....A... A...C...T. ..........

C.az2 .......... ..A...G.CC .-........ ....A..C-. AG..T..... .......... .......C.. ....A...TC T..CCG..AC AA....-... A.G.C...T. ..A.......

C.ko2 .........G ..A..GA.CC .-........ ....AC.CC. .A..T..... .......... .......C.. ....A...T. A.-.AG.GA. .A....G... C..A.G.C.. ..........

C.va2 .........G ..A...A..A .-........ ....A.ACT. A-..T..... .......... .......C.. ....A...TA -AGAA.GCA. TAAA..C... A.G.C...T. ..........

C.lu2 ...A...G.G ..A...CT.. A-..A.AATA .A..AACG.. .-A....... .......... .......C.. ..A.G...T. -ACCAG-.GA TAATA..... A......CT. .GA..GA...

P.st2 ...A...G.G ..A...A... .-........ .....C.C.. .-ATT..... .......... .......C.. ....GC..TG -A..CG.-G. CACT..C..C A......... ......A...

P.ol2 ...A...G.G ..A...A... .-........ .....A.... .-A....... .......... .......C.. ....GC..TG -ACC.G..A. TA.T.....C A......... ..A...A...

A.te1 -AAGTTAATG GTGTTAAC-C AT-CTCTTCG TTACCCCCCA AGCCGAGCGT TCACTCCACA GGGCCATTTG GTTCTCTTTT TT--ATTCTC CTTTCAATTG GCAT-TTCAG AGTGCAGCGC

A.te2 -......... ........-. ..-....... .......... .......... .......... .......... .......... ..--...... .......... ....-..... ..........

L.ga1 -......... T.CGA...-. ..-.C..... ......A..T .....G.... ......T.AT ....-..... .......... ..GGT..--. ......C..T A...-..... .......A..

L.ga2 -......... T.CGA...-. ..-.C..... ......A..T .....G.... ......T.AT ....-..... .......... ..GGT..--. ......C..T A...-..... .......A..

L.la1 -......... ...G....-. ..-.C..... ......A..T .......... .......... ...G..GC.. ....-..... ..AGG..-.. .......C.T ....-..... ..........

L.la2 -......... ...G....-. ..-.C..... ......A..T .......... .......... ...G..GC.. ....-..... ..AGG..-.. .......C.T ....-..... ..........

P.ii1 -......... ...GC...-. ..-.C..... ......A..T .....G.... ........TC ....-..C.. ....CT.... .-CTC..T.. ......C... ....-....C ......AG..

P.ii2 -......... ...GC...-. ..-.C..... ......A..T .....G.... ........TC ....-..C.. ....CT.... .-CTC..T.. ......C... ....-....C ......AG..

A.po2 -......... T.CGA...-. ..-.A..... ......A..T .....G.... ......T.AG ....-..... ....CT.... ..TTC..A.. ......CC.T ....-C...C .......GAA

B.my2 -TG....... ...GAG..CA T.ATAA.AAC AC....AA.T ...A.G..AC ...T..T.-. ...A...A.. ..AG...... ..TAT..T.. ......-CAA A...-G...- ....T---AA

B.pa1 -......... ...GA...-. ..-....... ......A..T .......... .......... .C.-...C.. ..A.--C... ..TAT.A-.. .....CTC.T A...-..... .......G..

C.az2 -......... ...AC...-. ..-....... .......... .....G..A. ...T..T.A. .TA.-..C.. ....CTC... ..CTC..T.. ......CCCT ....-GGGCC T.G-TT.TT.

C.ko2 -......... ...AA...-. ..-.A..... ......A... .....G..A. ......T.A. ....-..-.. ...A.T.... A.TAT..T.A A.C...GGCA A...-CCAT. CA--T...TA

C.va2 -......... ........-. ..-.A..... ......A... .....G...C ......T.A. ....-..... ....CT.... ..CTC..A.. ......C..T A.GCGCC..T GA.CATAA.T

C.lu2 CTG.....C. ...G....CA CGT--A.GG. AC....A... .....G...C .......... ...G..A..A ....C..... A.TTT..TC. T.....GC.T ....-....C ....T---TA

P.ste -......... T..A...TA. ..A.GAC... ......AG.. .....G.... ........GC ...T-.AGG. .......... ..TTT-.--. ......C... ....-..... ......T.CA

P.oli -......... T..G..TTA. ..A.GAC... ......AG.. .....G.... ........-G C..GT.AGG. .......... --TTT..TC. T.....TC.. ....-..... ......T.TA

A.te1 GTCTAT---G TTATATTAAG GTTGATCATT TTTGG---TG CCAGGCCAC- ATGTAATGTA ATGCT-TTTA AACATTCT-A TAAAGAATTG CAATTTATTA TCTCACGGGC ATAAGTGTAT

A.te2 ......---. .......... .......... .....---.. .........- .......... .....-.... ........-. .......... .......... .......... ..........

L.ga1 ....-A---C GAC...C... ....TA.... ..C-T----T G.CT.....A ..AAGTGTC. T.T..-.AAG G.T.A...T. ...GA.-... ....AAGAG. .A...T.... ....CA-.G.

L.ga2 ....-A---C GAC...C... ....TA.... ..C-T----T G.CT.....A ..AAGTGTC. T.T..-.AAG G.T.A...T. ...GA.-... ....AAGAG. .A...T.... ....CA-.G.

L.la1 ......---. A.....C... .....G...C ..CCT----T GTTT.TA..A .A.AT.ATG. .....-AGAG G....GTCT. ...GA.-CCA ..C.AA.GG. .A...T.... ....CA.C..

L.la2 ......---. A.....C... .....G...C ..CCT----T GTTT.TA..A .A.AT.ATG. .....-AGAG G....GTCT. ...GA.-CCA ..C.AA.GG. .A...T.... ....CA.C..

P.ii1 A.TA-A---A AACA.A.... ....TC.... .CCCC----T G.CC.....A .GAATG.TA. CGCT.-AGAG G.T.....GC ...GA.-... ....AAT.A. .A...A.... ...TTAC...

P.ii2 A.TA-A---A AACA.A.... ....TC.... .CCCC----T G.CC.....A .GAATG.TA. CGCT.-AGAG G.T.....GC ...GA.-... ....AAT.A. .A...A.... ...TTAC...

A.po2 .C.CGA---T GAC.GAC... .........C AC.-T----T G..TAGA.-A .C.GGTATG. ...TC-AGG. ......GAT. C..GAGGC.. ...CC.T.A. .A...G.T.. .....A..G.

B.my2 .CTC.CA--A AATA..GG.A AG...A...G A.-------- -GCCC.GGGA CACATG.TG. ...AATAAA. G....AGCCG -G.CA.-CCA ..T.AA.C.. .A...G.T.. ...C-GT.G.

B.pa1 ...A.----A GCTAGCC... ....T....G .CCCT----T G..CCG..AC .A.ATGGTG. ...A.TAAA. G.....GA-. ...GA.-.G. ..T.C....T .A...G.T.. .....CAC.C

C.az2 AAT..----A CCCA--C... ..C.GA...A C..T.ATTGT ..CT..T..T .AT..G..-. ...T.-AAA. G...A...-. C..GAGCAGA ..TA.A.A.. .T...A..A. .....GC..-

C.ko2 AGG..C---. ...A--C... ..C.GA...A .CCC.--TG. T.TA.....- ..AA.G..-. ...T.-AAAG G..TC.AA-T A..GTGT..A ..TAA.TC.. AGCTT...A. .....AC.CA

C.va2 AAGA-G---A GACA.GGGC. .ACA.--... C.GAC----T GACC.A-G.G ..AATGGTG. ...G.-GAA. G......AA. C..GA.-.AA ..CAAAT.G. .A...G.T.. ...TACA.C.

C.lu2 AATC.GA--A AA.ATCA... ....GA...A AC..TCGTAC .ACTCAGGTA .ATGTGGTG. ...A.G.AA. GT....ACTG ...G.G-C.. ..T.AATC.. .A...G.T.. ...C-A....

P.ste .C.A.CCGAA ACG.T.A... .G...G.... ...CT----T G..CT.GC.G TACAT.GTAT CCATGTAACG ..AC..TATT AG.....CCA ..T.AATAG. .A...A.T.. .....GA.G.

P.oli .C.G..AG-A AATAT.C... ..G..A.... .CCTTG---- .GT.CAAGTA .ATAGTCTC. ...TAA..A. -T.T..A.CT A..GA.-.AA ..T.AA.AG. .A...A.T.. ...--A.GG.

A.te1 ATTCCACCCC CTCC

A.te2 .......... ....

L.ga1 CC..TC.T.T ...A

L.ga2 CC..TC.T.T ...A

L.la1 .C.G.TA.AT .CTA

L.la2 .C.G.TA.AT .CTA

P.ii1 .C.T.TTAA. ...A

P.ii2 .C.T.TTAA. ...A

A.po2 ...TTCTTGT ...A

B.my2 GG..TGAA-- ----

B.pa1 .CAG.TTTGA .-TA

C.az2 -----T.GTT TATT

C.ko2 TA.T.TT.TT TCTT

C.va2 ..CGTTT... ..TT

C.lu2 TC.TT.TT-- ----

P.ste GC.TG----- ----

P.oli GG..TTGT-- ----

**S2 Fig. Alignment of the core region sequences of the CRs from mitogenomes of 13 species of flatfishes.**

Abbreviations of species names are shown in Table 1. Numbers following species names represent the type of the CRs: “1” represents the CR1, and “2” represents the CR2, respectively.
