# Supplementary material for: Concerted Evolution of Duplicate Control Regions in the Mitochondria of Species of the Flatfish Family Bothidae (Teleostei: Pleuronectiformes)
Source: PLoS One. 2015 Aug 3;10(8):e0134580. doi: 10.1371/journal.pone.0134580 (PMC4523187; doi:10.1371/journal.pone.0134580)
Supplement: S1 Table — (DOCX) [file pone.0134580.s003.docx]

**S1 Table Primers used for fragment amplifications in five flatfish mitogenomes**

|  | Forward  primer | Sequences (5′-3′) |  | Reverse  primer | Sequences (5′-3′) |
| --- | --- | --- | --- | --- | --- |
| *Psettina iijimae* | Z15 | ATTAAAGCATAACHCTGAAGATGTTAAGAT |  | F-16S | GACAAGTGATTRCGCTACCTT |
|  | Z-16S | CTYGTACCTTTTGCATCATG |  | F13413 | TAGCTGCTACTCGGATTTGCACCAAGAGT |
|  | Z-ND1 | GGBTAYATRCARHTNCGNAARGG |  | F-ND1-380 | GCRTAYTTNGARTTNGANGCYCANCC |
|  | Z-ATP6 | ACHTTYACNCCHACHACNCARCTNTC |  | H-ND1 | TGYATDARYTGRTCRTADCGRAANCG |
|  | Z13347 | AAGGATAACAGCTCATCCGTTGGTCTTAGG |  | F-COI | GGRTARTCNGARYANCGNCGDGGYAT |
|  | Z-Ile | AAGGRHYACTTTGATAGAG |  | F-Cys | WCAYRTNRGATTGCAAAYC |
|  | R-COI | GGTCAACAAATCATAAAGATATTGG |  | F-ND2 | TGDGHRATNGANGARTADGCNAG |
|  | Z10818 | TTYGAAGCAGCCGCMTGATACTGACAYTT |  | F11089 | TTTAACCAAGACCRGGTGATTGGAAGTC |
|  | Z13347 | AAGGATAACAGCTCATCCGTTGGTCTTAGG |  | F-ATP6 | AGRTGNCCNGCNGTBARRTTNGCNGT |
|  | Z-Cytb | TNACHTGRATYGGNGSNATRCCNGT |  | F-ND5 | ATRATNGCRTCYTTDGARWARAANCC |
|  | Z-Gln | CAARACTCYTNGTGCTYCC |  | F-Cytb | TGNCCRATRAKVAYRWADGGRTSTTC |
|  | Z-ND5 | GCNATRCTNTTYYTNTGYTCNGGNTC |  | F-Phe | GTTATGCTTTRNWTAAGCTAC |
|  |  |  |  |  |  |
| *Lophonectes gallus* | Z15 | ATTAAAGCATAACHCTGAAGATGTTAAGAT |  | F2753 | TAGATAGAAACTGACCTGGATTACTCCGGT |
|  | Z2733 | ATCCAGGTCAGTTTCTATC |  | F13413 | TAGCTGCTACTCGGATTTGCACCAAGAGT |
|  | Z-Ile | AAGGRHYACTTTGATAGAG |  | F6746 | GCGGTGGATTGTAGACCCATARACAGAGGT |
|  | GMP-COI-R | TCATTCTCCACGACACCT |  | F-ND2 | TGDGHRATNGANGARTADGCNAG |
|  | Z-ATP6 | ACHTTYACNCCHACHACNCARCTNTC |  | F-ND4 | CCYATGTGVCYNACDGADGAGTADGC |
|  | Z10818 | TTYGAAGCAGCCGCMTGATACTGACAYTT |  | F-ND5 | ATRATNGCRTCYTTDGARWARAANCC |
|  | Z-Leu | CTTAGGWACCARARACTCTTGG |  | F-COI | GGRTARTCNGARYANCGNCGDGGYAT |
|  | Z-ND5 | GCNATRCTNTTYYTNTGYTCNGGNTC |  | GMP-ATP-F | GCTACTTCCAGCAGTGTTA |
|  | Z-Cytb | TNACHTGRATYGGNGSNATRCCNGT |  | F-Gln | GGRAGCACNARGAGTYTTG |
|  | Z-Gln | CAARACTCYTNGTGCTYCC |  | F-Phe | GTTATGCTTTRNWTAAGCTAC |
|  |  |  |  |  |  |
| *Arnoglossus tenuis* | Z15 | ATTAAAGCATAACHCTGAAGATGTTAAGAT |  | F2671 | AGATAGAAACTGACCTGGAT |
|  | 16SAR | CGCCTGTTTATCAAAAACAT |  | F-ND1-380 | GCRTAYTTNGARTTNGANGCYCANCC |
|  | Z2733 | ATCCAGGTCAGTTTCTATC |  | H-ND1 | TGYATDARYTGRTCRTADCGRAANCG |
|  | Z-Ile | AAGGRHYACTTTGATAGAG |  | F11089 | TTTAACCAAGACCRGGTGATTGGAAGTC |
|  | Z10818 | TTYGAAGCAGCCGCMTGATACTGACAYTT |  | F13413 | TAGCTGCTACTCGGATTTGCACCAAGAGT |
|  | Z13347 | AAGGATAACAGCTCATCCGTTGGTCTTAGG |  | F95 | GACAGTAAAGTCAGGACCAAGCCTTTGTGC |
|  | Z-COI | TNACHTTYTTYCCNCARCAYTTC |  | F-COI | GGRTARTCNGARYANCGNCGDGGYAT |
|  | Z-ND5 | GCNATRCTNTTYYTNTGYTCNGGNTC |  | H-Cytb | CCNARNARDGANCCRAARTTTCA |
|  | R-Cytb | CAYGCYAAYRGHGCHTCHTTYTTYTT |  | F-Cytb | TGNCCRATRAKVAYRWADGGRTSTTC |
|  | Z-Gln | CAARACTCYTNGTGCTYCC |  | F-ND6 | ATYTAYYTDGGNGGDATRYTVGTDGT |
|  |  |  |  |  |  |
| *Chascanopsetta lugubris* | Z15 | ATTAAAGCATAACHCTGAAGATGTTAAGAT |  | F2671 | AGATAGAAACTGACCTGGAT |
|  | H-ND1 | GTHTCNGGNTTYAAYGTHGARTAYGC |  | F-COI | GGRTARTCNGARYANCGNCGDGGYAT |
|  | 16SAR | CGCCTGTTTATCAAAAACAT |  | F5196 | CTAAATGGTTGGGGTATGG |
|  | DK-Cytb-R | ACTTGTTACTGCCCTTGAG |  | F11089 | TTTAACCAAGACCRGGTGATTGGAAGTC |
|  | Z-COI | TNACHTTYTTYCCNCARCAYTTC |  | F13413 | TAGCTGCTACTCGGATTTGCACCAAGAGT |
|  | Z10818 | TTYGAAGCAGCCGCMTGATACTGACAYTT |  | F-Gln | GGRAGCACNARGAGTYTTG |
|  | Z13347 | AAGGATAACAGCTCATCCGTTGGTCTTAGG |  | H-Cytb | CCNARNARDGANCCRAARTTTCA |
|  | Z-Gln | CAARACTCYTNGTGCTYCC |  | F49 | GGCCCATCTTAACATCTTC |
|  | L17114 | RCGCCCAAAGCTAGDATTC |  | DK-16S-F | TTTCAGCKTTYCCTYGCGGTACT |
|  | DK-Z-CR | TCTATATCAGGTGGATAC |  | F-Phe | GTTATGCTTTRNWTAAGCTAC |
|  |  |  |  |  |  |
| *Crossorhombus valderostratus* | Z15 | ATTAAAGCATAACHCTGAAGATGTTAAGAT |  | F-ND1-380 | GCRTAYTTNGARTTNGANGCYCANCC |
|  | Z2733 | ATCCAGGTCAGTTTCTATC |  | F-COI | GGRTARTCNGARYANCGNCGDGGYAT |
|  | 16SAR | CGCCTGTTTATCAAAAACAT |  | 16SBR | CCGGTCTGAACTCAGATCACGT |
|  | Z-Ile | ATCAACGAACCAAGTTATCCTA |  | F11089 | TTTAACCAAGACCRGGTGATTGGAAGTC |
|  | Z-COI | TNACHTTYTTYCCNCARCAYTTC |  | F13413 | TAGCTGCTACTCGGATTTGCACCAAGAGT |
|  | Z10818 | TTYGAAGCAGCCGCMTGATACTGACAYTT |  | F-Ile | CTCTATCAAAGTRDYCCTT |
|  | Z13347 | AAGGATAACAGCTCATCCGTTGGTCTTAGG |  | F-ATP6 | AGRTGNCCNGCNGTBARRTTNGCNGT |
|  | Z-ATP6 | ACHTTYACNCCHACHACNCARCTNTC |  | F-ND5 | ATRATNGCRTCYTTDGARWARAANCC |
|  | Z-ND5 | GCNATRCTNTTYYTNTGYTCNGGNTC |  | F-Cytb | TGNCCRATRAKVAYRWADGGRTSTTC |
|  | L-ND5-450 | TTYCARCTHTTYATHGGBTGRGA |  | H-ND5 | CAYCANCCRATNARDAGRAANGA |
|  | Z-Gln | CAARACTCYTNGTGCTYCC |  | F-Gln | GGRAGCACNARGAGTYTTG |
|  | R-Cytb | CAYGCYAAYRGHGCHTCHTTYTTYTT |  | F-Phe | GTTATGCTTTRNWTAAGCTAC |
