# Supplementary material for: Concerted Evolution of Duplicate Control Regions in the Mitochondria of Species of the Flatfish Family Bothidae (Teleostei: Pleuronectiformes)
Source: PLoS One. 2015 Aug 3;10(8):e0134580. doi: 10.1371/journal.pone.0134580 (PMC4523187; doi:10.1371/journal.pone.0134580)

| **S2 Table**. **Features and gene maps of the mitogenomes of five species of Bothidae**  ***Arnoglossus tenuis*** | | | | | | | | | | | | | | | |  |
| --- | --- | --- | --- | --- | --- | --- | --- | --- | --- | --- | --- | --- | --- | --- | --- | --- |
| Gene | Position | | | | Length(bp) | | AA | | Anticondon | | Start/Stop condon | | Intergenic region* | | Strand |  |
|  | From | | To | |  |  |  |  |  |  |  |  |  |  |  |  |
| *tRNA-Phe* (*F*) | 1 | | 68 | | 68 | |  | | GAA | |  | | 0 | | H |  |
| *12S* | 69 | | 1017 | | 949 | |  | |  | |  | | 0 | | H |  |
| *tRNA-Val*(*V*) | 1018 | | 1087 | | 70 | |  | | TAC | |  | | 0 | | H |  |
| *16S* | 1088 | | 2789 | | 1702 | |  | |  | |  | | 0 | | H |  |
| *tRNA-Leu* (*L1*) | 2790 | | 2862 | | 73 | |  | | TAA | |  | | 1 | | H |  |
| *ND1* | 2864 | | 3841 | | 978 | | 325 | |  | | ATG/AGA | | 3 | | H |  |
| *tRNA-Ile* (*I*) | 3845 | | 3916 | | 72 | |  | | GAT | |  | | 6 | | H |  |
| *tRNA-Met* (*M*) | 3923 | | 3991 | | 69 | |  | | CAT | |  | | 0 | | H |  |
| *ND2* | 3992 | | 5041 | | 1050 | | 349 | |  | | ATG/TAG | | -2 | | H |  |
| *tRNA-Trp* (*W*) | 5040 | | 5109 | | 70 | |  | | TCA | |  | | 5 | | H |  |
| *tRNA-Asn* (*A*) | 5115 | | 5187 | | 73 | |  | | GTT | |  | | -7 | | L |  |
| OL | 5181 | | 5230 | | 50 | |  | |  | |  | | 8 | | H |  |
| *COI* | 5239 | | 6786 | | 1548 | | 515 | |  | | GTG/TAG | | 66 | | H |  |
| *COII* | 6853 | | 7543 | | 691 | | 230 | |  | | ATG/T | | 0 | | H |  |
| *tRNA-Lys* (*K*) | 7544 | | 7616 | | 73 | |  | | TTT | |  | | 1 | | H |  |
| *ATP8* | 7618 | | 7785 | | 168 | | 55 | |  | | ATG/TAA | | -10 | | H |  |
| *ATP6* | 7776 | | 8459 | | 684 | | 227 | |  | | ATG/TAA | | -1 | | H |  |
| *COIII* | 8459 | | 9244 | | 786 | | 261 | |  | | ATG/TAG | | -1 | | H |  |
| *tRNA-Gly* (*G*) | 9244 | | 9313 | | 70 | |  | | TCC | |  | | 0 | | H |  |
| *ND3* | 9314 | | 9664 | | 351 | | 116 | |  | | ATG/TAG | | -2 | | H |  |
| *tRNA-Arg* (*R*) | 9663 | | 9731 | | 69 | |  | | TCG | |  | | 0 | | H |  |
| *ND4L* | 9732 | | 10028 | | 297 | | 98 | |  | | ATG/TAA | | -7 | | H |  |
| *ND4* | 10022 | | 11402 | | 1381 | | 460 | |  | | ATG/T | | 0 | | H |  |
| *tRNA-His* (*H*) | 11403 | | 11472 | | 70 | |  | | GTG | |  | | 0 | | H |  |
| *tRNA-Ser* (*S2*) | 11473 | | 11539 | | 67 | |  | | GCT | |  | | 5 | | H |  |
| *tRNA-Leu* (*L2*) | 11545 | | 11617 | | 73 | |  | | TAG | |  | | 0 | | H |  |
| *ND5* | 11618 | | 13450 | | 1833 | | 610 | |  | | ATG/TAA | | 9 | | H |  |
| *CytB* | 13460 | | 14599 | | 1140 | | 379 | |  | | ATG/TAA | | 1 | | H |  |
| *tRNA-Thr* (*T*) | 14601 | | 14672 | | 72 | |  | | TGT | |  | | 0 | | H |  |
| CR2 | 14673 | | 15502 | | 830 | |  | |  | |  | | 0 | | H |  |
| *tRNA-Gln* (*Q*) | 15503 | | 15573 | | 71 | |  | | TTG | |  | | 4 | | L |  |
| *tRNA-Ala* (*A*) | 15578 | | 15646 | | 69 | |  | | TGC | |  | | 1 | | L |  |
| *tRNA-Cys* (*C*) | 15648 | | 15713 | | 66 | |  | | GCA | |  | | 0 | | L |  |
| *tRNA-Tyr* (*Y*) | 15714 | | 15783 | | 70 | |  | | GTA | |  | | 0 | | L |  |
| *tRNA-Ser* (*S1*) | 15784 | | 15854 | | 71 | |  | | TGA | |  | | 2 | | L |  |
| *tRNA-Asp* (*D*) | 15857 | | 15926 | | 70 | |  | | GTC | |  | | 4 | | H |  |
| *ND6* | 15931 | | 16452 | | 522 | | 173 | |  | | ATG/TAG | | 0 | | L |  |
| *tRNA-Glu* (*E*) | 16453 | | 16520 | | 68 | |  | | TTC | |  | | 1 | | L |  |
| *tRNA-Pro* (*F*) | 16522 | | 16590 | | 69 | |  | | TGG | |  | | 0 | | L |  |
| CR1 | 16591 | | 17556 | | 966 | |  | |  | |  | | 0 | | H |  |
| ***Arnoglossus tenuis***  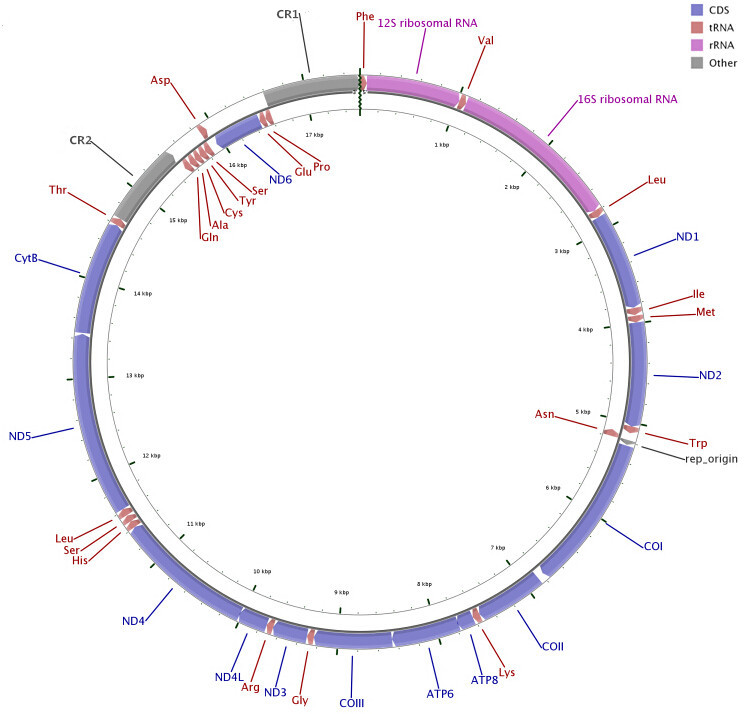  ***Chascanopsetta lugubris*** | | | | | | | | | | | | | | | | |
| Gene | | Position | | | | Length(bp) | | AA | | Anticondon | | Start/Stop condon | | Intergenic region* | | Strand |
|  |  | From | | To | |  |  |  |  |  |  |  |  |  |  |  |
| *tRNA-Phe* (*F*) | | 1 | | 71 | | 71 | |  | | GAA | |  | | 0 | | H |
| *12S* | | 72 | | 1032 | | 961 | |  | |  | |  | | 0 | | H |
| *tRNA-Val*(*V*) | | 1033 | | 1104 | | 72 | |  | | TAC | |  | | 0 | | H |
| *16S* | | 1105 | | 2808 | | 1704 | |  | |  | |  | | 0 | | H |
| *tRNA-Leu* (*L1*) | | 2809 | | 2881 | | 73 | |  | | TAA | |  | | 1 | | H |
| *ND1* | | 2883 | | 3863 | | 981 | | 326 | |  | | GTG/TAA | | 3 | | H |
| *tRNA-Ile* (*I*) | | 3867 | | 3937 | | 71 | |  | | GAT | |  | | 16 | | H |
| *tRNA-Met* (*M*) | | 3954 | | 4022 | | 69 | |  | | CAT | |  | | 0 | | H |
| *ND2* | | 4023 | | 5072 | | 1050 | | 349 | |  | | GTG/TAG | | -2 | | H |
| *tRNA-Trp* (*W*) | | 5071 | | 5141 | | 71 | |  | | TCA | |  | | 7 | | H |
| *tRNA-Asn* (*A*) | | 5149 | | 5221 | | 73 | |  | | GTT | |  | | -7 | | L |
| OL | | 5215 | | 5261 | | 47 | |  | |  | |  | | 8 | | H |
| *COI* | | 5270 | | 6835 | | 1566 | | 521 | |  | | GTG/TAA | | 70 | | H |
| *COII* | | 6906 | | 7596 | | 691 | | 230 | |  | | GTG/T | | 0 | | H |
| *tRNA-Lys* (*K*) | | 7597 | | 7669 | | 73 | |  | | TTT | |  | | 1 | | H |
| *ATP8* | | 7671 | | 7838 | | 168 | | 55 | |  | | ATG/TAA | | -10 | | H |
| *ATP6* | | 7829 | | 8512 | | 684 | | 227 | |  | | ATG/TAA | | -1 | | H |
| *COIII* | | 8512 | | 9297 | | 786 | | 261 | |  | | ATG/TAG | | -1 | | H |
| *tRNA-Gly* (*G*) | | 9297 | | 9366 | | 70 | |  | | TCC | |  | | 0 | | H |
| *ND3* | | 9367 | | 9717 | | 351 | | 116 | |  | | ATG/TAG | | -2 | | H |
| *tRNA-Arg* (*R*) | | 9716 | | 9784 | | 69 | |  | | TCG | |  | | 1 | | H |
| *ND4L* | | 9786 | | 10082 | | 297 | | 98 | |  | | ATG/TAA | | -7 | | H |
| *ND4* | | 10076 | | 11456 | | 1381 | | 460 | |  | | ATG/T | | 0 | | H |
| *tRNA-His* (*H*) | | 11457 | | 11526 | | 70 | |  | | GTG | |  | | 0 | | H |
| *tRNA-Ser* (*S2*) | | 11527 | | 11593 | | 67 | |  | | GCT | |  | | 3 | | H |
| *tRNA-Leu* (*L2*) | | 11597 | | 11669 | | 73 | |  | | TAG | |  | | 0 | | H |
| *ND5* | | 11670 | | 13514 | | 1845 | | 614 | |  | | ATG/AGA | | 19 | | H |
| *CytB* | | 13534 | | 14673 | | 1140 | | 379 | |  | | ATG/TAG | | 6 | | H |
| *tRNA-Thr* (*T*) | | 14680 | | 14750 | | 71 | |  | | TGT | |  | | 0 | | H |
| CR2 | | 14751 | | 15605 | | 855 | |  | |  | |  | | 0 | | H |
| *tRNA-Gln* (*Q*) | | 15606 | | 15676 | | 71 | |  | | TTG | |  | | 9 | | L |
| *tRNA-Ala* (*A*) | | 15686 | | 15754 | | 69 | |  | | TGC | |  | | 5 | | L |
| *tRNA-Cys* (*C*) | | 15760 | | 15825 | | 66 | |  | | GCA | |  | | -1 | | L |
| *tRNA-Tyr* (*Y*) | | 15825 | | 15895 | | 71 | |  | | GTA | |  | | 15 | | L |
| *tRNA-Ser* (*S1*) | | 15911 | | 15981 | | 71 | |  | | TGA | |  | | 0 | | L |
| *tRNA-Asp* (*D*) | | 15982 | | 16050 | | 69 | |  | | GTC | |  | | -2 | | H |
| *ND6* | | 16049 | | 16570 | | 522 | | 173 | |  | | GTG/TAA | | 0 | | L |
| *tRNA-Glu* (*E*) | | 16571 | | 16639 | | 69 | |  | | TTC | |  | | 30 | | L |
| *tRNA-Pro* (*F*) | | 16670 | | 16740 | | 71 | |  | | TGG | |  | | 0 | | L |
| NC | | 16741 | | 17251 | | 511 | |  | |  | |  | | 0 | | H |

|  | | | | | | | | |
| --- | --- | --- | --- | --- | --- | --- | --- | --- |
| ***Chascanopsetta lugubris***  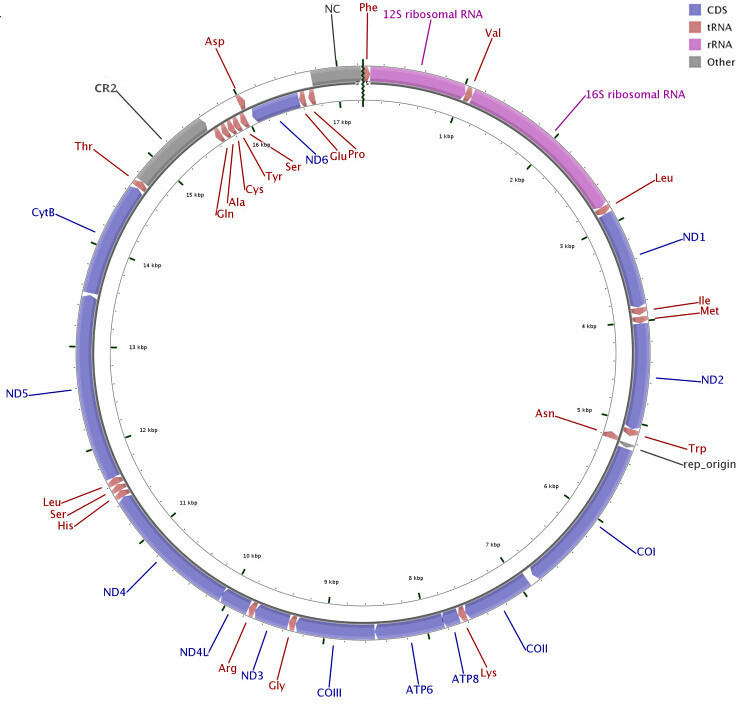  ***Crossorhombus valderostratus*** | | | | | | | | |
| Gene | Position | | Length(bp) | AA | Anticondon | Start/Stop condon | Intergenic region* | Strand |
|  | From | To |  |  |  |  |  |  |
| *tRNA-Phe* (*F*) | 1 | 68 | 68 |  | GAA |  | 0 | H |
| *12S* | 69 | 1029 | 961 |  |  |  | 0 | H |
| *tRNA-Val*(*V*) | 1030 | 1101 | 72 |  | TAC |  | 0 | H |
| *16S* | 1102 | 2823 | 1722 |  |  |  | 0 | H |
| *tRNA-Leu* (*L1*) | 2824 | 2896 | 73 |  | TAA |  | 0 | H |
| *ND1* | 2897 | 3874 | 978 | 325 |  | ATG/TAA | 3 | H |
| *tRNA-Ile* (*I*) | 3878 | 3946 | 69 |  | GAT |  | 26 | H |
| *tRNA-Met* (*M*) | 3973 | 4044 | 72 |  | CAT |  | 0 | H |
| *ND2* | 4045 | 5094 | 1050 | 349 |  | ATG/TAA | -2 | H |
| *tRNA-Trp* (*W*) | 5093 | 5162 | 70 |  | TCA |  | 6 | H |
| *tRNA-Asn* (*A*) | 5169 | 5241 | 73 |  | GTT |  | 12 | L |
| *COI* | 5254 | 6804 | 1551 | 516 |  | GTG/TAA | 76 | H |
| *COII* | 6881 | 7571 | 691 | 230 |  | ATG/T | 0 | H |
| *tRNA-Lys* (*K*) | 7572 | 7644 | 73 |  | TTT |  | 1 | H |
| *ATP8* | 7646 | 7813 | 168 | 55 |  | ATG/TAA | -10 | H |
| *ATP6* | 7804 | 8487 | 684 | 227 |  | ATG/TAA | -1 | H |
| *COIII* | 8487 | 9272 | 786 | 261 |  | ATG/TAA | -1 | H |
| *tRNA-Gly* (*G*) | 9272 | 9342 | 71 |  | TCC |  | 0 | H |
| *ND3* | 9343 | 9693 | 351 | 116 |  | ATG/TAG | -2 | H |
| *tRNA-Arg* (*R*) | 9692 | 9760 | 69 |  | TCG |  | 0 | H |
| *ND4L* | 9761 | 10057 | 297 | 98 |  | ATG/TAA | -7 | H |
| *ND4* | 10051 | 11434 | 1382 | 461 |  | ATG/T | 0 | H |
| *tRNA-His* (*H*) | 11435 | 11503 | 69 |  | GTG |  | 0 | H |
| *tRNA-Ser* (*S2*) | 11504 | 11569 | 66 |  | GCT |  | 8 | H |
| *tRNA-Leu* (*L2*) | 11578 | 11650 | 73 |  | TAG |  | 0 | H |
| *ND5* | 11651 | 13489 | 1839 | 612 |  | ATG/TAA | 7 | H |
| *CytB* | 13497 | 14637 | 1141 | 380 |  | ATG/T | 0 | H |
| *tRNA-Thr* (*T*) | 14638 | 14710 | 73 |  | TGT |  | 37 | H |
| *tRNA-Asp* (*D*) | 14748 | 14815 | 68 |  | GTC |  | 0 | H |
| CR2 | 14816 | 15519 | 704 |  |  |  | 0 | H |
| *tRNA-Gln* (*Q*) | 15520 | 15590 | 71 |  | TTG |  | 5 | L |
| *tRNA-Ala* (*A*) | 15596 | 15665 | 70 |  | TGC |  | 6 | L |
| *tRNA-Cys* (*C*) | 15672 | 15737 | 66 |  | GCA |  | 0 | L |
| *tRNA-Tyr* (*Y*) | 15738 | 15808 | 71 |  | GTA |  | 3 | L |
| *tRNA-Ser* (*S1*) | 15812 | 15882 | 71 |  | TGA |  | 36 | L |
| *ND6* | 15919 | 16440 | 522 | 173 |  | ATG/TAA | 0 | L |
| *tRNA-Glu* (*E*) | 16441 | 16508 | 68 |  | TTC |  | 4 | L |
| *tRNA-Pro* (*F*) | 16513 | 16580 | 68 |  | TGG |  | 0 | L |
| NC | 16581 | 16790 | 210 |  |  |  | 0 | H |

| ***Crossorhombus valderostratus***  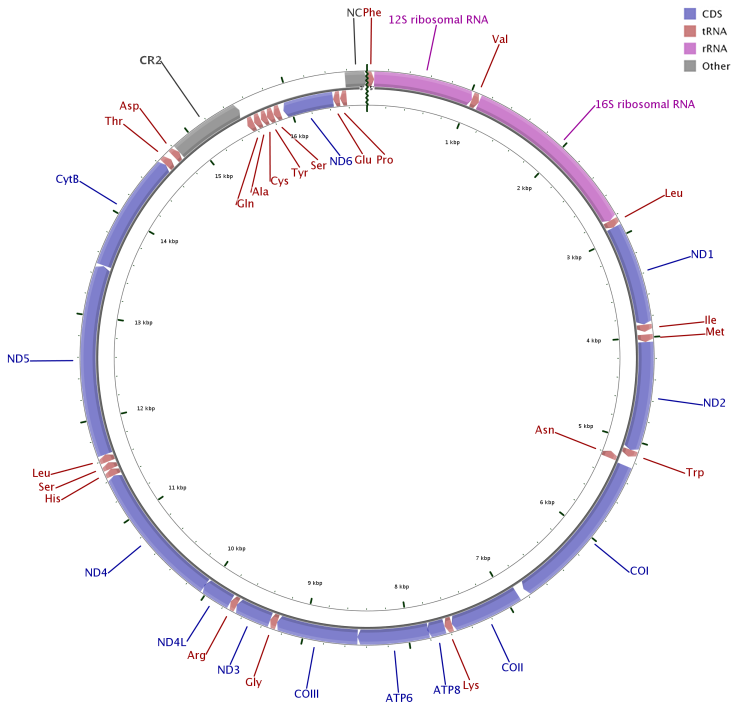  ***Lophonectes gallus*** | | | | | | | | | | | | | | | | |  |  |  |
| --- | --- | --- | --- | --- | --- | --- | --- | --- | --- | --- | --- | --- | --- | --- | --- | --- | --- | --- | --- |
| Gene | | Position | | | Length(bp) | | AA | | Anticondon | | Start/Stop condon | | Intergenic region* | | Strand | |  |  |  |
|  |  | From | To | |  |  |  |  |  |  |  |  |  |  |  |  |  |  |  |
| *tRNA-Phe* (*F*) | | | 1 | 69 | | 69 | |  | | GAA | |  | | 0 | | H | |  |  |
| *12S* | | | 70 | 1465 | | 1396 | |  | |  | |  | | 0 | | H | |  |  |
| *tRNA-Val*(*V*) | | | 1466 | 1536 | | 71 | |  | | TAC | |  | | 0 | | H | |  |  |
| *16S* | | | 1537 | 3247 | | 1711 | |  | |  | |  | | 0 | | H | |  |  |
| *tRNA-Leu* (*L1*) | | | 3248 | 3320 | | 73 | |  | | TAA | |  | | 1 | | H | |  |  |
| *ND1* | | | 3322 | 4302 | | 981 | | 326 | |  | | ATG/TAG | | 6 | | H | |  |  |
| *tRNA-Ile* (*I*) | | | 4309 | 4380 | | 72 | |  | | GAT | |  | | 5 | | H | |  |  |
| *tRNA-Met* (*M*) | | | 4386 | 4454 | | 69 | |  | | CAT | |  | | 0 | | H | |  |  |
| *ND2* | | | 4455 | 5504 | | 1050 | | 349 | |  | | ATG/TAG | | -2 | | H | |  |  |
| *tRNA-Trp* (*W*) | | | 5503 | 5572 | | 70 | |  | | TCA | |  | | -3 | | H | |  |  |
| *tRNA-Asn* (*A*) | | | 5570 | 5642 | | 73 | |  | | GTT | |  | | -6 | | L | |  |  |
| OL | | | 5637 | 5685 | | 49 | |  | |  | |  | | 11 | | L | |  |  |
| *COI* | | | 5697 | 7244 | | 1548 | | 515 | |  | | GTG/TAA | | 88 | | H | |  |  |
| *COII* | | | 7333 | 8023 | | 691 | | 230 | |  | | ATG/T | | 0 | | H | |  |  |
| *tRNA-Lys* (*K*) | | | 8024 | 8096 | | 73 | |  | | TTT | |  | | 1 | | H | |  |  |
| *ATP8* | | | 8098 | 8265 | | 168 | | 55 | |  | | ATG/TAA | | -10 | | H | |  |  |
| *ATP6* | | | 8256 | 8939 | | 684 | | 227 | |  | | ATG/TAA | | -1 | | H | |  |  |
| *COIII* | | | 8939 | 9724 | | 786 | | 261 | |  | | ATG/TAA | | -1 | | H | |  |  |
| *tRNA-Gly* (*G*) | | | 9724 | 9794 | | 71 | |  | | TCC | |  | | 0 | | H | |  |  |
| *ND3* | | | 9795 | 10145 | | 351 | | 116 | |  | | ATG/TAG | | -2 | | H | |  |  |
| *tRNA-Arg* (*R*) | | | 10144 | 10212 | | 69 | |  | | TCG | |  | | 0 | | H | |  |  |
| *ND4L* | | | 10213 | 10509 | | 297 | | 98 | |  | | ATG/TAA | | -7 | | H | |  |  |
| *ND4* | | | 10503 | 11883 | | 1381 | | 460 | |  | | ATG/T | | 0 | | H | |  |  |
| *tRNA-His* (*H*) | | | 11884 | 11952 | | 69 | |  | | GTG | |  | | 0 | | H | |  |  |
| *tRNA-Ser* (*S2*) | | | 11953 | 12019 | | 67 | |  | | GCT | |  | | 7 | | H | |  |  |
| *tRNA-Leu* (*L2*) | | | 12027 | 12099 | | 73 | |  | | TAG | |  | | 0 | | H | |  |  |
| *ND5* | | | 12100 | 13938 | | 1839 | | 612 | |  | | ATG/TAG | | 29 | | H | |  |  |
| *CytB* | | | 13968 | 15108 | | 1141 | | 380 | |  | | ATG/T | | 0 | | H | |  |  |
| *tRNA-Thr* (*T*) | | | 15109 | 15180 | | 72 | |  | | TGT | |  | | 0 | | H | |  |  |
| CR2 | | | 15181 | 16016 | | 836 | |  | |  | |  | | 0 | | H | |  |  |
| *tRNA-Gln* (*Q*) | | | 16017 | 16087 | | 71 | |  | | TTG | |  | | 7 | | L | |  |  |
| *tRNA-Ala* (*A*) | | | 16095 | 16163 | | 69 | |  | | TGC | |  | | 4 | | L | |  |  |
| *tRNA-Cys* (*C*) | | | 16168 | 16233 | | 66 | |  | | GCA | |  | | 0 | | L | |  |  |
| *tRNA-Tyr* (*Y*) | | | 16234 | 16304 | | 71 | |  | | GTA | |  | | 13 | | L | |  |  |
| *tRNA-Ser* (*S1*) | | | 16318 | 16388 | | 71 | |  | | TGA | |  | | 1 | | L | |  |  |
| *tRNA-Asp* (*D*) | | | 16390 | 16458 | | 69 | |  | | GTC | |  | | 2 | | H | |  |  |
| *ND6* | | | 16461 | 16982 | | 522 | | 173 | |  | | ATG/TAG | | 0 | | L | |  |  |
| *tRNA-Glu* (*E*) | | | 16983 | 17051 | | 69 | |  | | TTC | |  | | 10 | | L | |  |  |
| *tRNA-Pro* (*F*) | | | 17062 | 17129 | | 68 | |  | | TGG | |  | | 0 | | L | |  |  |
| CR1 | | | 17130 | 18642 | | 1513 | |  | |  | |  | | 0 | | H | |  |  |
| ***Lophonectes gallus***  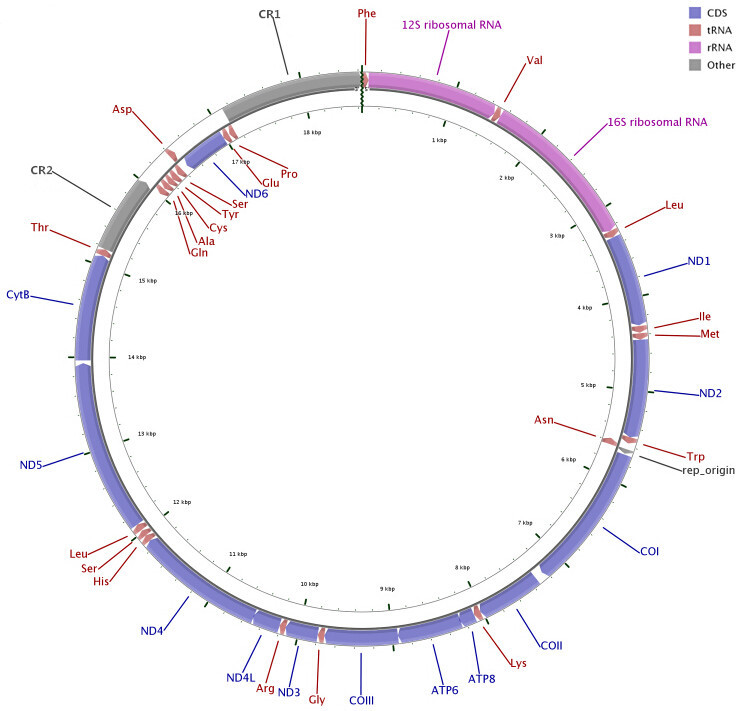  ***Psettina iijimae*** | | | | | | | | | | | | | | | | | |  |  |
| Gene | Position | | | | | Length(bp) | | AA | | Anticondon | | Start/Stop condon | | Intergenic region* | | Strand | |  |  |
|  | From | | | To | |  |  |  |  |  |  |  |  |  |  |  |  |  |  |
| *tRNA-Phe* (*F*) | | | 1 | | | 68 | | 68 | |  | | GAA | |  | | 0 | | H | |
| *12S* | | | 69 | | | 1024 | | 956 | |  | |  | |  | | 0 | | H | |
| *tRNA-Val*(*V*) | | | 1025 | | | 1095 | | 71 | |  | | TAC | |  | | 0 | | H | |
| *16S* | | | 1096 | | | 2804 | | 1709 | |  | |  | |  | | 0 | | H | |
| *tRNA-Leu* (*L1*) | | | 2805 | | | 2878 | | 74 | |  | | TAA | |  | | 1 | | H | |
| *ND1* | | | 2880 | | | 3857 | | 978 | | 325 | |  | | ATG/TAG | | 8 | | H | |
| *tRNA-Ile* (*I*) | | | 3866 | | | 3937 | | 72 | |  | | GAT | |  | | 4 | | H | |
| *tRNA-Met* (*M*) | | | 3942 | | | 4010 | | 69 | |  | | CAT | |  | | 0 | | H | |
| *ND2* | | | 4011 | | | 5060 | | 1050 | | 349 | |  | | ATG/TAG | | -2 | | H | |
| *tRNA-Trp* (*W*) | | | 5059 | | | 5128 | | 70 | |  | | TCA | |  | | 7 | | H | |
| *tRNA-Asn* (*A*) | | | 5136 | | | 5208 | | 73 | |  | | GTT | |  | | -6 | | L | |
| OL | | | 5203 | | | 5252 | | 50 | |  | |  | |  | | 6 | | H | |
| *COI* | | | 5259 | | | 6815 | | 1557 | | 518 | |  | | GTG/TAG | | 74 | | H | |
| *COII* | | | 6890 | | | 7580 | | 691 | | 230 | |  | | ATG/T | | 0 | | H | |
| *tRNA-Lys* (*K*) | | | 7581 | | | 7653 | | 73 | |  | | TTT | |  | | 1 | | H | |
| *ATP8* | | | 7655 | | | 7822 | | 168 | | 55 | |  | | ATG/TAA | | -10 | | H | |
| *ATP6* | | | 7813 | | | 8496 | | 684 | | 227 | |  | | ATG/TAA | | -1 | | H | |
| *COIII* | | | 8496 | | | 9281 | | 786 | | 261 | |  | | ATG/TAA | | -1 | | H | |
| *tRNA-Gly* (*G*) | | | 9281 | | | 9351 | | 71 | |  | | TCC | |  | | 0 | | H | |
| *ND3* | | | 9352 | | | 9702 | | 351 | | 116 | |  | | ATG/TAG | | -2 | | H | |
| *tRNA-Arg* (*R*) | | | 9701 | | | 9769 | | 69 | |  | | TCG | |  | | 0 | | H | |
| *ND4L* | | | 9770 | | | 10066 | | 297 | | 98 | |  | | ATG/TAA | | -7 | | H | |
| *ND4* | | | 10060 | | | 11440 | | 1381 | | 460 | |  | | ATG/T | | 0 | | H | |
| *tRNA-His* (*H*) | | | 11441 | | | 11508 | | 68 | |  | | GTG | |  | | 0 | | H | |
| *tRNA-Ser* (*S2*) | | | 11509 | | | 11575 | | 67 | |  | | GCT | |  | | 7 | | H | |
| *tRNA-Leu* (*L2*) | | | 11583 | | | 11655 | | 73 | |  | | TAG | |  | | 0 | | H | |
| *ND5* | | | 11656 | | | 13494 | | 1839 | | 612 | |  | | ATG/TAA | | 16 | | H | |
| *CytB* | | | 13511 | | | 14651 | | 1141 | | 380 | |  | | ATG/T | | 0 | | H | |
| *tRNA-Thr* (*T*) | | | 14652 | | | 14723 | | 72 | |  | | TGT | |  | | 0 | | H | |
| CR2 | | | 14724 | | | 15559 | | 836 | |  | |  | |  | | 0 | | H | |
| *tRNA-Gln* (*Q*) | | | 15560 | | | 15630 | | 71 | |  | | TTG | |  | | 3 | | L | |
| *tRNA-Ala* (*A*) | | | 15634 | | | 15702 | | 69 | |  | | TGC | |  | | 5 | | L | |
| *tRNA-Cys* (*C*) | | | 15708 | | | 15773 | | 66 | |  | | GCA | |  | | 0 | | L | |
| *tRNA-Tyr* (*Y*) | | | 15774 | | | 15844 | | 71 | |  | | GTA | |  | | 1 | | L | |
| *tRNA-Ser* (*S1*) | | | 15846 | | | 15916 | | 71 | |  | | TGA | |  | | 2 | | L | |
| *tRNA-Asp* (*D*) | | | 15919 | | | 15987 | | 69 | |  | | GTC | |  | | -3 | | H | |
| *ND6* | | | 15985 | | | 16506 | | 522 | | 173 | |  | | ATG/TAA | | 0 | | L | |
| *tRNA-Glu* (*E*) | | | 16507 | | | 16574 | | 68 | |  | | TTC | |  | | 2 | | L | |
| *tRNA-Pro* (*F*) | | | 16577 | | | 16644 | | 68 | |  | | TGG | |  | | 0 | | L | |
| CR1 | | | 16645 | | | 18080 | | 1436 | |  | |  | |  | | 0 | | H | |
| *Intergenic region: non-coding bases between the feature on the same line and the line below, with a negative number indicating an overlap. | | | | | | | | | | | | | | | | | |  |  |

***Psettina iijimae***


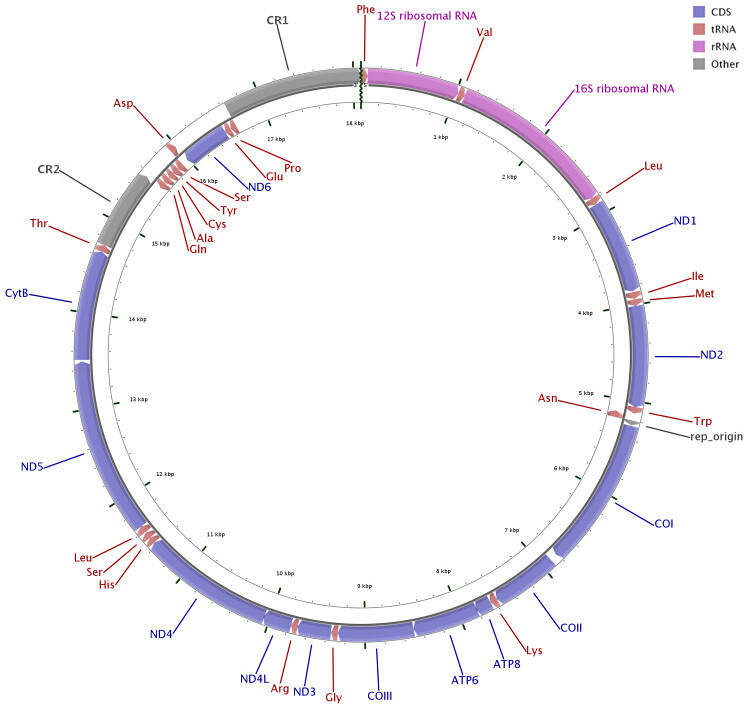

Supplement: S2 Table — Intergenic region: non-coding bases between the feature on the same line and the line below, with a negative number indicating an overlap. (DOCX) [file pone.0134580.s004.docx]
