# Supplementary material for: Concerted Evolution of Duplicate Control Regions in the Mitochondria of Species of the Flatfish Family Bothidae (Teleostei: Pleuronectiformes)
Source: PLoS One. 2015 Aug 3;10(8):e0134580. doi: 10.1371/journal.pone.0134580 (PMC4523187; doi:10.1371/journal.pone.0134580)
Supplement: S3 Table — Abbreviations of species names are shown in Table 1. Numbers following the species names represent the type of CRs: CR1 or CR2. Numbers in gray are sequence similarity and the genetic distances of Kimura-2 Parameter between core regions of the duplicate CRs within each of four species of flounders. (DOCX) [file pone.0134580.s005.docx]

**S3 Table. Percent similarity (above triangle) and genetic distance (below triangle) calculated by Kimura-2 Parameter based on**

**core regions of the CRs and counterpart sepuences from 11 bothids with two other flatfishes as outgroup.***

| Species | A.te1 | A.te2 | L.ga1 | L.ga2 | L.la1 | L.la2 | P.ii1 | P.ii2 | A.po2 | B.my2 | B.pa1 | C.az2 | C.ko2 | C.va2 | C.lu2 | P.ste | P.oli |
| --- | --- | --- | --- | --- | --- | --- | --- | --- | --- | --- | --- | --- | --- | --- | --- | --- | --- |
| A.te1 |  | 100 | 70.8 | 70.8 | 65.4 | 65.4 | 69.5 | 69.4 | 66.8 | 53.3 | 64.0 | 62.1 | 61.9 | 59.1 | 52.1 | 60.3 | 60.4 |
| A.te2 | 0 |  | 70.8 | 70.8 | 65.4 | 65.4 | 69.5 | 69.4 | 66.8 | 53.3 | 64.0 | 62.1 | 61.9 | 59.1 | 52.1 | 60.3 | 60.4 |
| L.ga1 | 0.356 | 0.356 |  | 100 | 66.3 | 66.3 | 76.0 | 76.1 | 74.6 | 56.1 | 66.3 | 61.0 | 60.7 | 64.0 | 54.8 | 64.6 | 63.6 |
| L.ga2 | 0.356 | 0.356 | 0 |  | 66.3 | 66.3 | 76.0 | 76.1 | 74.6 | 56.1 | 66.3 | 61.0 | 60.7 | 64.0 | 54.8 | 64.6 | 63.6 |
| L.la1 | 0.436 | 0.436 | 0.418 | 0.418 |  | 100 | 66.3 | 66.5 | 65.6 | 54.5 | 65.7 | 58.2 | 57.6 | 58.5 | 55.3 | 62.2 | 62.8 |
| L.la2 | 0.436 | 0.436 | 0.418 | 0.418 | 0 |  | 66.3 | 66.5 | 65.6 | 54.5 | 65.7 | 58.2 | 57.6 | 58.5 | 55.3 | 62.2 | 62.8 |
| P.ii1 | 0.356 | 0.356 | 0.241 | 0.241 | 0.426 | 0.426 |  | 99.8 | 70.4 | 54.5 | 64.8 | 63.1 | 61.1 | 66.2 | 56.5 | 63.9 | 63.4 |
| P.ii2 | 0.356 | 0.356 | 0.238 | 0.238 | 0.422 | 0.422 | 0.002 |  | 70.4 | 54.5 | 65.0 | 63.1 | 61.1 | 66.2 | 56.4 | 64.1 | 63.5 |
| A.po2 | 0.373 | 0.373 | 0.265 | 0.265 | 0.429 | 0.429 | 0.326 | 0.326 |  | 56.4 | 65.1 | 62.7 | 60.2 | 62.4 | 56.9 | 63.2 | 61.7 |
| B.my2 | 0.780 | 0.780 | 0.645 | 0.645 | 0.672 | 0.672 | 0.625 | 0.625 | 0.591 |  | 57.4 | 51.4 | 50.2 | 53.2 | 64.6 | 55.4 | 60.0 |
| B.pa1 | 0.398 | 0.398 | 0.394 | 0.394 | 0.428 | 0.428 | 0.428 | 0.428 | 0.380 | 0.610 |  | 60.5 | 58.6 | 58.9 | 54.4 | 61.1 | 59.3 |
| C.az2 | 0.411 | 0.411 | 0.475 | 0.475 | 0.589 | 0.589 | 0.407 | 0.407 | 0.432 | 0.710 | 0.506 |  | 66.3 | 65.2 | 51.1 | 55.8 | 57.6 |
| C.ko2 | 0.485 | 0.485 | 0.513 | 0.513 | 0.635 | 0.635 | 0.472 | 0.472 | 0.486 | 0.774 | 0.540 | 0.367 |  | 62.5 | 51.2 | 56.0 | 55.2 |
| C.va2 | 0.543 | 0.543 | 0.424 | 0.424 | 0.582 | 0.582 | 0.389 | 0.389 | 0.476 | 0.693 | 0.516 | 0.428 | 0.496 |  | 52.2 | 54.8 | 55.3 |
| C.lu2 | 0.742 | 0.742 | 0.622 | 0.622 | 0.666 | 0.666 | 0.591 | 0.591 | 0.575 | 0.446 | 0.622 | 0.705 | 0.728 | 0.640 |  | 56.2 | 62.4 |
| P.ste | 0.544 | 0.544 | 0.471 | 0.471 | 0.504 | 0.504 | 0.448 | 0.444 | 0.460 | 0.621 | 0.488 | 0.582 | 0.645 | 0.635 | 0.635 |  | 74.7 |
| P.oli | 0.535 | 0.535 | 0.417 | 0.417 | 0.444 | 0.444 | 0.417 | 0.417 | 0.444 | 0.523 | 0.459 | 0.573 | 0.640 | 0.590 | 0.464 | 0.308 |  |

* Abbreviations of the species names are shown in Table 1. Numbers following the species names represent the type of CRs: CR1 or CR2. Numbers in gray are sequence similarity and the genetic distances of Kimura-2 Parameter between core regions of the duplicate CRs within each of four species of flounders.
